# Supplementary material for: Global, Regional, and National Burdens with Temporal Trends of Early-, Intermediate-, and Later-Onset Gastric Cancer from 1990 to 2019 and Predictions up to 2035
Source: Cancers (Basel). 2022 Nov 3;14(21):5417. doi: 10.3390/cancers14215417 (PMC9656932; doi:10.3390/cancers14215417)
Supplement: Supplementary file 1 [file cancers-14-05417-s001.zip › Supplementary Figures.pdf]

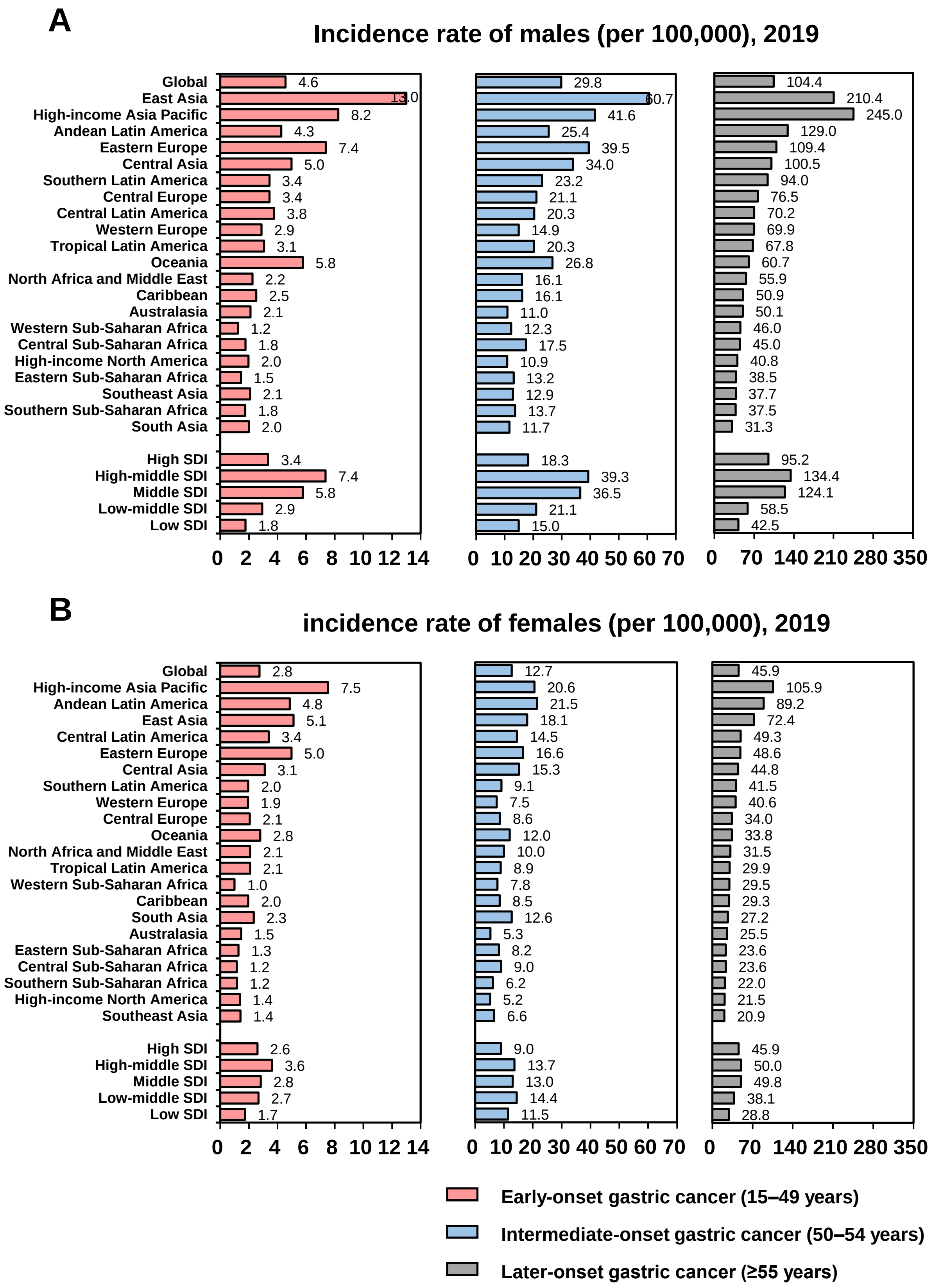
A
Incidence rate of males (per 100,000), 2019
Global 4.6 29.8 104.4
East Asia 13.0 60.7 210.4
High-income Asia Pacific 8.2 41.6 245.0
Andean Latin America 4.3 25.4 129.0
Eastern Europe 7.4 39.5 109.4
Central Asia 5.0 34.0 100.5
Southern Latin America 3.4 23.2 94.0
Central Europe 3.4 21.1 76.5
Central Latin America 3.8 20.3 70.2
Western Europe 2.9 14.9 69.9
Tropical Latin America 3.1 20.3 67.8
Oceania 5.8 26.8 60.7
North Africa and Middle East 2.2 16.1 55.9
Caribbean 2.5 16.1 50.9
Australasia 2.1 11.0 50.1
Western Sub-Saharan Africa 1.2 12.3 46.0
Central Sub-Saharan Africa 1.8 17.5 45.0
High-income North America 2.0 10.9 40.8
Eastern Sub-Saharan Africa 1.5 13.2 38.5
Southeast Asia 2.1 12.9 37.7
Southern Sub-Saharan Africa 1.8 13.7 37.5
South Asia 2.0 11.7 31.3
High SDI 3.4 18.3 95.2
High-middle SDI 7.4 39.3 134.4
Middle SDI 5.8 36.5 124.1
Low-middle SDI 2.9 21.1 58.5
Low SDI 1.8 15.0 42.5
0 2 4 6 8 10 12 14
0 10 20 30 40 50 60 70
0 70 140 210 280 350
B
incidence rate of females (per 100,000), 2019
Global 2.8 12.7 45.9
High-income Asia Pacific 7.5 20.6 105.9
Andean Latin America 4.8 21.5 89.2
East Asia 5.1 18.1 72.4
Central Latin America 3.4 14.5 49.3
Eastern Europe 5.0 16.6 48.6
Central Asia 3.1 15.3 44.8
Southern Latin America 2.0 9.1 41.5
Western Europe 1.9 7.5 40.6
Central Europe 2.1 8.6 34.0
Oceania 2.8 12.0 33.8
North Africa and Middle East 2.1 10.0 31.5
Tropical Latin America 2.1 8.9 29.9
Western Sub-Saharan Africa 1.0 7.8 29.5
Caribbean 2.0 8.5 29.3
South Asia 2.3 12.6 27.2
Australasia 1.5 5.3 25.5
Eastern Sub-Saharan Africa 1.3 8.2 23.6
Central Sub-Saharan Africa 1.2 9.0 23.6
Southern Sub-Saharan Africa 1.2 6.2 22.0
High-income North America 1.4 5.2 21.5
Southeast Asia 1.4 6.6 20.9
High SDI 2.6 9.0 45.9
High-middle SDI 3.6 13.7 50.0
Middle SDI 2.8 13.0 49.8
Low-middle SDI 2.7 14.4 38.1
Low SDI 1.7 11.5 28.8
0 2 4 6 8 10 12 14
0 10 20 30 40 50 60 70
0 70 140 210 280 350
Early-onset gastric cancer (15–49 years)
Intermediate-onset gastric cancer (50–54 years)
Later-onset gastric cancer (≥55 years)

Figure S1

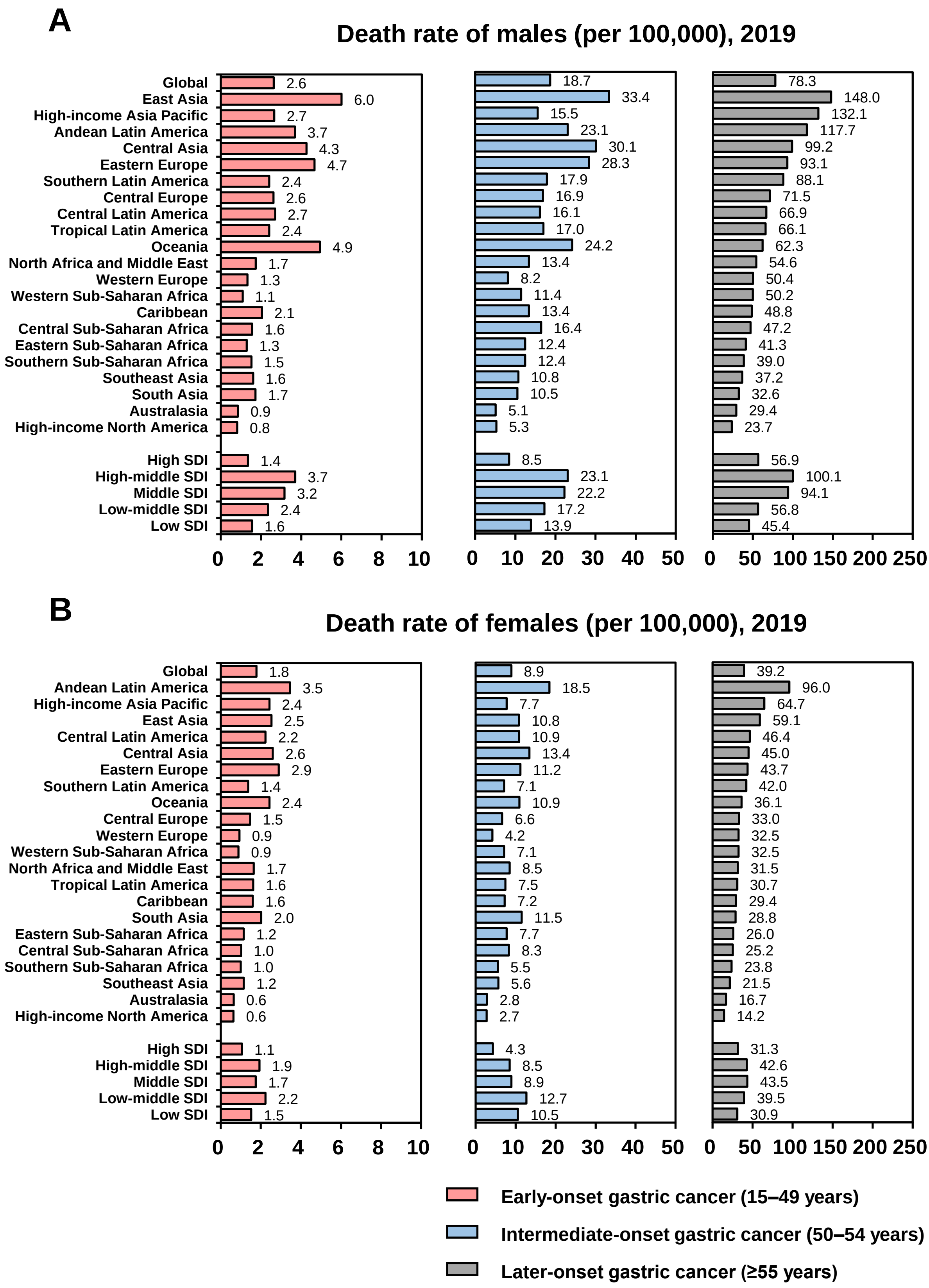
A
Death rate of males (per 100,000), 2019
Global 2.6 18.7 78.3
East Asia 6.0 33.4 148.0
High-income Asia Pacific 2.7 15.5 132.1
Andean Latin America 3.7 23.1 117.7
Central Asia 4.3 30.1 99.2
Eastern Europe 4.7 28.3 93.1
Southern Latin America 2.4 17.9 88.1
Central Europe 2.6 16.9 71.5
Central Latin America 2.7 16.1 66.9
Tropical Latin America 2.4 17.0 66.1
Oceania 4.9 24.2 62.3
North Africa and Middle East 1.7 13.4 54.6
Western Europe 1.3 8.2 50.4
Western Sub-Saharan Africa 1.1 11.4 50.2
Caribbean 2.1 13.4 48.8
Central Sub-Saharan Africa 1.6 16.4 47.2
Eastern Sub-Saharan Africa 1.3 12.4 41.3
Southern Sub-Saharan Africa 1.5 12.4 39.0
Southeast Asia 1.6 10.8 37.2
South Asia 1.7 10.5 32.6
Australasia 0.9 5.1 29.4
High-income North America 0.8 5.3 23.7
High SDI 1.4 8.5 56.9
High-middle SDI 3.7 23.1 100.1
Middle SDI 3.2 22.2 94.1
Low-middle SDI 2.4 17.2 56.8
Low SDI 1.6 13.9 45.4
0 2 4 6 8 10
0 10 20 30 40 50
0 50 100 150 200 250
B
Death rate of females (per 100,000), 2019
Global 1.8 8.9 39.2
Andean Latin America 3.5 18.5 96.0
High-income Asia Pacific 2.4 7.7 64.7
East Asia 2.5 10.8 59.1
Central Latin America 2.2 10.9 46.4
Central Asia 2.6 13.4 45.0
Eastern Europe 2.9 11.2 43.7
Southern Latin America 1.4 7.1 42.0
Oceania 2.4 10.9 36.1
Central Europe 1.5 6.6 33.0
Western Europe 0.9 4.2 32.5
Western Sub-Saharan Africa 0.9 7.1 32.5
North Africa and Middle East 1.7 8.5 31.5
Tropical Latin America 1.6 7.5 30.7
Caribbean 1.6 7.2 29.4
South Asia 2.0 11.5 28.8
Eastern Sub-Saharan Africa 1.2 7.7 26.0
Central Sub-Saharan Africa 1.0 8.3 25.2
Southern Sub-Saharan Africa 1.0 5.5 23.8
Southeast Asia 1.2 5.6 21.5
Australasia 0.6 2.8 16.7
High-income North America 0.6 2.7 14.2
High SDI 1.1 4.3 31.3
High-middle SDI 1.9 8.5 42.6
Middle SDI 1.7 8.9 43.5
Low-middle SDI 2.2 12.7 39.5
Low SDI 1.5 10.5 30.9
0 2 4 6 8 10
0 10 20 30 40 50
0 50 100 150 200 250
Early-onset gastric cancer (15–49 years)
Intermediate-onset gastric cancer (50–54 years)
Later-onset gastric cancer (≥55 years)

Figure S2

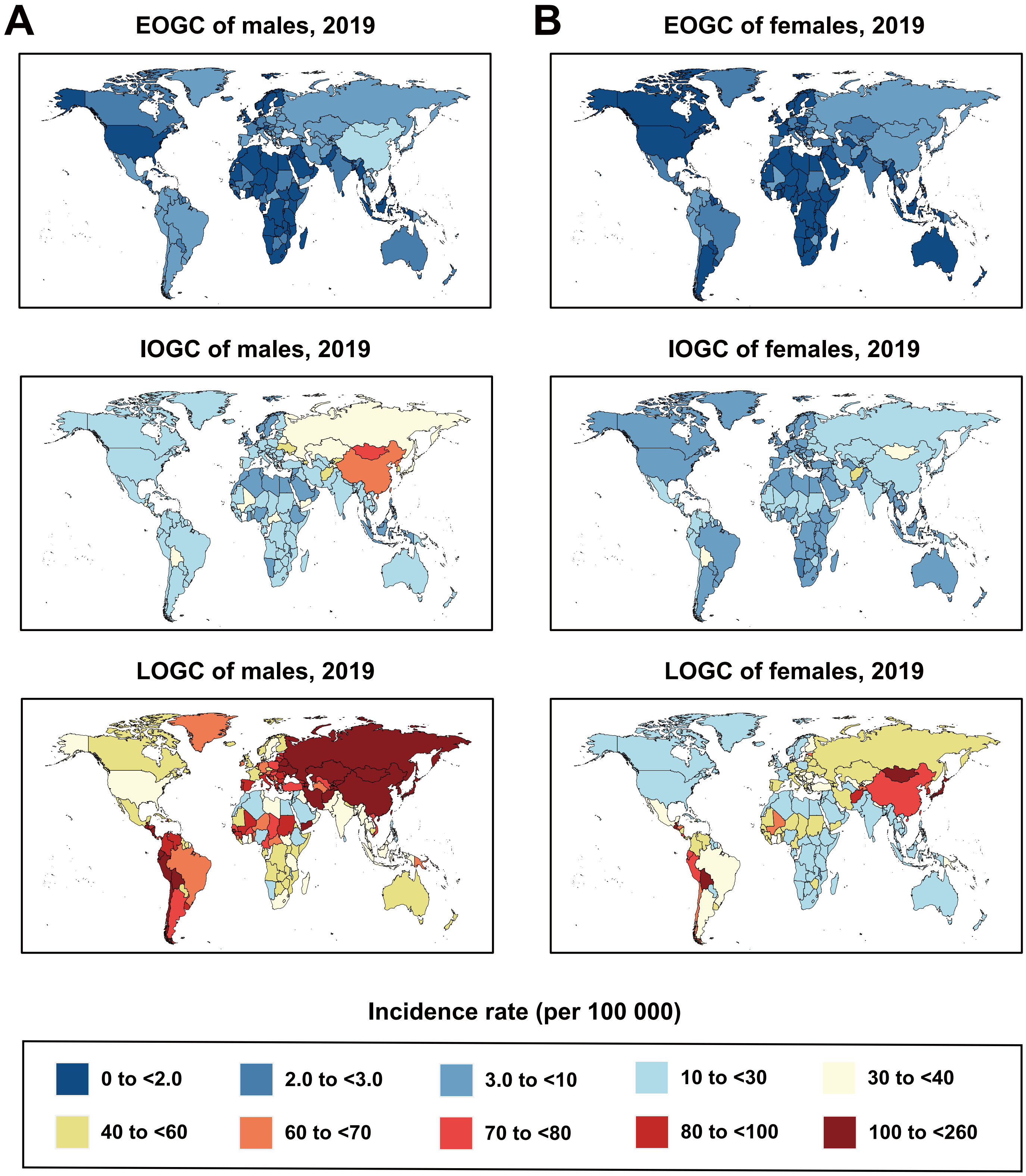
A
EOGC of males, 2019
B
EOGC of females, 2019
IOGC of males, 2019
IOGC of females, 2019
LOGC of males, 2019
LOGC of females, 2019
Incidence rate (per 100 000)
0 to <2.0
2.0 to <3.0
3.0 to <10
10 to <30
30 to <40
40 to <60
60 to <70
70 to <80
80 to <100
100 to <260

Figure S3

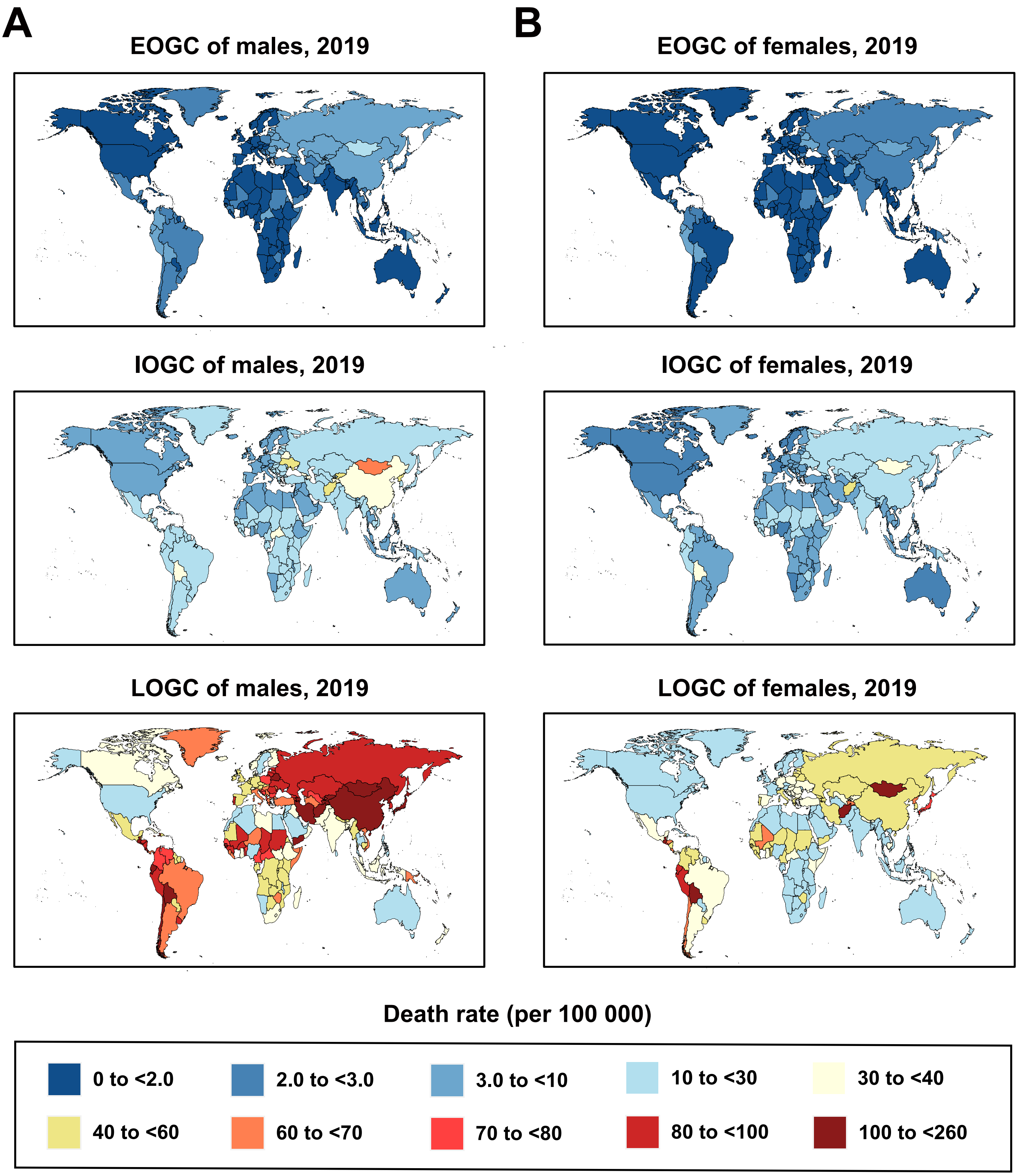
A
EOGC of males, 2019
B
EOGC of females, 2019
IOGC of males, 2019
IOGC of females, 2019
LOGC of males, 2019
LOGC of females, 2019
Death rate (per 100 000)
0 to <2.0
2.0 to <3.0
3.0 to <10
10 to <30
30 to <40
40 to <60
60 to <70
70 to <80
80 to <100
100 to <260

Figure S4

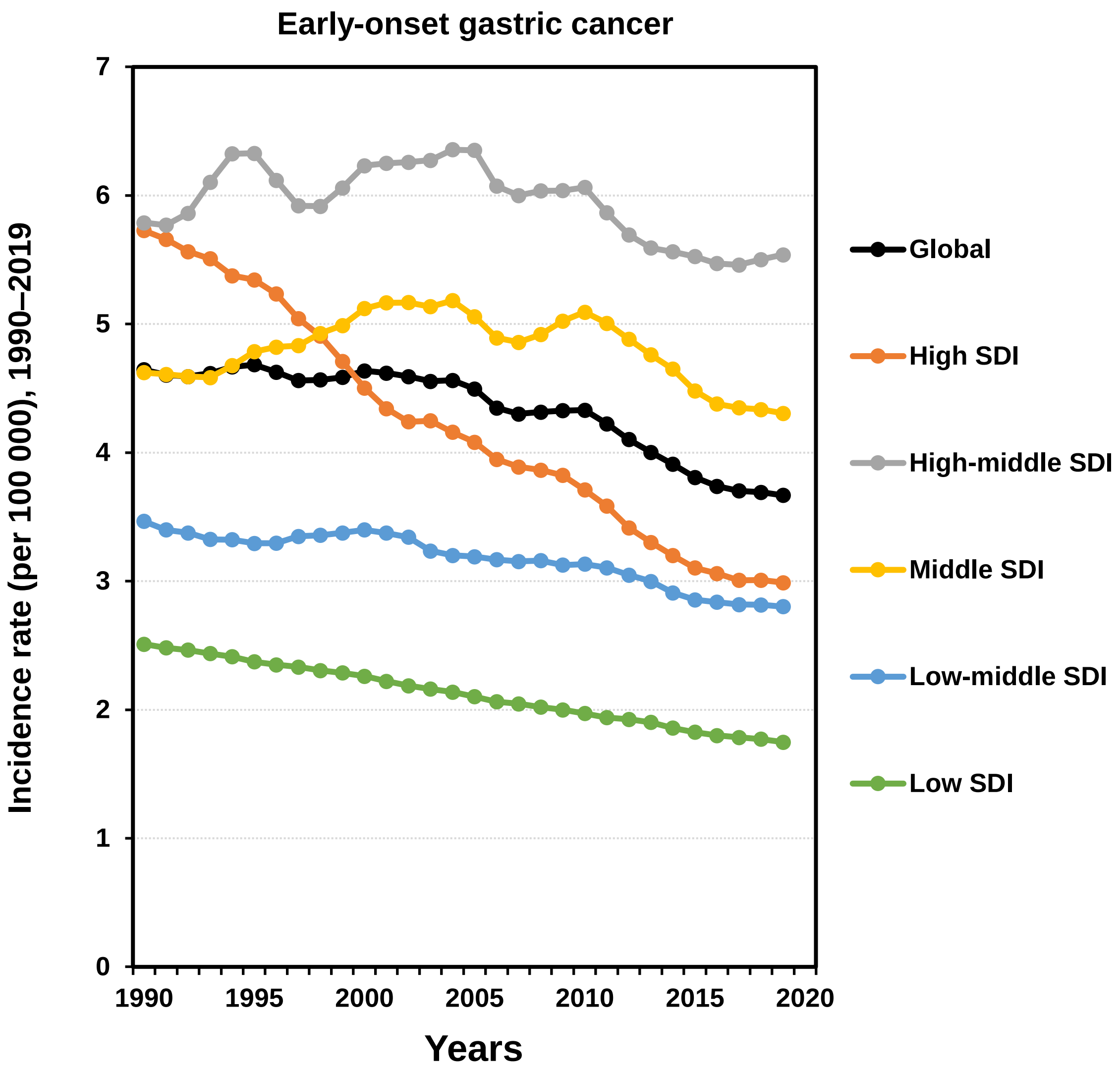
Early-onset gastric cancer
Incidence rate (per 100 000), 1990–2019
0
1
2
3
4
5
6
7
1990
1995
2000
2005
2010
2015
2020
Years
Global
High SDI
High-middle SDI
Middle SDI
Low-middle SDI
Low SDI

Figure S5

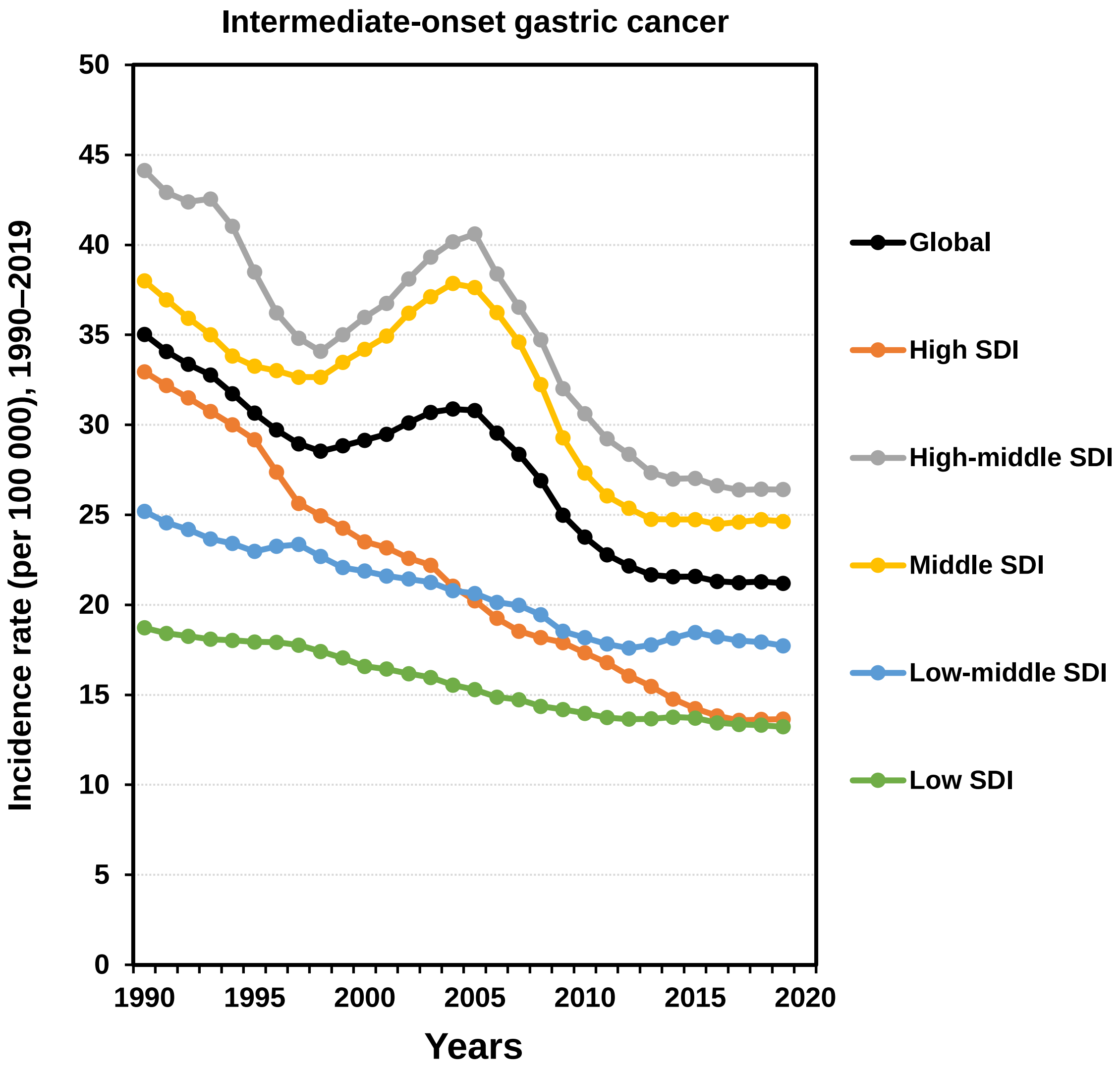

Intermediate-onset gastric cancer
Incidence rate (per 100 000), 1990–2019
50
45
40
35
30
25
20
15
10
5
0
1990
1995
2000
2005
2010
2015
2020
Years
Global
High SDI
High-middle SDI
Middle SDI
Low-middle SDI
Low SDI

Figure S6

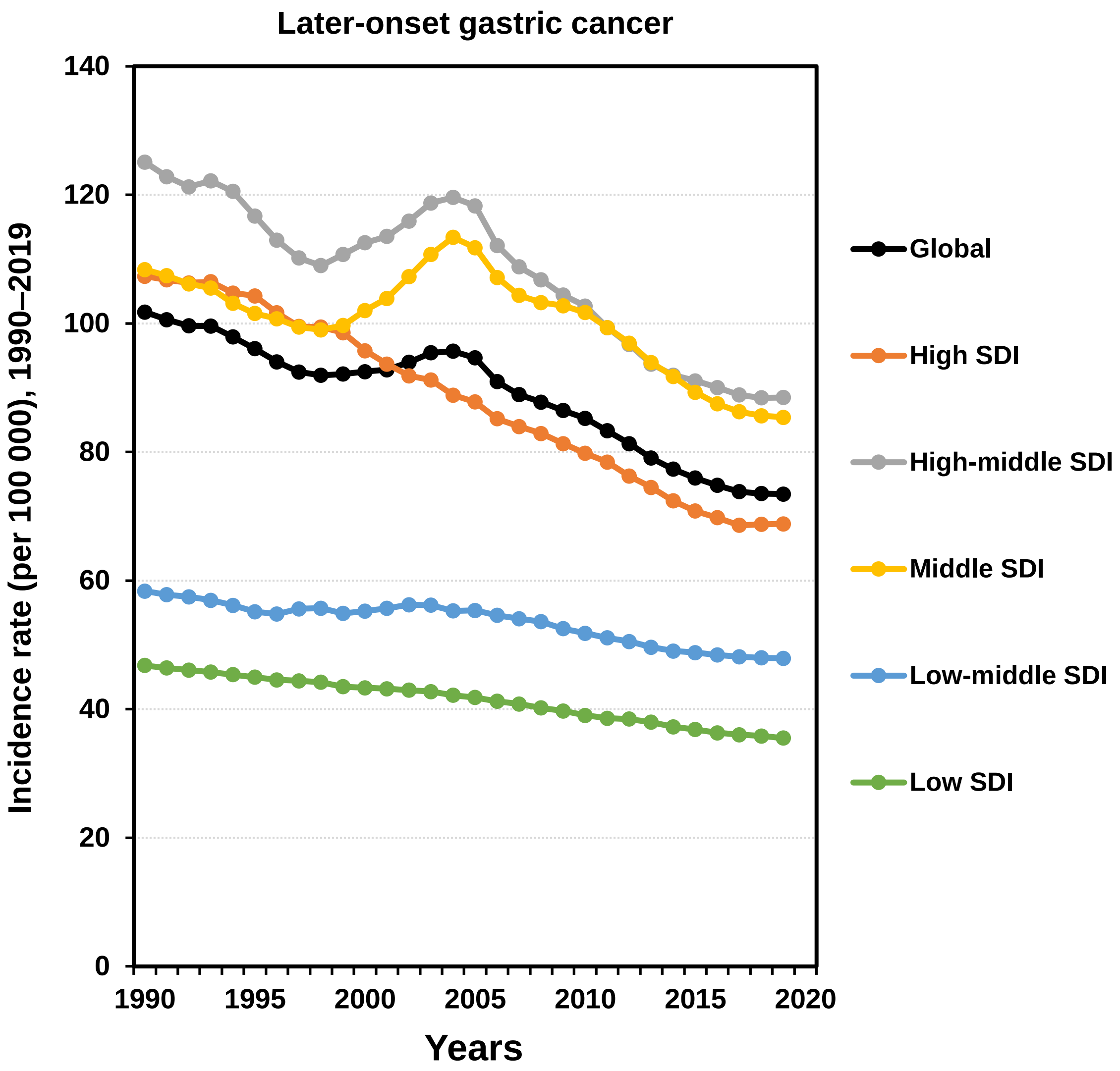
Later-onset gastric cancer
Incidence rate (per 100 000), 1990–2019
Years
0
20
40
60
80
100
120
140
1990
1995
2000
2005
2010
2015
2020
Global
High SDI
High-middle SDI
Middle SDI
Low-middle SDI
Low SDI

Figure S7

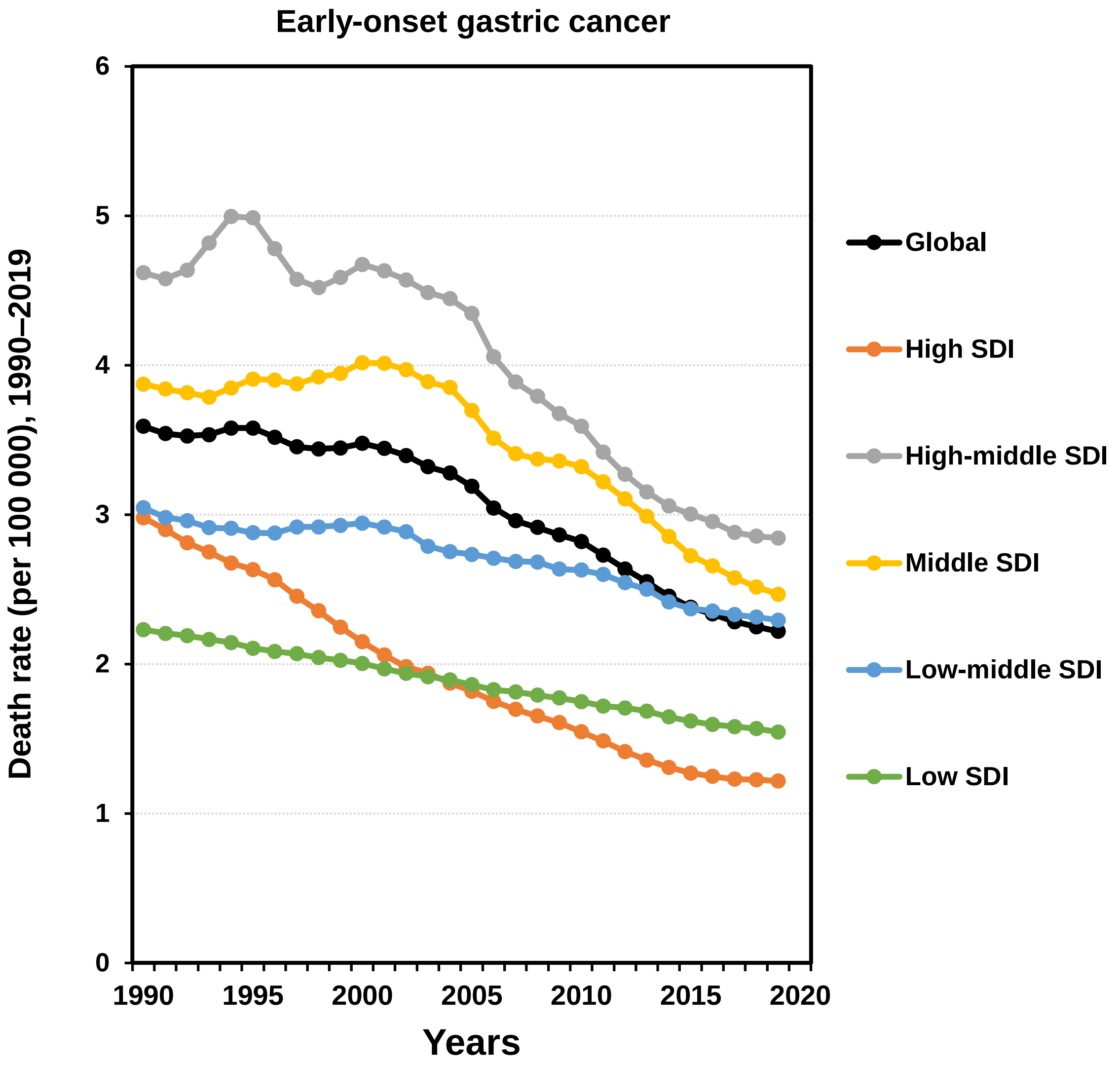

Early-onset gastric cancer
Death rate (per 100 000), 1990–2019
6
5
4
3
2
1
0
1990
1995
2000
2005
2010
2015
2020
Years
Global
High SDI
High-middle SDI
Middle SDI
Low-middle SDI
Low SDI

Figure S8

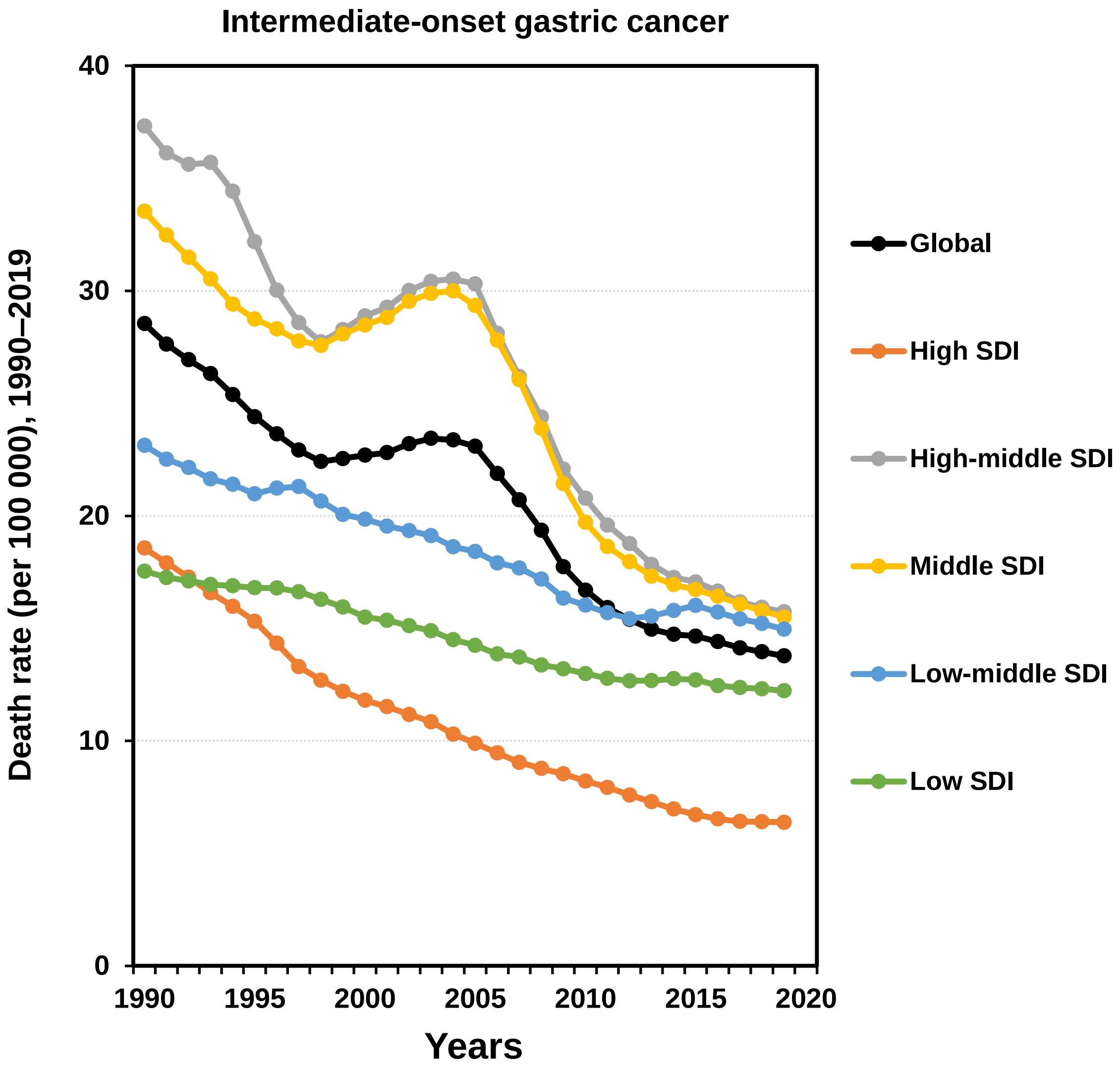

Intermediate-onset gastric cancer
Death rate (per 100 000), 1990–2019
40
30
20
10
0
1990
1995
2000
2005
2010
2015
2020
Years
Global
High SDI
High-middle SDI
Middle SDI
Low-middle SDI
Low SDI

Figure S9

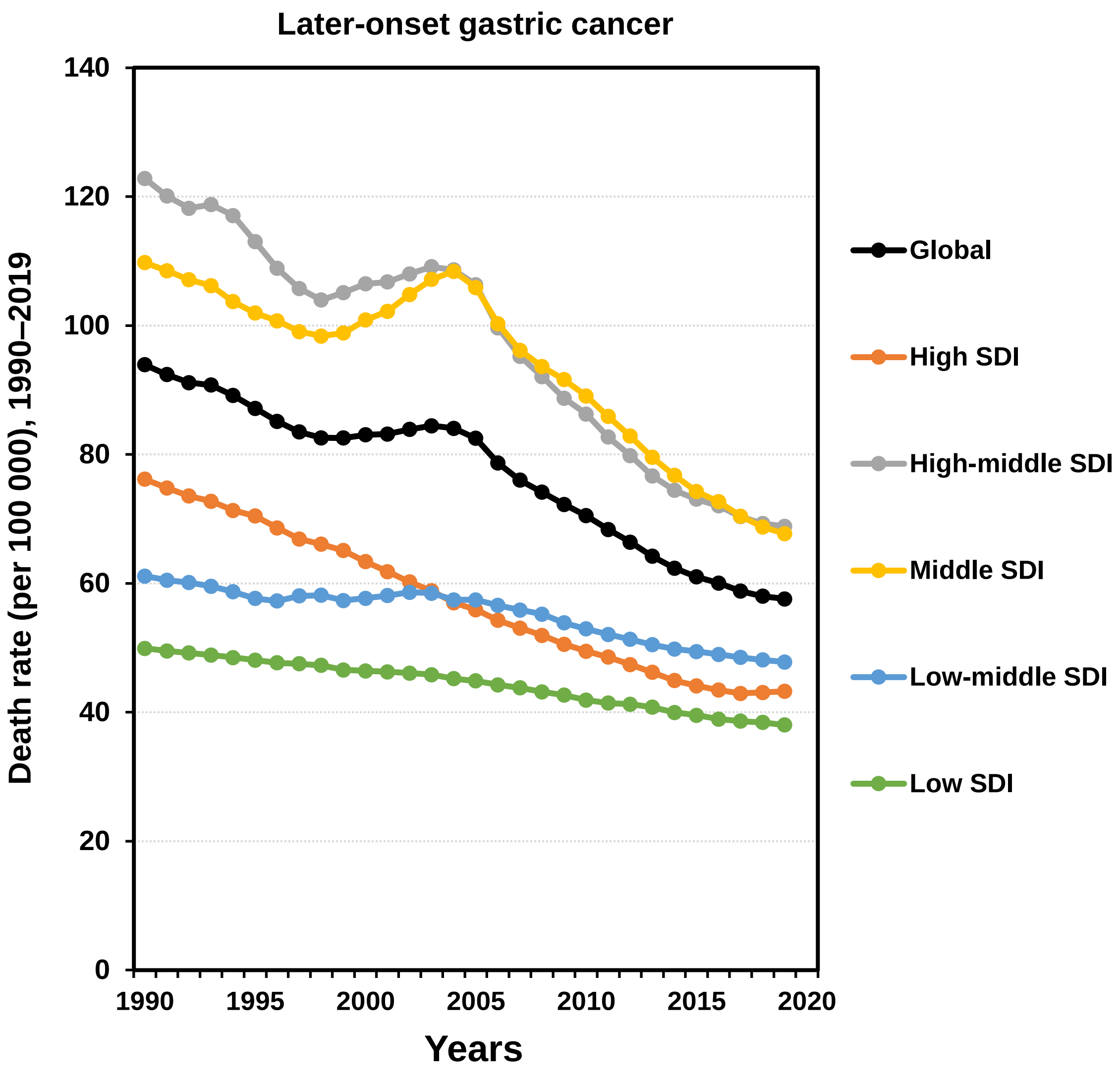
Later-onset gastric cancer
Death rate (per 100 000), 1990–2019
140
120
100
80
60
40
20
0
1990
1995
2000
2005
2010
2015
2020
Years
Global
High SDI
High-middle SDI
Middle SDI
Low-middle SDI
Low SDI

Figure S10

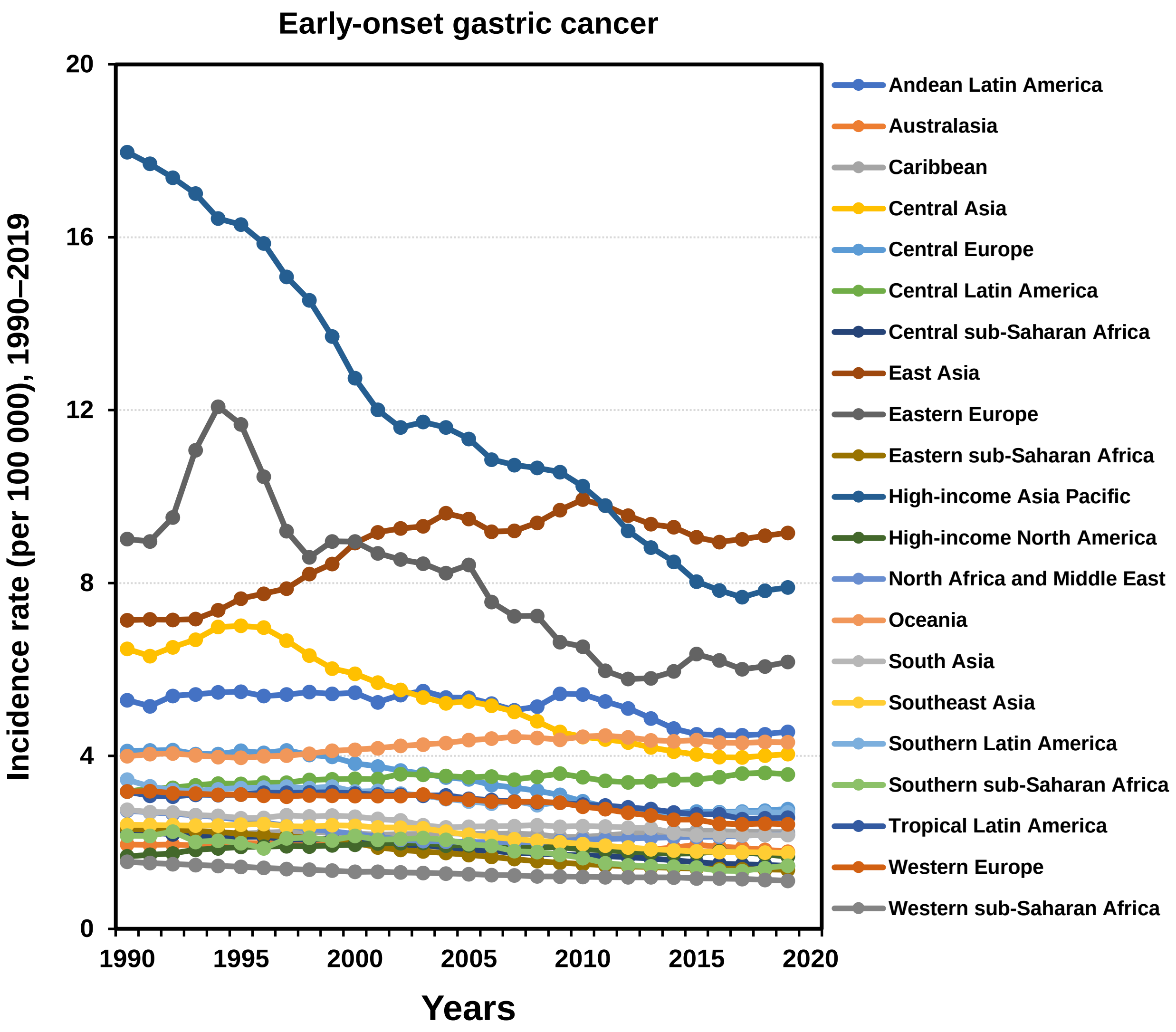

Early-onset gastric cancer
Incidence rate (per 100 000), 1990–2019
Years
0
4
8
12
16
20
1990
1995
2000
2005
2010
2015
2020
Andean Latin America
Australasia
Caribbean
Central Asia
Central Europe
Central Latin America
Central sub-Saharan Africa
East Asia
Eastern Europe
Eastern sub-Saharan Africa
High-income Asia Pacific
High-income North America
North Africa and Middle East
Oceania
South Asia
Southeast Asia
Southern Latin America
Southern sub-Saharan Africa
Tropical Latin America
Western Europe
Western sub-Saharan Africa

Figure S11

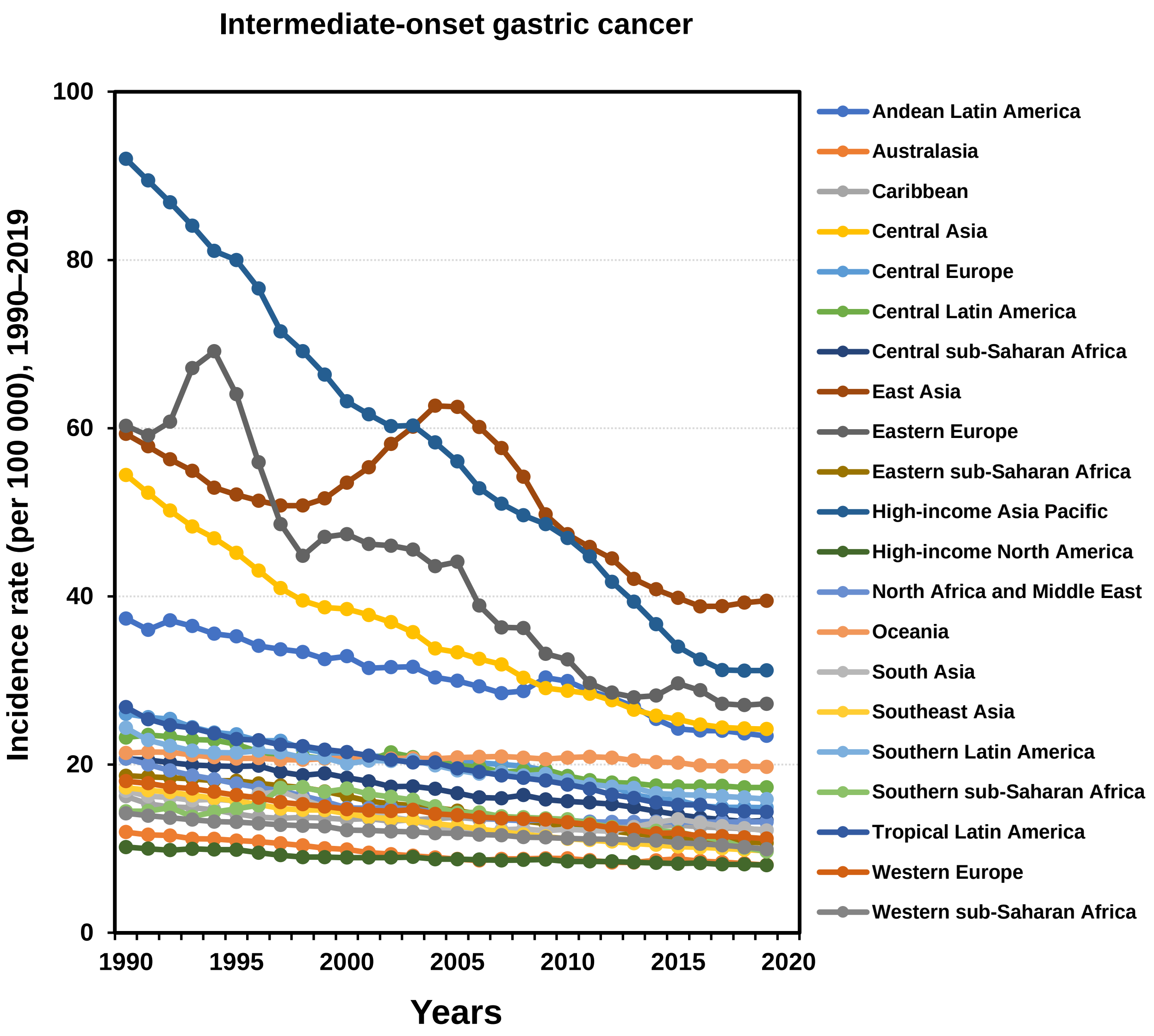
Intermediate-onset gastric cancer
Incidence rate (per 100 000), 1990–2019
100
80
60
40
20
0
1990
1995
2000
2005
2010
2015
2020
Years
Andean Latin America
Australasia
Caribbean
Central Asia
Central Europe
Central Latin America
Central sub-Saharan Africa
East Asia
Eastern Europe
Eastern sub-Saharan Africa
High-income Asia Pacific
High-income North America
North Africa and Middle East
Oceania
South Asia
Southeast Asia
Southern Latin America
Southern sub-Saharan Africa
Tropical Latin America
Western Europe
Western sub-Saharan Africa

Figure S12

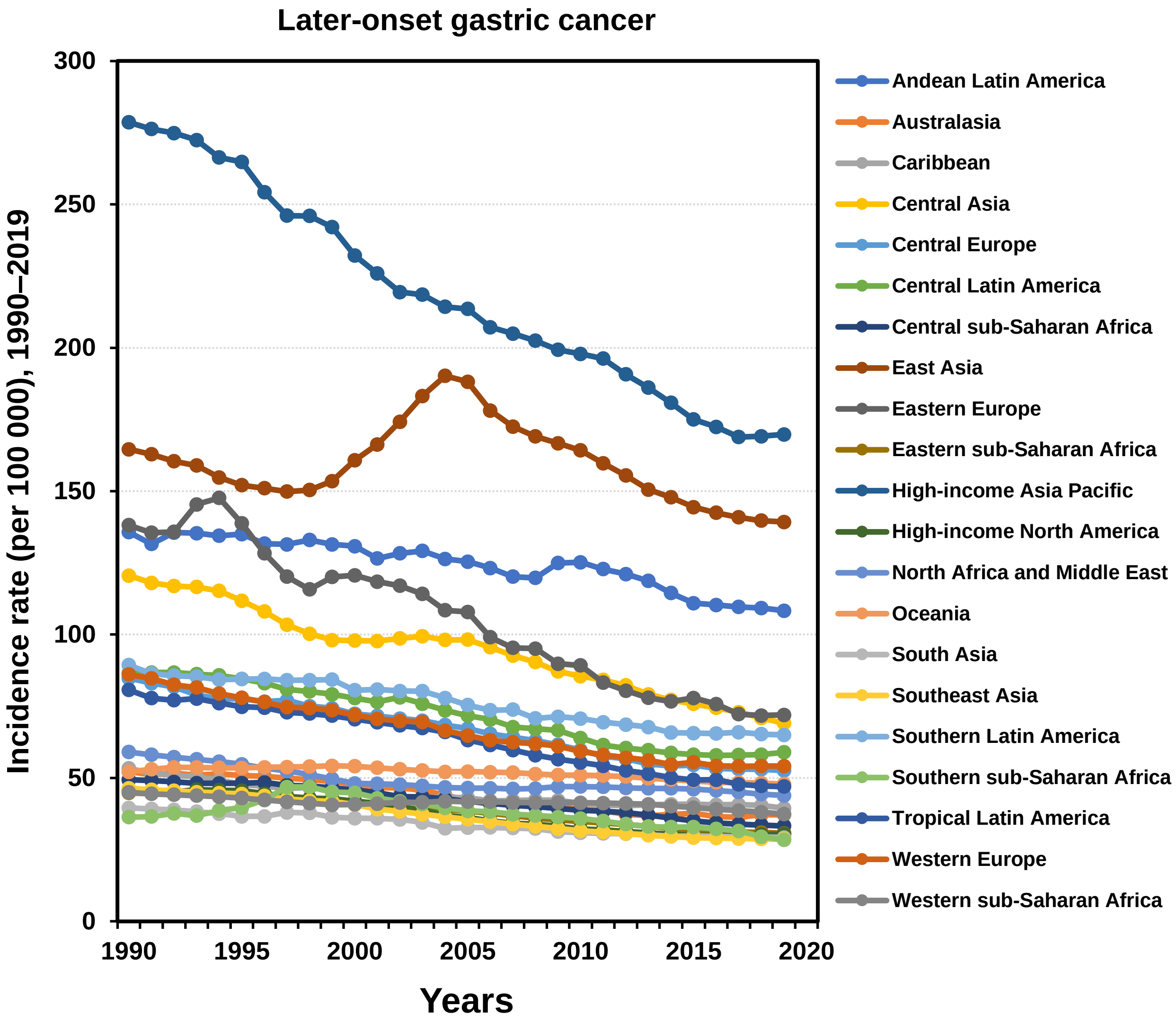

Later-onset gastric cancer
Incidence rate (per 100 000), 1990–2019
Years
0
50
100
150
200
250
300
1990
1995
2000
2005
2010
2015
2020
Andean Latin America
Australasia
Caribbean
Central Asia
Central Europe
Central Latin America
Central sub-Saharan Africa
East Asia
Eastern Europe
Eastern sub-Saharan Africa
High-income Asia Pacific
High-income North America
North Africa and Middle East
Oceania
South Asia
Southeast Asia
Southern Latin America
Southern sub-Saharan Africa
Tropical Latin America
Western Europe
Western sub-Saharan Africa

Figure S13

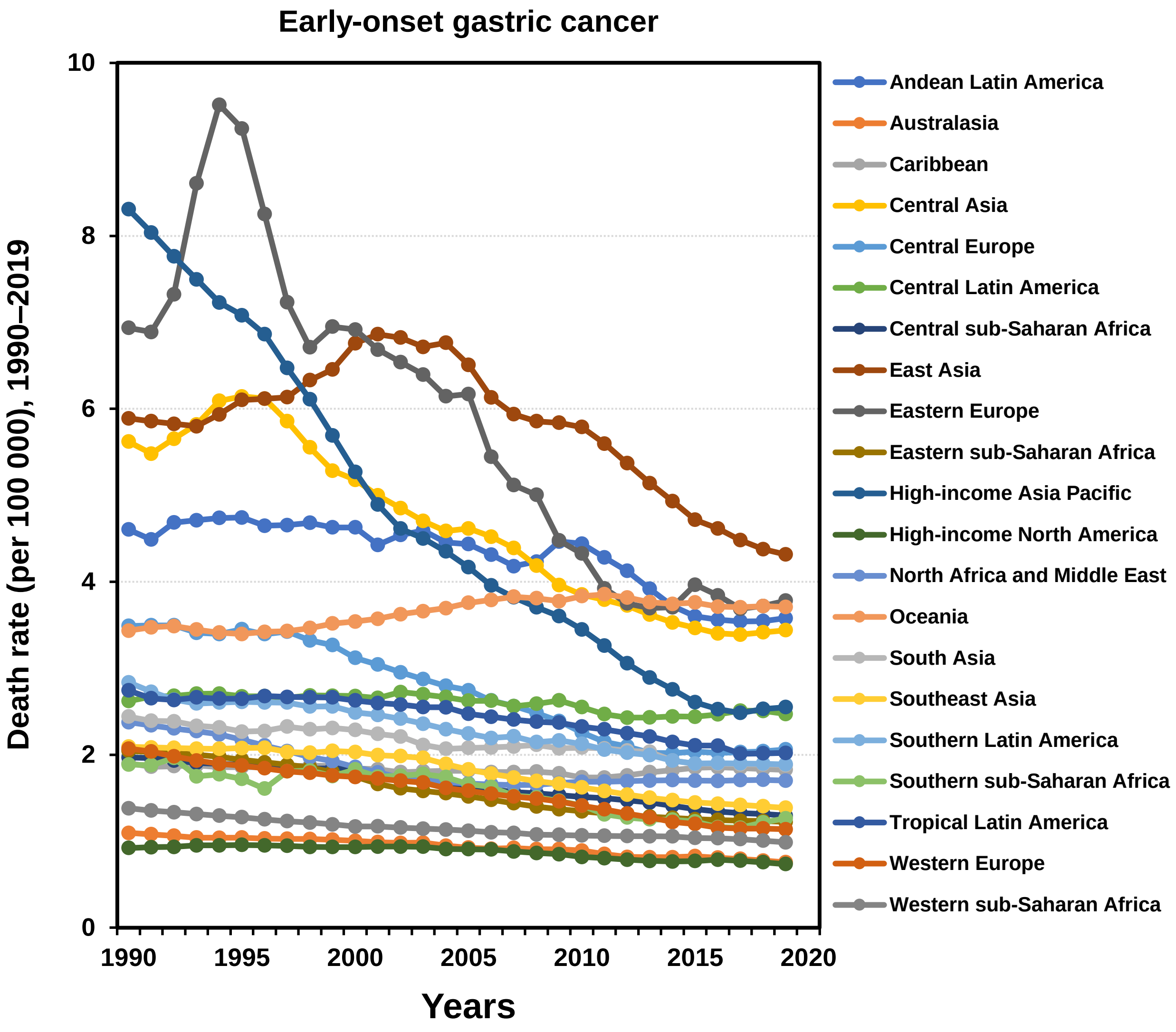

Early-onset gastric cancer
Death rate (per 100 000), 1990–2019
Years
0
2
4
6
8
10
1990
1995
2000
2005
2010
2015
2020
Andean Latin America
Australasia
Caribbean
Central Asia
Central Europe
Central Latin America
Central sub-Saharan Africa
East Asia
Eastern Europe
Eastern sub-Saharan Africa
High-income Asia Pacific
High-income North America
North Africa and Middle East
Oceania
South Asia
Southeast Asia
Southern Latin America
Southern sub-Saharan Africa
Tropical Latin America
Western Europe
Western sub-Saharan Africa

Figure S14

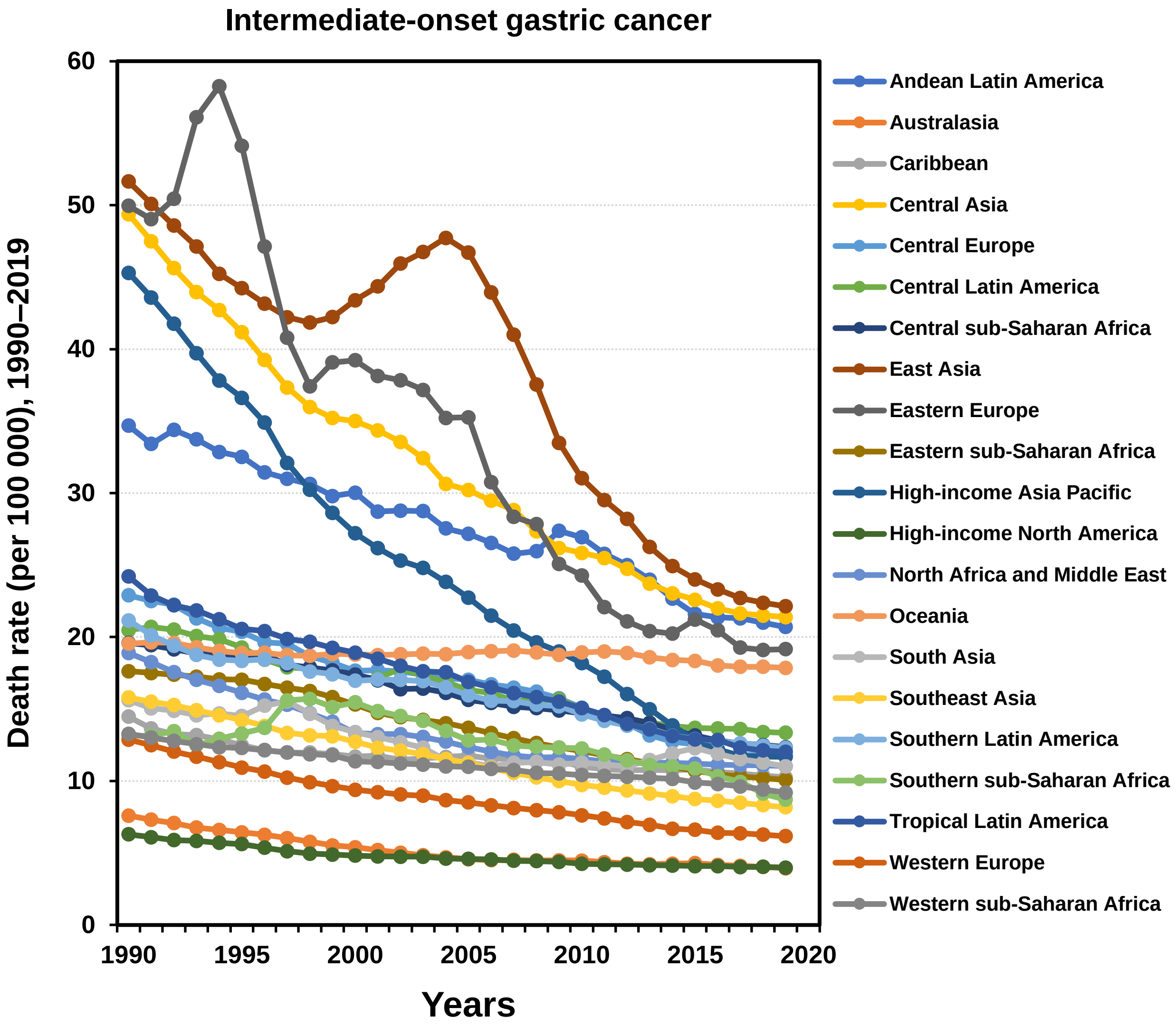
Intermediate-onset gastric cancer
Death rate (per 100 000), 1990–2019
Years
0
10
20
30
40
50
60
1990
1995
2000
2005
2010
2015
2020
Andean Latin America
Australasia
Caribbean
Central Asia
Central Europe
Central Latin America
Central sub-Saharan Africa
East Asia
Eastern Europe
Eastern sub-Saharan Africa
High-income Asia Pacific
High-income North America
North Africa and Middle East
Oceania
South Asia
Southeast Asia
Southern Latin America
Southern sub-Saharan Africa
Tropical Latin America
Western Europe
Western sub-Saharan Africa

Figure S15

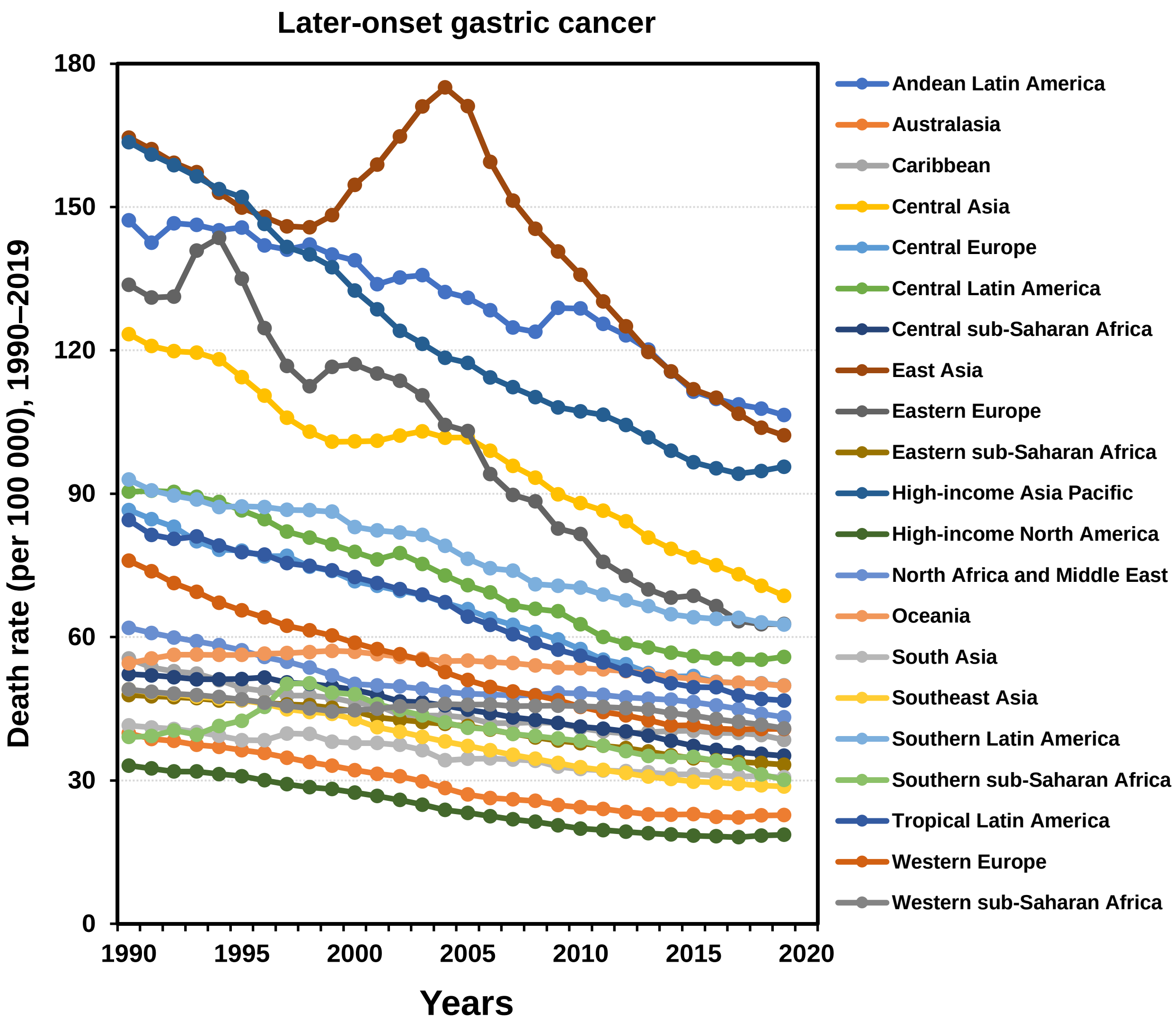
Later-onset gastric cancer
Death rate (per 100 000), 1990–2019
0
30
60
90
120
150
180
1990
1995
2000
2005
2010
2015
2020
Years
Andean Latin America
Australasia
Caribbean
Central Asia
Central Europe
Central Latin America
Central sub-Saharan Africa
East Asia
Eastern Europe
Eastern sub-Saharan Africa
High-income Asia Pacific
High-income North America
North Africa and Middle East
Oceania
South Asia
Southeast Asia
Southern Latin America
Southern sub-Saharan Africa
Tropical Latin America
Western Europe
Western sub-Saharan Africa

Figure S16

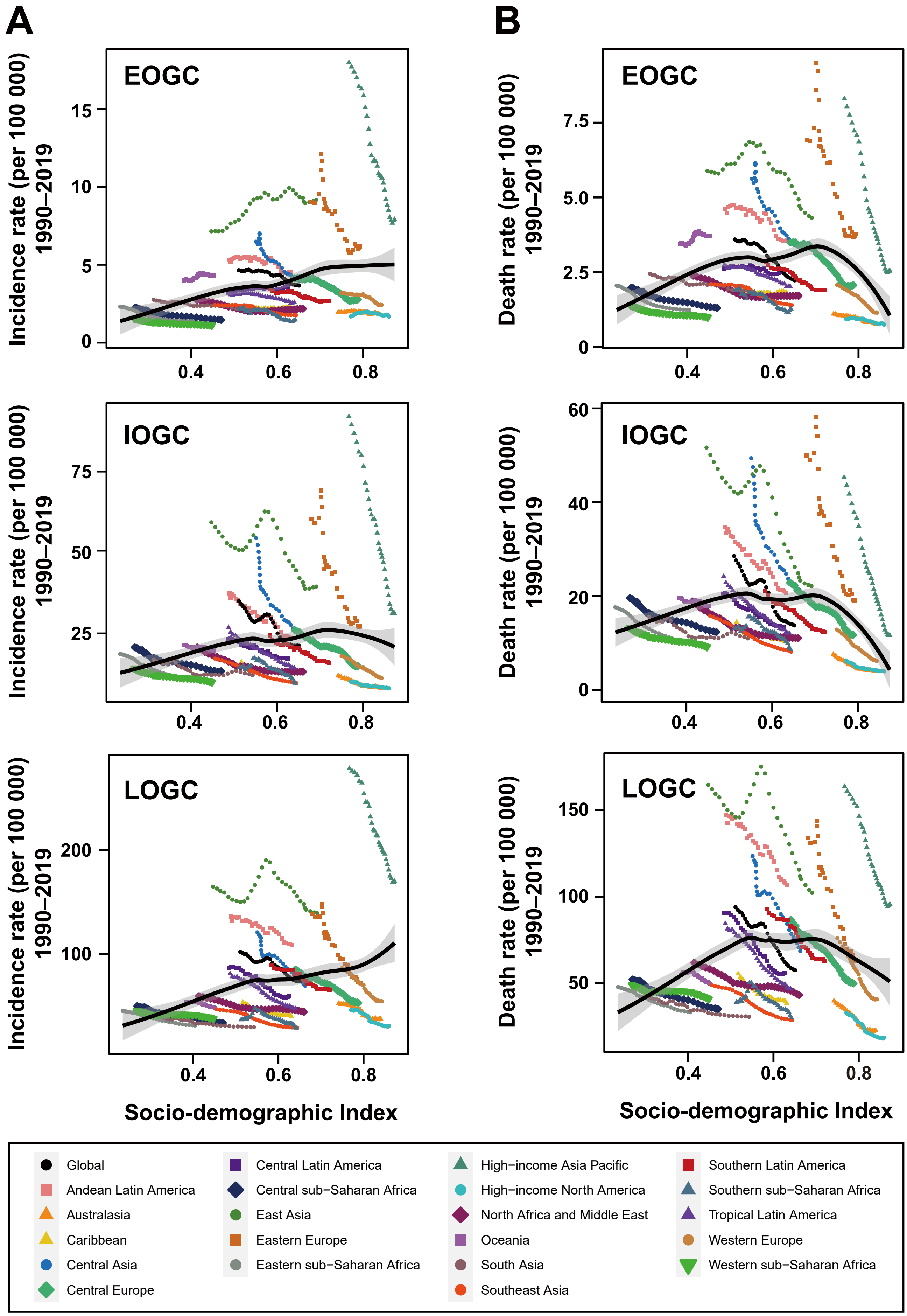
A
B
EOGC
IOGC
LOGC
Incidence rate (per 100 000) 1990–2019
Death rate (per 100 000) 1990–2019
15
10
5
0
7.5
5.0
2.5
75
50
25
60
40
20
200
100
150
0.4
0.6
0.8
Socio-demographic Index
Global
Andean Latin America
Australasia
Caribbean
Central Asia
Central Europe
Central Latin America
Central sub−Saharan Africa
East Asia
Eastern Europe
Eastern sub−Saharan Africa
High−income Asia Pacific
High−income North America
North Africa and Middle East
Oceania
South Asia
Southeast Asia
Southern Latin America
Southern sub−Saharan Africa
Tropical Latin America
Western Europe
Western sub−Saharan Africa

Figure S17

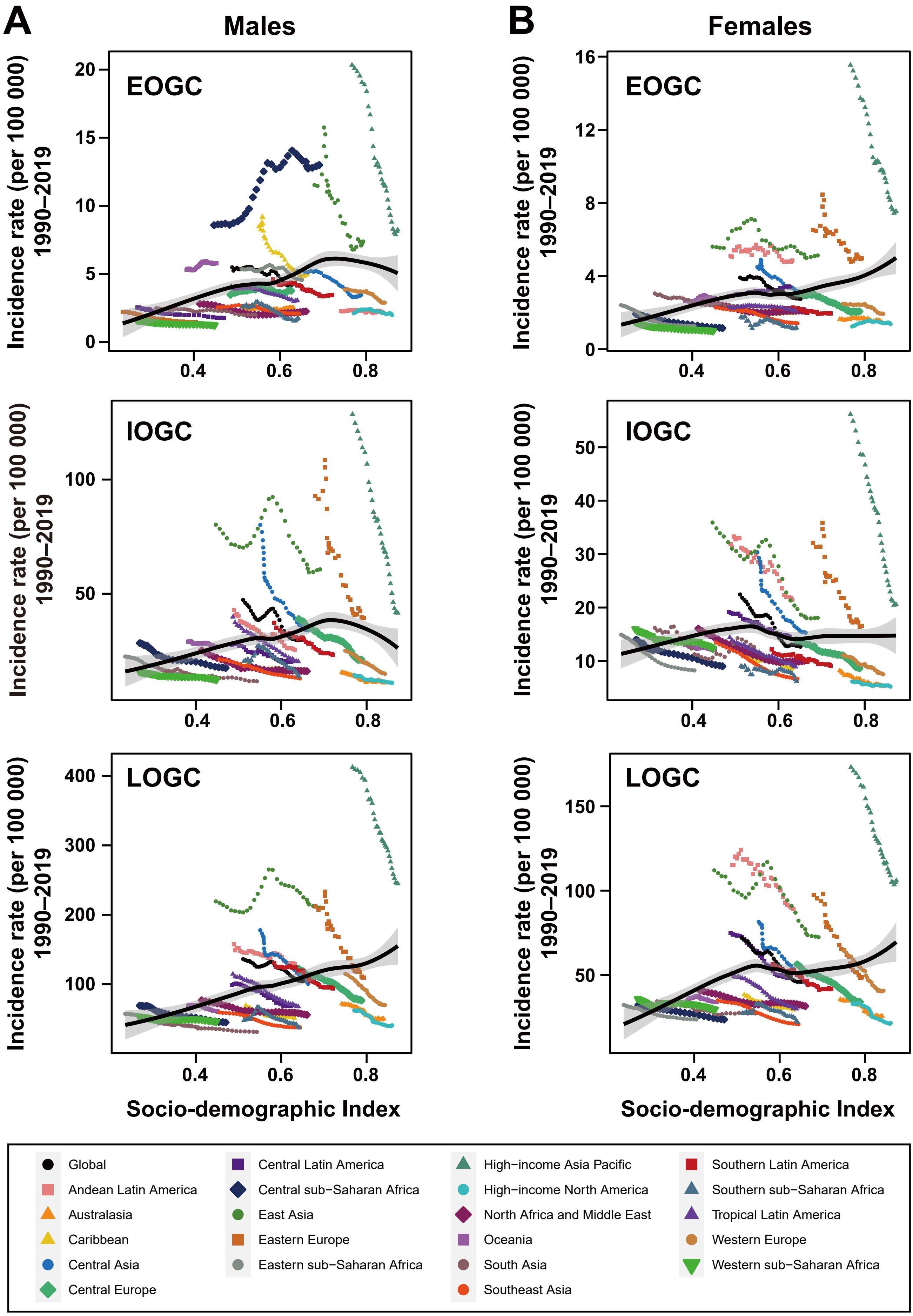

A
Males
B
Females
EOGC
IOGC
LOGC
Incidence rate (per 100 000) 1990–2019
Socio-demographic Index
0.4
0.6
0.8
0
5
10
15
20
0
4
8
12
16
50
100
10
20
30
40
50
100
200
300
400
50
100
150
Global
Andean Latin America
Australasia
Caribbean
Central Asia
Central Europe
Central Latin America
Central sub−Saharan Africa
East Asia
Eastern Europe
Eastern sub−Saharan Africa
High−income Asia Pacific
High−income North America
North Africa and Middle East
Oceania
South Asia
Southeast Asia
Southern Latin America
Southern sub−Saharan Africa
Tropical Latin America
Western Europe
Western sub−Saharan Africa

Figure S18

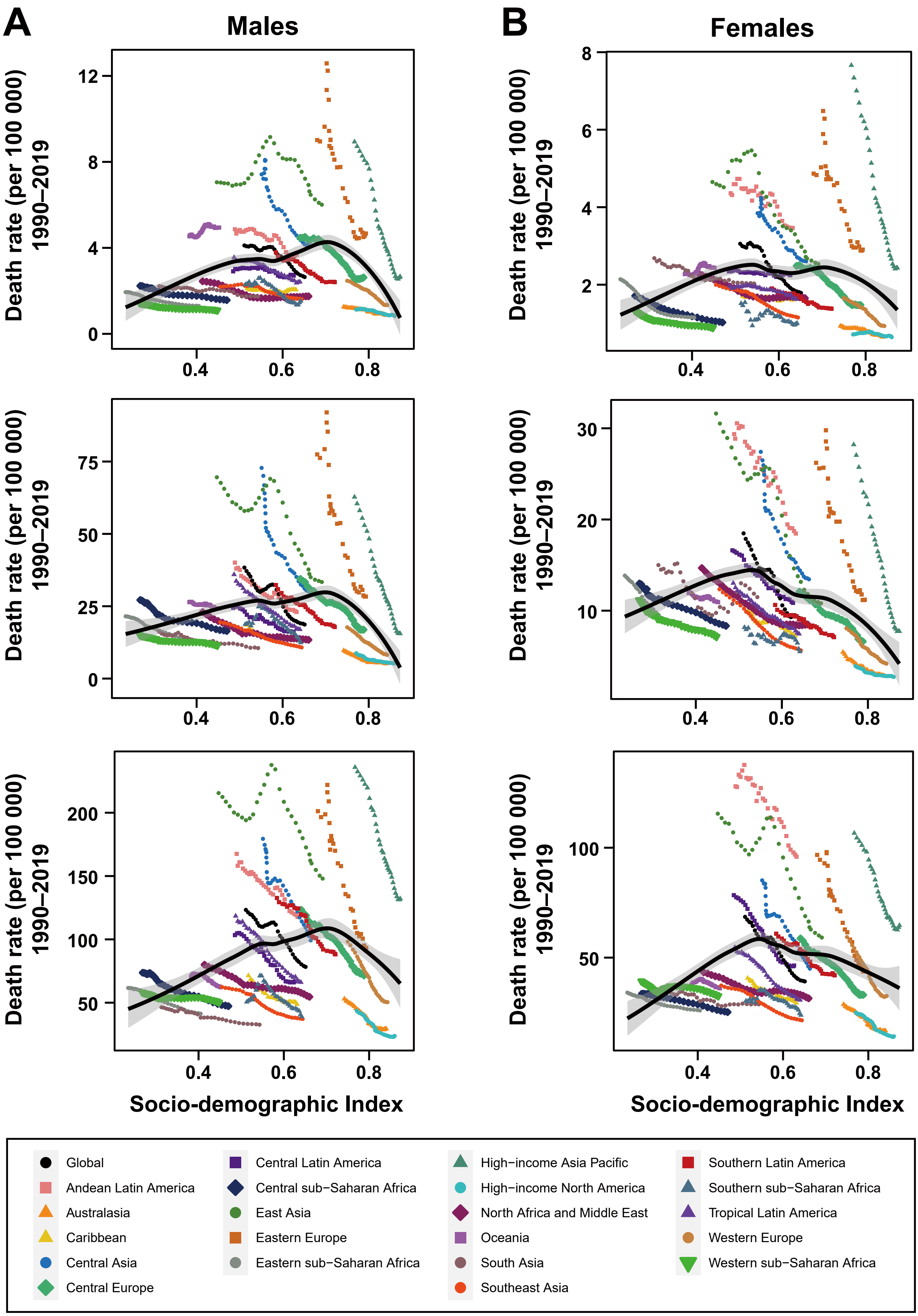
A
Males
B
Females
Death rate (per 100 000) 1990–2019
Socio-demographic Index
Global
Andean Latin America
Australasia
Caribbean
Central Asia
Central Europe
Central Latin America
Central sub−Saharan Africa
East Asia
Eastern Europe
Eastern sub−Saharan Africa
High−income Asia Pacific
High−income North America
North Africa and Middle East
Oceania
South Asia
Southeast Asia
Southern Latin America
Southern sub−Saharan Africa
Tropical Latin America
Western Europe
Western sub−Saharan Africa

Figure S19

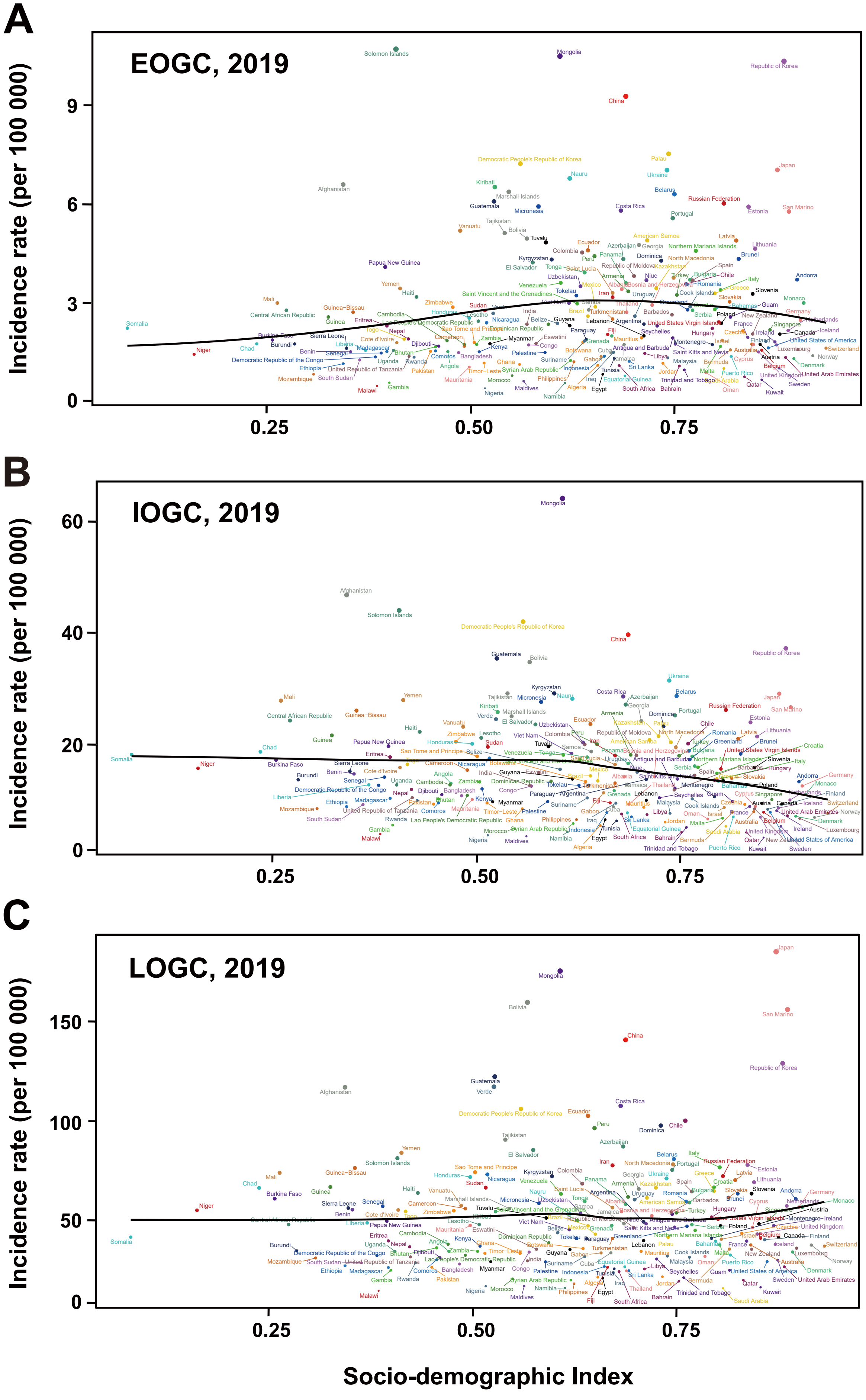
A
EOGC, 2019
Incidence rate (per 100 000)
0
3
6
9
0.25
0.50
0.75
B
IOGC, 2019
Incidence rate (per 100 000)
0
20
40
60
0.25
0.50
0.75
C
LOGC, 2019
Incidence rate (per 100 000)
0
50
100
150
0.25
0.50
0.75
Socio-demographic Index

Figure S20

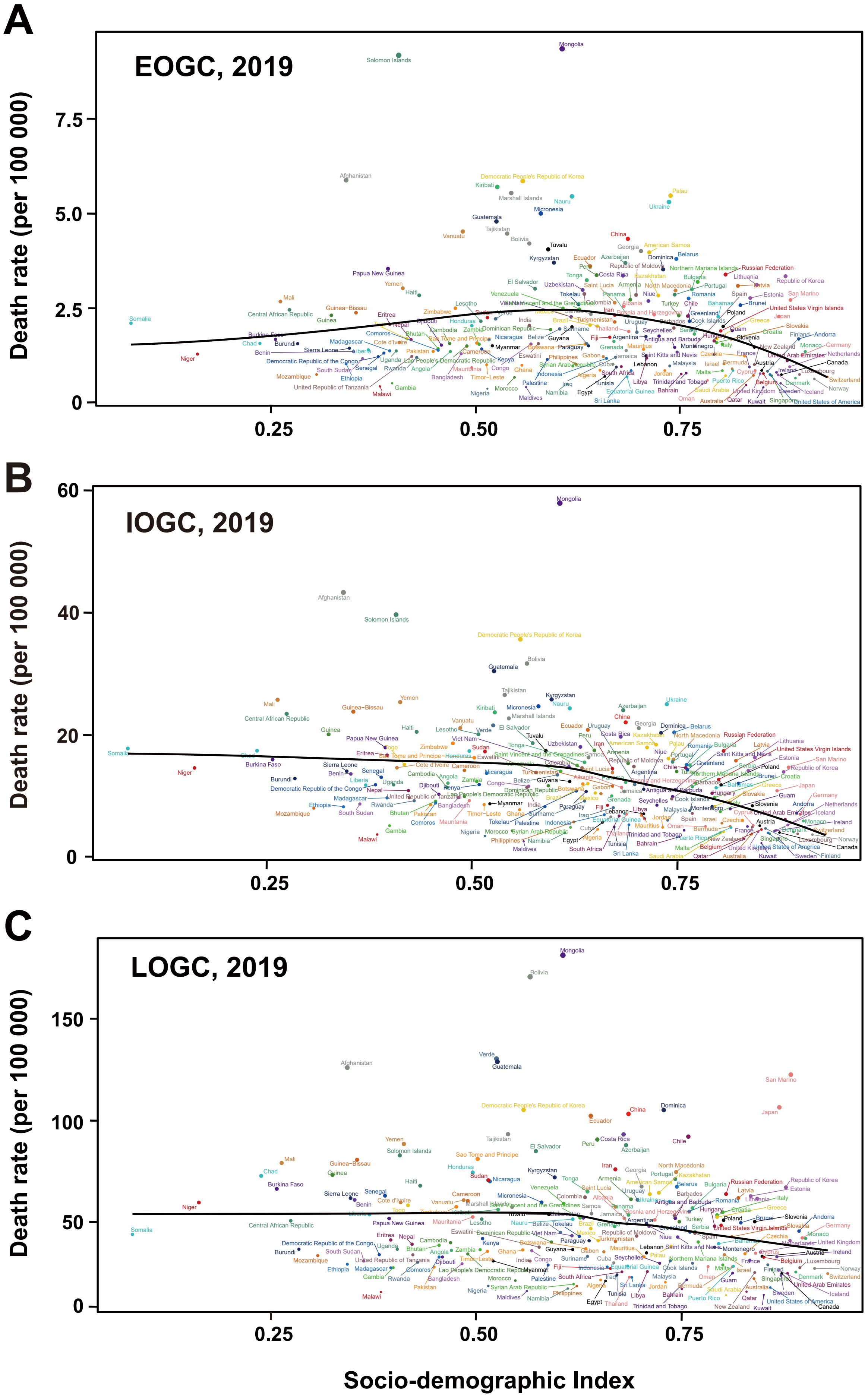
A
EOGC, 2019
Death rate (per 100 000)
0
2.5
5.0
7.5
0.25
0.50
0.75
B
IOGC, 2019
Death rate (per 100 000)
0
20
40
60
0.25
0.50
0.75
C
LOGC, 2019
Death rate (per 100 000)
0
50
100
150
0.25
0.50
0.75
Socio-demographic Index

Figure S21
