# Supplementary material for: Global, Regional, and National Burdens with Temporal Trends of Early-, Intermediate-, and Later-Onset Gastric Cancer from 1990 to 2019 and Predictions up to 2035
Source: Cancers (Basel). 2022 Nov 3;14(21):5417. doi: 10.3390/cancers14215417 (PMC9656932; doi:10.3390/cancers14215417)
Supplement: Supplementary file 1 [file cancers-14-05417-s001.zip › Supplementary Methods.pdf]

Supplementary Methods

Data Sources

The numbers of cases, numbers of deaths, and the corresponding rates were estimated for early-onset gastric cancer (EOGC), intermediate-onset gastric cancer (IOGC), and later-onset gastric cancer (LOGC) in relation to sex, region, and country based on from the Global Burden of Disease (GBD) Study 2019. Geographically, the world was divided into five sociodemographic index (SDI) and 21 GBD regions. Cancer incidence and death data were obtained from either a single cancer registry or from an aggregate database of cancer registries. However, not every country has a tumor registry system covering the entire population, and tumor registration may only cover a few areas in developing countries and may be absent in some underdeveloped areas.

Estimations of Incidence, Death, and Corresponding Rates

The numbers of cases and deaths due to EOGC, IOGC, and LOGC and their corresponding rates were estimated from GBD 2019. Incidence and death rates were reported per 100 000 person-years with 95% uncertainty intervals. Details of the statistical methods have been described previously [1,2].

Estimated Annual Percentage Change (EAPC)

The EAPC was calculated to quantify the time trends in incidence and death rates from 1990 to 2019 using the formula: $\ln(y) = \alpha + \beta x + \varepsilon$, where y was the incidence rate or death rate and x was the calendar year [3,4]. EAPC with 95% confidence intervals (95% CI) was calculated as $100 \times (\exp(\beta) - 1)$ for the time trends in incidence and death rates [5,6]. The incidence or death rate was defined as showing an increasing trend if both the EAPC and 95%CI were > 0, a decreasing trend if both were < 0, and stable if the 95%CI of EAPC included 0 [5]. The EAPC was analyzed using the Joinpoint Regression Program (version 4.8.0.1; Statistical Research and Applications Branch, National Cancer Institute) and R version 4.1.2 (R Foundation for Statistical Computing, Vienna,

Austria).

SDI

SDI is a comprehensive measure of educational level, income per capita, and fertility rate, and the SDI values range from 0 to 1 [7]. The expected relationship between SDI and incidence rate or death rate was analyzed by fitting a Gaussian process regression from 1990 to 2019. We further analyzed the associations between EAPC and SDI in 2019 by Pearson's correlation analyses. All countries were grouped into high-, high-middle-, middle-, low-middle-, and low-SDI regions based on the SDI values in 2019 [8].

Predictions for EOGC, IOGC, and LOGC up to 2035

Increasing evidence has shown that the Bayesian age-period-cohort (BAPC) model has higher accuracy for predicting cancer burden, especially in non-longer projection years, than generalized additive, smooth spline, Nordpred, Joinpoint, and Poisson regression models, and details of the BAPC model have been described elsewhere [9-11]. Therefore, the BAPC model within the integrated nested Laplacian approximation (R packages BAPC and INLA) was applied to predict the burden of EOGC, IOGC and LOGC from 2020 to 2035. The predicted-period incidence rates per 100,000 for the three age groups were calculated by the predicted incidence cases and populations of estimation.

References

1. Ning, F.L.; Lyu, J.; Pei, J.P.; Gu, W.J.; Zhang, N.N.; Cao, S.Y.; Zeng, Y.J.; Abe, M.; Nishiyama, K.; Zhang, C.D. The burden and trend of gastric cancer and possible risk factors in five Asian countries from 1990 to 2019. *Sci Rep* **2022**, *12*, 5980.

2. Gu, W.J.; Ning, F.L.; Jin, H.R.; Zhao, Z.M.; Lyu, J.; Pei, J.P.; Cao, S.Y.; Zeng, Y.J.; Abe, M.; Nishiyama, K.; Zhang, C.D. Burden and trends of inflammatory bowel disease in five Asian countries from 1990 to 2019: A comparison with the United States and the United Kingdom. *Dis Colon Rectum* **2022**.

3. Gao, S.; Yang, W.S.; Bray, F.; Va, P.; Zhang, W.; Gao, J.; Xiang, Y.B. Declining rates of hepatocellular carcinoma in urban Shanghai: incidence trends in 1976-2005. *Eur J Epidemiol* **2012**, *27*, 39-46.

4. Deng, Y.; Zhao, P.; Zhou, L.; Xiang, D.; Hu, J.; Liu, Y.; Ruan, J.; Ye, X.; Zheng, Y.; Yao, J.; et al. Epidemiological trends of tracheal, bronchus, and lung cancer at the global, regional, and national levels: a population-based study. *J Hematol Oncol* **2020**, *13*, 98.

5. Hankey, B.F.; Ries, L.A.; Kosary, C.L.; Feuer, E.J.; Merrill, R.M.; Clegg, L.X.; Edwards, B.K. Partitioning linear trends in age-adjusted rates. *Cancer Causes Control* **2000**, *11*, 31-35.

6. Yang, X.; Zhang, T.; Zhang, Y.; Chen, H.; Sang, S. Global burden of COPD attributable to ambient PM2.5 in 204 countries and territories, 1990 to 2019: A systematic analysis for the Global Burden of Disease Study 2019. *Sci Total Environ* **2021**, *796*, 148819.

7. GBD 2017 Population and Fertility Collaborators. Population and fertility by age and sex for 195 countries and territories, 1950-2017: a systematic analysis for the Global Burden of Disease Study 2017. *Lancet* **2018**, *392*, 1995-2051.

8. GBD 2016 Causes of Death Collaborators. Global, regional, and national age-

sex specific mortality for 264 causes of death, 1980-2016: a systematic analysis for the Global Burden of Disease Study 2016. *Lancet* **2017**, *390*, 1151-1210.

9. Yu, J.; Yang, X.; He, W.; Ye, W. Burden of pancreatic cancer along with attributable risk factors in Europe between 1990 and 2019, and projections until 2039. *Int J Cancer* **2021**, *149*, 993-1001.
10. Knoll, M.; Furkel, J.; Debus, J.; Abdollahi, A.; Karch, A.; Stock, C. An R package for an integrated evaluation of statistical approaches to cancer incidence projection. *BMC Med Res Methodol* **2020**, *20*, 257.
11. Du, Z.; Chen, W.; Xia, Q.; Shi, O.; Chen, Q. Trends and projections of kidney cancer incidence at the global and national levels, 1990-2030: a Bayesian age-period-cohort modeling study. *Biomark Res* **2020**, *8*, 16.
