# Supplementary material for: Global, Regional, and National Burdens with Temporal Trends of Early-, Intermediate-, and Later-Onset Gastric Cancer from 1990 to 2019 and Predictions up to 2035
Source: Cancers (Basel). 2022 Nov 3;14(21):5417. doi: 10.3390/cancers14215417 (PMC9656932; doi:10.3390/cancers14215417)
Supplement: Supplementary file 1 [file cancers-14-05417-s001.zip › Supplementary Tables.pdf]

Table S1. Death and temporal trends globally and by sex, and SDI and GBD regions, 1990–2019

Population	1990		2019		1990–2019
	**Death cases No. (95% UI)**	**DR (per 100 000) No. (95% UI)**	**Death cases No. (95% UI)**	**DR (per 100 000) No. (95% UI)**	**EAPC No. (95% CI)**
Global					
EOGC	97383 (90808–104062)	3.6 (3.3–3.8)	87333 (79904–95000)	2.2 (2.0–2.4)	–1.78* (–2.00 to –1.57)
IOGC	60688 (56418–65167)	28.5 (26.5–30.7)	60169 (53940–66427)	13.8 (12.3–15.2)	–2.55* (–2.85 to –2.26)
LOGC	630247 (593255–666176)	93.9 (88.4–99.3)	809683 (737694–873985)	57.6 (52.5–62.2)	–1.69* (–1.88 to –1.51)
Sex					
Male					
EOGC	56507 (51656–61283)	4.1 (3.8–4.5)	52594 (46921–58651)	2.6 (2.4–2.9)	–1.52* (–1.79 to –1.25)
IOGC	41296 (37455–45341)	38.4 (34.8–42.1)	40658 (35094–46597)	18.7 (16.1–21.4)	–2.45* (–2.79 to –2.11)
LOGC	383498 (353987–414008)	123.3 (113.8–133.1)	518252 (461036–573859)	78.3 (69.6–86.7)	–1.53* (–1.72 to –1.34)
Female					
EOGC	40875 (36852–44764)	3.1 (2.8–3.3)	34739 (31191–38545)	1.8 (1.6–2.0)	–2.17* (–2.37 to –1.96)
IOGC	19391 (17519–21193)	18.5 (16.7–20.2)	19511 (17301–22194)	8.9 (7.9–10.1)	–2.70* (–2.92 to –2.47)
LOGC	246749 (228756–264041)	68.5 (63.5–73.3)	291431 (257354–322447)	39.2 (34.6–43.3)	–2.01* (–2.19 to –1.84)
Sociodemographic index (SDI)					
High SDI					
EOGC	12811 (12569–13027)	3.0 (2.9–3.0)	5718 (5463–5995)	1.2 (1.2–1.3)	–3.31* (–3.40 to –3.22)
IOGC	8102 (7868–8319)	18.6 (18.0–19.1)	4391 (4154–4638)	6.4 (6.0–6.7)	–3.81* (–3.94 to –3.68)
LOGC	133745 (127517–136918)	76.2 (72.6–78.0)	134681 (118723–143813)	43.2 (38.1–46.2)	–2.16* (–2.23 to –2.10)
High-middle SDI					
EOGC	27950 (25910–30134)	4.6 (4.3–5.0)	20716 (18484–22954)	2.8 (2.5–3.2)	–2.05* (–2.34 to –1.76)
IOGC	21072 (19652–22629)	37.3 (34.8–40.1)	16048 (13911–18418)	15.7 (13.6–18.1)	–3.04* (–3.41 to –2.66)
LOGC	227416 (213982–240343)	122.8 (115.6–129.8)	244191 (219174–267421)	68.8 (61.8–75.4)	–2.05* (–2.28 to –1.81)
Middle SDI					
EOGC	34946 (31454–38871)	3.9 (3.5–4.3)	31097 (27804–34950)	2.5 (2.2–2.8)	–1.59* (–1.90 to –1.27)
IOGC	20400 (17893–23043)	33.5 (29.4–37.9)	23807 (20501–27237)	15.5 (13.3–17.7)	–2.71* (–3.15 to –2.27)
LOGC	189007 (171461–208183)	109.8 (99.6–120.9)	290366 (256668–325521)	67.7 (59.9–75.9)	–1.54* (–1.85 to –1.23)
Low-middle SDI					
EOGC	16459 (14723–17964)	3.0 (2.7–3.3)	21409 (19236–23772)	2.3 (2.1–2.5)	–0.95* (–1.06 to –0.83)
IOGC	8506 (7606–9414)	23.1 (20.7–25.6)	11929 (10502–13499)	15.0 (13.2–16.9)	–1.56* (–1.66 to –1.45)
LOGC	60513 (55414–65148)	61.1 (56.0–65.8)	108619 (99648–118189)	47.9 (43.8–52.0)	–0.82* (–0.94 to –0.71)
Low SDI					
EOGC	5186 (4411–5845)	2.2 (1.9–2.5)	8351 (7187–9592)	1.5 (1.3–1.8)	–1.29* (–1.31 to –1.26)
IOGC	2592 (2237–2927)	17.5 (15.1–19.8)	3971 (3370–4593)	12.2 (10.4–14.1)	–1.40* (–1.49 to –1.31)
LOGC	19366 (17266–21320)	49.9 (44.5–54.9)	31554 (28649–34779)	38.0 (34.5–41.9)	–0.95* (–1.01 to –0.90)
GBD regions					
Andean Latin America					
EOGC	857 (773–951)	4.6 (4.2–5.1)	1185 (925–1504)	3.6 (2.8–4.5)	–1.00* (–1.21 to –0.79)
IOGC	399 (346–456)	34.7 (30.1–39.6)	620 (466–815)	20.7 (15.5–27.2)	–1.75* (–1.88 to –1.61)
LOGC	4946 (4492–5400)	147.2 (133.7–160.7)	9985 (8196–11945)	106.4 (87.4–127.3)	–1.11* (–1.23 to –0.99)
Australasia					
EOGC	118 (112–125)	1.1 (1.0–1.2)	102 (90–115)	0.76 (0.67–0.85)	–1.23* (–1.32 to –1.14)
IOGC	73 (67–80)	7.6 (7.0–8.3)	72 (60–85)	3.9 (3.3–4.7)	–2.16* (–2.39 to –1.92)
LOGC	1570 (1488–1632)	39.9 (37.8–41.4)	1872 (1668–2046)	22.7 (20.3–24.8)	–2.23* (–2.37 to –2.08)
Caribbean					
EOGC	347 (293–386)	1.9 (1.6–2.1)	437 (349–529)	1.8 (1.5–2.2)	–0.14* (–0.22 to –0.05)
IOGC	188 (163–216)	14.5 (12.5–16.6)	280 (224–344)	10.3 (8.2–12.6)	–0.90* (–1.02 to –0.79)
LOGC	2398 (2201–2535)	55.5 (51.0–58.7)	3401 (2950–3880)	38.5 (33.4–43.9)	–1.16* (–1.27 to –1.05)
Central Asia					
EOGC	1875 (1799–1948)	5.6 (5.4–5.8)	1680 (1498–1900)	3.4 (3.1–3.9)	–2.26* (–2.49 to –2.04)
IOGC	1468 (1396–1539)	49.4 (46.9–51.8)	1023 (889–1184)	21.4 (18.6–24.8)	–2.91* (–3.00 to –2.81)
LOGC	9840 (9481–10151)	123.4 (118.9–127.3)	8938 (8122–9849)	68.7 (62.4–75.7)	–1.88* (–2.03 to –1.73)
Central Europe					
EOGC	2128 (2077–2181)	3.5 (3.4–3.6)	1086 (933–1254)	2.1 (1.8–2.4)	–2.35* (–2.56 to –2.15)
IOGC	1576 (1515–1637)	22.9 (22.0–23.8)	879 (748–1022)	11.8 (10.0–13.7)	–2.30* (–2.44 to –2.16)
LOGC	22626 (21946–23080)	86.6 (84.0–88.3)	18147 (16005–20391)	49.9 (44.0–56.0)	–2.01* (–2.10 to –1.93)
Central Latin America					
EOGC	2137 (2073–2198)	2.6 (2.5–2.7)	3253 (2730–3877)	2.5 (2.1–2.9)	–0.37* (–0.46 to –0.28)

Population	1990		2019		1990–2019
	Death cases No. (95% UI)	DR (per 100 000) No. (95% UI)	Death cases No. (95% UI)	DR (per 100 000) No. (95% UI)	EAPC No. (95% CI)
IOGC	995 (953–1036)	20.5 (19.6–21.3)	1730 (1439–2083)	13.4 (11.1–16.1)	–1.59* (–1.68 to –1.50)
LOGC	12316 (11716–12704)	90.4 (86.0–93.3)	22318 (19154–25902)	55.8 (47.9–64.8)	–1.98* (–2.08 to –1.88)
Central sub-Saharan Africa					
EOGC	480 (369–599)	2.0 (1.5–2.5)	805 (599–1067)	1.3 (1.0–1.7)	–1.51* (–1.59 to –1.43)
IOGC	290 (215–376)	19.6 (14.6–25.4)	466 (330–635)	12.4 (8.7–16.8)	–1.64* (–1.70 to –1.57)
LOGC	1984 (1664–2345)	52.3 (43.8–61.8)	3009 (2457–3721)	35.2 (28.7–43.5)	–1.46* (–1.57 to –1.36)
East Asia					
EOGC	40658 (34889–46566)	5.9 (5.1–6.7)	32211 (26787–38306)	4.3 (3.6–5.1)	–1.08* (–1.50 to –0.65)
IOGC	25759 (21769–29930)	51.6 (43.6–60.0)	28548 (23218–34603)	22.1 (18.0–26.8)	–2.90* (–3.43 to –2.37)
LOGC	246687 (216549–278021)	164.5 (144.4–185.4)	372232 (314718–432401)	102.2 (86.4–118.7)	–1.46* (–1.88 to –1.04)
Eastern Europe					
EOGC	7651 (7151–7923)	6.9 (6.5–7.2)	3707 (3263–4180)	3.8 (3.3–4.3)	–3.26* (–3.74 to –2.78)
IOGC	7920 (7500–8217)	50.0 (47.3–51.8)	2481 (2164–2843)	19.2 (16.7–22.0)	–4.03* (–4.38 to –3.68)
LOGC	65360 (63506–66674)	133.7 (129.9–136.4)	38820 (34993–42867)	62.7 (56.5–69.3)	–3.03* (–3.27 to –2.79)
Eastern sub-Saharan Africa					
EOGC	1695 (1350–1954)	2.0 (1.6–2.4)	2433 (2017–2917)	1.2 (1.0–1.5)	–2.05* (–2.17 to –1.94)
IOGC	820 (692–956)	17.6 (14.9–20.6)	1063 (886–1262)	10.1 (8.4–12.0)	–2.13* (–2.21 to –2.04)
LOGC	5821 (5074–6471)	47.9 (41.7–53.2)	8586 (7647–9638)	33.3 (29.7–37.4)	–1.37* (–1.45 to –1.30)
High-income Asia Pacific					
EOGC	7716 (7482–7894)	8.3 (8.1–8.5)	2069 (1943–2212)	2.5 (2.4–2.7)	–4.45* (–4.62 to –4.28)
IOGC	4708 (4516–4904)	45.3 (43.5–47.2)	1586 (1447–1732)	11.7 (10.7–12.8)	–4.75* (–4.88 to –4.62)
LOGC	57223 (54595–58651)	163.6 (156.1–167.6)	66201 (56159–71871)	95.6 (81.1–103.8)	–2.05* (–2.14 to –1.95)
High-income North America					
EOGC	1370 (1335–1401)	0.92 (0.90–0.94)	1226 (1174–1283)	0.74 (0.70–0.77)	–0.92* (–1.07 to –0.76)
IOGC	803 (778–825)	6.3 (6.1–6.5)	929 (880–983)	4.0 (3.8–4.2)	–1.50* (–1.64 to –1.35)
LOGC	19233 (18149–19819)	33.1 (31.2–34.1)	20161 (18539–21112)	18.6 (17.1–19.5)	–2.37* (–2.51 to –2.22)
North Africa and Middle East					
EOGC	3859 (3343–4341)	2.4 (2.1–2.7)	5676 (4740–6695)	1.7 (1.4–2.0)	–1.21* (–1.49 to –0.92)
IOGC	1933 (1637–2198)	18.9 (16.0–21.5)	3076 (2628–3587)	11.0 (9.4–12.9)	–1.85* (–2.07 to –1.63)
LOGC	17753 (15296–19420)	61.9 (53.4–67.7)	30853 (27934–33829)	43.2 (39.2–47.4)	–1.09* (–1.22 to –0.95)
Oceania					
EOGC	109 (82–136)	3.4 (2.6–4.3)	252 (183–338)	3.7 (2.7–5.0)	0.41* (0.30 to 0.51)
IOGC	38 (27–51)	19.5 (14.0–26.6)	91 (62–126)	17.9 (12.2–24.9)	–0.22* (–0.28 to –0.16)
LOGC	264 (210–321)	54.5 (43.3–64.4)	565 (442–702)	49.8 (38.9–61.8)	–0.42* (–0.51 to –0.33)
South Asia					
EOGC	12915 (11397–14185)	2.4 (2.2–2.7)	18314 (15928–21109)	1.9 (1.6–2.2)	–0.88* (–0.96 to –0.79)
IOGC	5630 (4934–6271)	15.6 (13.7–17.4)	8746 (7243–10286)	11.0 (9.1–12.9)	–1.24* (–1.51 to –0.96)
LOGC	38703 (34548–42778)	41.5 (37.0–45.9)	72002 (63034–81814)	30.7 (26.8–34.8)	–1.15* (–1.23 to –1.06)
Southeast Asia					
EOGC	4948 (4220–5517)	2.1 (1.8–2.3)	5020 (4324–5813)	1.4 (1.2–1.6)	–1.63* (–1.80 to –1.46)
IOGC	2501 (2167–2833)	15.8 (13.7–17.9)	3128 (2644–3672)	8.2 (6.9–9.6)	–2.35* (–2.40 to –2.30)
LOGC	20757 (18054–22856)	49.0 (42.6–54.0)	30060 (27062–33180)	28.7 (25.9–31.7)	–2.08* (–2.17 to –1.99)
Southern Latin America					
EOGC	695 (667–727)	2.8 (2.7–3.0)	643 (591–700)	1.9 (1.7–2.1)	–1.44* (–1.53 to –1.36)
IOGC	466 (434–501)	21.2 (19.7–22.8)	451 (396–509)	12.3 (10.8–13.9)	–1.73* (–1.82 to –1.64)
LOGC	7360 (7075–7599)	92.9 (89.4–96.0)	8860 (8231–9424)	62.6 (58.2–66.6)	–1.46* (–1.55 to –1.37)
Southern sub-Saharan Africa					
EOGC	492 (451–533)	1.9 (1.7–2.0)	535 (455–630)	1.3 (1.1–1.5)	–1.76* (–2.04 to –1.48)
IOGC	201 (178–226)	13.2 (11.7–14.8)	281 (235–332)	8.7 (7.3–10.3)	–1.32* (–1.73 to –0.90)
LOGC	1759 (1580–1927)	39.1 (35.1–42.8)	2841 (2632–3081)	30.1 (27.9–32.7)	–1.08* (–1.48 to –0.68)
Tropical Latin America					
EOGC	2154 (2078–2231)	2.7 (2.6–2.8)	2409 (2288–2526)	2.0 (1.9–2.1)	–1.07* (–1.20 to –0.94)
IOGC	1279 (1215–1342)	24.2 (23.0–25.4)	1561 (1460–1668)	12.0 (11.2–12.8)	–2.33* (–2.40 to –2.26)
LOGC	12770 (12093–13216)	84.5 (80.0–87.4)	19480 (17933–20496)	46.7 (43.0–49.2)	–2.16* (–2.27 to –2.05)
Western Europe					
EOGC	4002 (3925–4072)	2.1 (2.0–2.1)	2169 (2043–2296)	1.1 (1.1–1.2)	–2.12* (–2.24 to –2.01)
IOGC	2950 (2859–3049)	12.9 (12.5–13.3)	1987 (1838–2137)	6.2 (5.7–6.6)	–2.48* (–2.55 to –2.41)
LOGC	73830 (70270–75760)	76.0 (72.3–77.9)	58969 (52935–62602)	40.8 (36.6–43.3)	–2.27* (–2.37 to –2.17)

Population	1990		2019		1990–2019
	**Death cases No. (95% UI)**	**DR (per 100 000) No. (95% UI)**	**Death cases No. (95% UI)**	**DR (per 100 000) No. (95% UI)**	**EAPC No. (95% CI)**
Western sub-Saharan Africa					
EOGC	1176 (1000–1360)	1.4 (1.2–1.6)	2120 (1707–2570)	0.99 (0.79–1.2)	–1.06* (–1.14 to –0.99)
IOGC	694 (579–821)	13.2 (11.1–15.7)	1170 (946–1439)	9.2 (7.4–11.3)	–1.11* (–1.16 to –1.07)
LOGC	7047 (6161–7878)	49.0 (42.9–54.8)	12373 (10849–13986)	40.9 (35.9–46.2)	–0.40* (–0.50 to –0.31)

CI, confidence interval; DR, death rate; EAPC, estimated annual percentage change; EOGC, early-onset gastric cancer (15–49 years); IOGC, intermediate-onset gastric cancer (50–54 years); LOGC, later-onset gastric cancer (≥ 55 years); UI, uncertainty interval. *Indicates statistical significance.

Table S2. Incidence of early-onset gastric cancer in 203 countries, 2019 versus 1990

Country	Early-onset gastric cancer (15–49 years)											
	1990						2019					
	Incidence number			Incidence rate (per 100 000)			Incidence number			Incidence rate (per 100 000)		
	Value	LUI	UUI	Value	LUI	UUI	Value	LUI	UUI	Value	LUI	UUI
Afghanistan	388.3	195.1	550.2	8.2	4.1	11.5	1198.8	664.4	1766.6	6.6	3.7	9.7
Albania	56.3	51.2	61.2	3.3	3.0	3.6	44.1	32.0	58.9	3.4	2.5	4.6
Algeria	172.3	132.9	218.2	1.5	1.1	1.9	246.9	186.6	322.1	1.1	0.8	1.4
American Samoa	1.2	1.0	1.4	4.8	3.9	5.9	1.3	1.0	1.9	4.9	3.6	6.7
Andorra	1.0	0.7	1.3	2.9	2.1	4.0	1.5	1.1	2.1	3.7	2.6	5.1
Angola	129.5	92.5	177.2	2.8	2.0	3.8	204.9	140.9	277.2	1.5	1.0	2.0
Antigua and Barbuda	0.8	0.7	0.9	2.6	2.3	3.0	0.9	0.8	1.1	1.9	1.6	2.4
Argentina	461.1	433.9	490.9	2.9	2.7	3.1	545.2	408.1	717.7	2.4	1.8	3.1
Armenia	100.9	93.7	108.5	5.9	5.5	6.4	49.4	39.9	60.4	3.3	2.7	4.1
Australia	164.6	153.4	177.9	1.8	1.7	2.0	201.1	146.1	269.8	1.7	1.3	2.3
Austria	171.8	158.8	185.7	4.3	3.9	4.6	70.2	52.6	89.5	1.7	1.3	2.2
Azerbaijan	243.5	220.8	269.0	6.6	6.0	7.3	240.6	190.4	298.6	4.3	3.4	5.4
Bahamas	4.2	3.7	4.8	3.0	2.6	3.3	5.8	4.5	7.4	2.9	2.2	3.6
Bahrain	3.9	3.2	4.7	1.3	1.1	1.6	10.0	7.6	12.9	1.1	0.8	1.4
Bangladesh	1145.0	827.5	1394.9	2.3	1.6	2.8	1279.6	916.0	1897.0	1.5	1.1	2.2
Barbados	4.1	3.6	4.6	3.0	2.7	3.4	3.7	2.8	4.8	2.6	2.0	3.4
Belarus	585.8	551.4	621.8	11.6	10.9	12.3	274.2	203.7	373.6	6.3	4.7	8.6
Belgium	105.9	96.5	115.8	2.1	1.9	2.3	78.2	56.6	105.4	1.6	1.1	2.1
Belize	1.3	1.2	1.5	1.6	1.4	1.8	5.3	4.4	6.4	2.4	2.0	2.8
Benin	42.9	34.2	53.2	2.1	1.7	2.7	88.0	59.8	123.7	1.5	1.0	2.1
Bermuda	0.7	0.6	0.9	2.2	1.9	2.5	0.4	0.3	0.6	1.6	1.2	2.0
Bhutan	6.2	3.7	8.8	2.1	1.2	2.9	7.2	4.5	11.0	1.7	1.0	2.5
Bolivia	223.2	169.8	275.5	7.5	5.7	9.2	302.1	194.4	423.1	4.9	3.2	6.9
Bosnia and Herzegovina	70.8	65.0	78.0	2.9	2.7	3.2	44.5	33.1	59.2	2.9	2.2	3.9
Botswana	10.8	7.2	15.9	1.8	1.2	2.6	23.8	14.5	36.2	1.8	1.1	2.7
Brazil	2455.7	2371.9	2541.3	3.2	3.1	3.3	2994.3	2843.1	3153.3	2.6	2.5	2.7
Brunei Darussalam	10.7	8.9	12.6	7.3	6.1	8.6	11.5	9.6	13.9	4.3	3.6	5.2
Bulgaria	212.0	194.3	230.3	5.1	4.7	5.5	112.0	84.9	148.1	3.7	2.8	4.9
Burkina Faso	90.3	68.1	118.5	2.4	1.8	3.1	191.4	138.1	251.3	1.9	1.3	2.5
Burundi	70.4	51.8	91.5	2.9	2.1	3.8	95.8	68.1	130.5	1.7	1.2	2.4
Cabo Verde	6.1	5.2	7.2	4.1	3.5	4.9	8.2	6.1	11.0	2.7	2.0	3.6
Cambodia	151.8	105.5	194.4	3.3	2.3	4.2	162.4	120.1	218.8	1.8	1.4	2.5
Cameroon	103.0	80.1	127.1	2.3	1.8	2.8	241.4	156.9	347.2	1.7	1.1	2.4
Canada	302.8	279.0	327.5	2.1	1.9	2.2	331.1	248.1	440.8	2.0	1.5	2.7
Central African Republic	45.3	34.1	57.0	3.6	2.7	4.6	71.0	48.1	101.7	2.8	1.9	4.0
Chad	53.6	42.2	66.4	2.2	1.7	2.7	119.8	84.2	163.0	1.8	1.2	2.4
Chile	328.5	307.6	353.8	4.6	4.3	5.0	328.1	244.1	433.2	3.6	2.7	4.7
China	47740.1	41319.2	54559.3	7.1	6.2	8.2	66758.8	54936.1	80133.0	9.3	7.6	11.1
Colombia	773.7	730.3	817.8	4.6	4.3	4.8	1126.9	817.5	1488.6	4.5	3.3	6.0
Comoros	3.6	1.0	5.6	1.8	0.5	2.7	5.7	3.4	7.9	1.5	0.9	2.1
Congo	32.6	23.6	43.2	2.9	2.1	3.9	46.0	30.4	66.1	1.7	1.1	2.5
Cook Islands	0.3	0.2	0.4	3.1	2.3	4.2	0.2	0.1	0.3	2.9	1.8	4.0
Costa Rica	96.9	89.7	104.7	6.3	5.8	6.8	144.6	108.0	190.5	5.8	4.3	7.6
Croatia	127.6	114.9	141.0	5.2	4.7	5.8	52.1	38.2	69.7	2.8	2.0	3.7
Cuba	95.4	88.1	103.3	1.6	1.4	1.7	78.3	60.2	99.4	1.5	1.1	1.9
Cyprus	4.9	4.1	5.8	1.2	1.0	1.4	11.9	9.7	14.6	1.7	1.4	2.1
Czechia	208.5	193.5	223.9	4.0	3.7	4.3	102.9	76.8	134.1	2.1	1.6	2.8
Cote d'Ivoire	135.2	101.6	172.2	2.4	1.8	3.1	233.4	158.9	322.2	1.8	1.2	2.5
Democratic People's Republic of Korea	1034.2	681.3	1460.5	9.7	6.4	13.7	1004.5	648.6	1505.7	7.2	4.7	10.8
Democratic Republic of the Congo	321.2	238.9	415.5	1.9	1.4	2.5	565.1	406.2	759.7	1.4	1.0	1.8
Denmark	74.0	67.4	81.9	2.8	2.5	3.1	33.4	24.6	45.6	1.3	1.0	1.8
Djibouti	3.8	2.6	5.5	1.6	1.1	2.3	10.8	6.2	17.3	1.7	1.0	2.7
Dominica	1.8	1.4	2.1	5.0	4.1	6.1	1.5	1.1	1.9	4.3	3.2	5.5
Dominican Republic	61.1	52.5	70.3	1.7	1.4	1.9	135.4	95.4	189.6	2.3	1.7	3.3
Ecuador	241.8	224.2	258.8	4.9	4.5	5.2	421.9	315.7	554.8	4.6	3.4	6.0
Egypt	287.7	251.8	329.8	1.1	0.9	1.2	426.4	304.1	580.7	0.8	0.6	1.1
El Salvador	90.9	83.6	98.0	3.6	3.3	3.9	138.5	100.8	190.1	4.2	3.1	5.8
Equatorial Guinea	5.4	3.7	7.3	3.0	2.1	4.0	5.8	3.4	9.7	0.8	0.5	1.3
Eritrea	38.5	28.0	49.5	2.8	2.1	3.6	79.4	53.1	113.7	2.3	1.5	3.3
Estonia	70.1	64.4	76.1	9.2	8.5	10.0	34.4	24.8	46.1	5.9	4.3	7.9
Eswatini	5.5	4.1	7.2	1.5	1.2	2.0	10.7	6.5	16.0	1.8	1.1	2.6
Ethiopia	831.0	500.9	1037.9	3.8	2.3	4.7	603.2	449.5	816.9	1.1	0.9	1.6

Country	Early-onset gastric cancer (15–49 years)											
	1990						2019					
	Incidence number			Incidence rate (per 100 000)			Incidence number			Incidence rate (per 100 000)		
	Value	LUI	UUI	Value	LUI	UUI	Value	LUI	UUI	Value	LUI	UUI
Fiji	9.1	7.2	11.5	2.3	1.8	2.9	9.5	6.9	12.6	2.0	1.5	2.7
Finland	107.7	97.0	117.8	4.2	3.8	4.6	42.7	31.3	56.7	1.8	1.3	2.4
France	601.4	558.8	646.8	2.1	1.9	2.2	554.2	406.9	744.6	1.9	1.4	2.6
Gabon	10.4	7.8	13.3	2.3	1.8	3.0	12.7	8.7	18.2	1.4	0.9	2.0
Gambia	2.9	2.0	4.1	0.7	0.5	0.9	6.4	4.4	8.8	0.6	0.4	0.8
Georgia	172.9	153.1	193.7	6.4	5.7	7.2	78.6	62.0	97.4	4.8	3.7	5.9
Germany	1349.8	1278.3	1425.5	3.4	3.2	3.6	886.8	644.6	1211.3	2.5	1.8	3.4
Ghana	125.3	93.6	162.8	1.8	1.4	2.4	187.2	128.6	254.4	1.1	0.8	1.5
Greece	178.7	164.2	193.9	3.5	3.3	3.8	146.8	108.3	198.0	3.3	2.4	4.4
Greenland	1.6	1.2	2.0	4.7	3.5	6.0	0.7	0.6	1.0	2.8	2.1	3.7
Grenada	1.1	1.0	1.3	2.8	2.4	3.3	1.1	0.9	1.3	2.0	1.6	2.5
Guam	1.4	1.1	1.6	1.8	1.5	2.1	2.1	1.7	2.6	2.6	2.0	3.2
Guatemala	226.9	196.9	262.1	6.5	5.7	7.6	581.4	440.7	744.7	6.1	4.6	7.8
Guinea	78.0	61.1	95.5	3.0	2.3	3.6	146.8	105.5	198.8	2.6	1.8	3.5
Guinea-Bissau	18.4	13.7	24.3	4.2	3.1	5.5	25.7	18.7	34.7	2.7	2.0	3.7
Guyana	10.0	8.2	12.1	2.5	2.0	3.0	9.6	6.9	13.0	2.3	1.7	3.1
Haiti	144.3	85.9	182.9	4.9	2.9	6.2	208.1	123.5	291.6	3.2	1.9	4.4
Honduras	79.1	61.3	95.3	3.9	3.0	4.7	131.0	78.5	201.1	2.5	1.5	3.9
Hungary	231.0	212.9	251.0	4.5	4.2	4.9	98.7	76.4	126.7	2.2	1.7	2.9
Iceland	4.0	3.4	4.6	3.0	2.6	3.4	2.8	2.3	3.4	1.7	1.4	2.1
India	12497.3	11029.6	13919.3	3.0	2.6	3.3	17827.1	15025.9	20927.1	2.3	2.0	2.8
Indonesia	1918.0	1473.4	2199.2	2.0	1.6	2.3	1961.8	1560.4	2493.6	1.4	1.1	1.7
Iran	905.6	794.0	1015.7	3.4	3.0	3.8	1498.1	1402.3	1626.4	3.2	3.0	3.4
Iraq	122.3	93.2	158.9	1.6	1.2	2.0	345.1	247.7	487.5	1.5	1.1	2.1
Ireland	37.3	33.9	41.2	2.1	1.9	2.3	42.2	31.1	57.2	1.8	1.3	2.5
Israel	46.7	41.8	51.6	1.9	1.7	2.1	81.5	59.7	108.4	1.9	1.4	2.5
Italy	1214.1	1162.7	1269.0	4.2	4.0	4.4	859.0	689.5	1050.8	3.4	2.7	4.1
Jamaica	14.8	12.9	17.1	1.3	1.1	1.5	26.5	19.3	35.6	1.7	1.3	2.3
Japan	12210.3	11848.2	12602.4	18.8	18.2	19.4	3639.2	3086.0	4284.3	7.0	6.0	8.3
Jordan	22.5	18.0	27.6	1.3	1.0	1.5	73.6	58.2	91.0	1.2	0.9	1.4
Kazakhstan	643.4	601.1	688.0	7.8	7.3	8.4	318.9	262.1	384.5	3.4	2.8	4.1
Kenya	137.5	106.4	191.6	1.4	1.0	1.9	393.1	296.7	510.8	1.5	1.1	2.0
Kiribati	2.6	2.0	3.2	7.0	5.5	8.8	3.9	2.9	5.1	6.5	4.9	8.4
Kuwait	8.2	7.1	9.3	0.8	0.7	0.9	19.2	15.4	24.0	0.7	0.5	0.8
Kyrgyzstan	182.3	168.3	197.9	8.7	8.0	9.4	144.8	120.6	172.8	4.3	3.6	5.1
Lao People's Democratic Republic	76.9	53.1	100.5	4.1	2.9	5.4	67.5	44.7	90.1	1.7	1.1	2.3
Latvia	119.6	111.1	128.1	9.3	8.6	10.0	40.0	31.0	51.0	4.9	3.8	6.2
Lebanon	31.0	23.8	38.9	2.1	1.6	2.6	53.3	39.0	70.5	2.0	1.5	2.6
Lesotho	12.4	9.2	16.1	1.5	1.1	1.9	28.0	18.5	39.1	2.4	1.6	3.4
Liberia	18.6	13.9	24.1	2.3	1.7	2.9	38.4	25.8	53.3	1.6	1.0	2.2
Libya	24.5	18.3	32.1	1.3	0.9	1.7	60.1	42.7	82.9	1.5	1.0	2.0
Lithuania	150.5	139.4	161.3	8.2	7.6	8.8	56.2	42.8	72.5	4.6	3.5	6.0
Luxembourg	6.3	5.7	7.0	3.2	2.9	3.5	4.8	3.8	6.1	1.6	1.2	2.0
Madagascar	121.5	99.9	143.1	2.3	1.9	2.7	200.0	139.6	267.0	1.5	1.1	2.0
Malawi	31.9	25.3	39.8	0.7	0.6	0.9	43.1	29.8	59.4	0.5	0.3	0.7
Malaysia	149.0	129.4	170.6	1.7	1.4	1.9	231.6	174.9	304.2	1.3	1.0	1.7
Maldives	1.6	1.1	2.0	1.7	1.2	2.1	2.0	1.6	2.4	0.6	0.5	0.8
Mali	175.5	141.3	211.7	4.9	3.9	5.9	288.8	202.1	394.6	3.0	2.1	4.1
Malta	4.1	3.6	4.6	2.1	1.9	2.4	3.3	2.7	4.1	1.7	1.4	2.1
Marshall Islands	1.1	0.9	1.3	5.3	4.3	6.3	1.9	1.3	2.6	6.4	4.4	8.4
Mauritania	20.8	16.1	26.1	2.3	1.8	2.9	20.3	12.4	30.5	1.0	0.6	1.6
Mauritius	15.3	13.8	17.1	2.5	2.3	2.8	12.6	9.6	16.4	1.9	1.5	2.5
Mexico	945.2	918.5	972.5	2.2	2.2	2.3	1906.4	1622.5	2226.2	2.9	2.4	3.4
Micronesia	3.0	2.2	4.0	6.4	4.7	8.3	3.2	1.0	4.6	5.9	1.9	8.6
Monaco	0.6	0.5	0.7	4.2	3.3	5.3	0.4	0.3	0.6	3.0	2.2	4.2
Mongolia	94.6	75.2	119.1	9.2	7.3	11.6	189.5	141.5	254.2	10.5	7.8	14.1
Montenegro	6.6	5.7	7.6	2.1	1.8	2.4	5.1	4.1	6.3	1.7	1.4	2.1
Morocco	112.2	90.0	138.1	0.9	0.7	1.1	150.8	105.3	228.2	0.8	0.5	1.2
Mozambique	48.5	36.2	61.7	0.9	0.6	1.1	110.3	73.4	158.9	0.8	0.6	1.2
Myanmar	776.1	553.8	1055.7	3.8	2.7	5.2	506.9	363.7	684.3	1.8	1.3	2.4
Namibia	4.1	2.7	5.4	0.6	0.4	0.8	7.0	4.5	10.4	0.6	0.4	0.8
Nauru	0.4	0.3	0.5	7.2	5.4	9.6	0.4	0.3	0.5	6.8	5.0	9.0
Nepal	243.8	174.6	319.9	2.7	2.0	3.6	307.7	212.2	427.8	1.9	1.3	2.6

Country	Early-onset gastric cancer (15–49 years)											
	1990						2019					
	Incidence number			Incidence rate (per 100 000)			Incidence number			Incidence rate (per 100 000)		
	Value	LUI	UUI	Value	LUI	UUI	Value	LUI	UUI	Value	LUI	UUI
Netherlands	217.8	200.0	237.4	2.7	2.5	2.9	161.6	117.4	217.7	2.2	1.6	2.9
New Zealand	46.1	41.6	51.2	2.6	2.3	2.8	40.7	31.6	51.1	2.1	1.6	2.6
Nicaragua	47.3	41.9	53.0	2.7	2.4	3.1	83.8	63.8	108.6	2.4	1.8	3.1
Niger	78.1	60.5	98.9	2.3	1.8	3.0	136.0	91.8	191.2	1.4	1.0	2.0
Nigeria	233.5	171.4	319.0	0.6	0.4	0.8	406.0	292.9	549.9	0.4	0.3	0.5
Niue	0.0	0.0	0.1	4.4	2.9	6.1	0.0	0.0	0.0	3.7	2.2	5.6
North Macedonia	54.3	48.4	60.0	5.2	4.6	5.7	40.7	30.5	52.8	3.8	2.8	4.9
Northern Mariana Islands	1.7	1.1	2.2	5.8	3.9	7.7	0.9	0.7	1.3	4.6	3.3	6.2
Norway	55.7	52.7	59.1	2.6	2.4	2.7	34.6	28.3	41.9	1.4	1.1	1.7
Oman	16.5	11.9	22.3	1.7	1.2	2.3	27.5	22.7	33.4	0.9	0.7	1.1
Pakistan	688.0	577.8	806.2	1.4	1.2	1.6	1705.5	1346.4	2162.1	1.5	1.2	1.9
Palau	0.5	0.3	0.6	5.3	3.6	7.1	0.7	0.5	0.9	7.5	5.1	10.1
Palestine	20.2	14.9	27.2	2.3	1.7	3.1	37.8	30.9	45.4	1.5	1.2	1.8
Panama	44.6	40.2	49.4	3.6	3.3	4.0	75.7	54.2	102.2	3.6	2.6	4.9
Papua New Guinea	77.4	54.3	104.3	3.9	2.7	5.2	207.7	138.8	296.7	4.1	2.7	5.8
Paraguay	37.7	32.6	43.4	2.0	1.7	2.3	70.0	51.6	93.7	1.9	1.4	2.5
Peru	520.2	442.6	606.2	4.9	4.1	5.7	786.6	553.5	1082.8	4.4	3.1	6.1
Philippines	577.5	516.9	640.2	1.9	1.7	2.1	705.5	570.0	875.1	1.2	1.0	1.5
Poland	761.4	738.7	786.2	4.0	3.9	4.1	432.4	356.3	523.9	2.4	2.0	2.9
Portugal	372.4	349.9	395.4	7.4	7.0	7.9	261.1	190.5	350.7	5.6	4.1	7.5
Puerto Rico	36.7	32.9	40.8	2.0	1.8	2.2	21.7	15.9	29.3	1.4	1.0	1.8
Qatar	3.4	2.5	4.3	1.2	0.9	1.5	16.3	12.1	21.8	0.8	0.6	1.0
Republic of Korea	4393.3	4054.5	4653.8	17.0	15.7	18.0	2690.3	2172.0	3292.7	10.3	8.3	12.6
Republic of Moldova	160.0	149.6	171.1	7.3	6.8	7.8	70.0	57.2	83.5	3.8	3.1	4.5
Romania	431.0	402.5	462.4	3.8	3.5	4.1	307.9	235.6	391.8	3.6	2.7	4.5
Russian Federation	6144.5	5606.9	6391.8	8.3	7.6	8.6	4118.2	3498.4	4828.0	6.0	5.1	7.1
Rwanda	92.4	70.4	113.4	2.9	2.2	3.6	87.2	58.4	120.3	1.3	0.9	1.9
Saint Kitts and Nevis	0.6	0.6	0.7	3.2	2.8	3.7	0.5	0.2	0.9	1.7	0.6	2.8
Saint Lucia	2.4	2.1	2.7	3.6	3.2	4.1	3.1	2.5	3.9	3.3	2.7	4.1
Saint Vincent and the Grenadines	1.5	1.3	1.7	2.9	2.5	3.3	1.7	1.4	2.0	3.0	2.5	3.6
Samoa	2.0	1.4	2.9	2.6	1.8	3.7	2.9	1.8	4.2	2.8	1.7	4.0
San Marino	0.7	0.6	0.9	5.9	4.7	7.3	0.9	0.6	1.2	5.8	4.1	7.8
Sao Tome and Principe	0.8	0.5	1.1	1.6	1.1	2.2	1.9	1.4	2.7	1.8	1.3	2.5
Saudi Arabia	62.5	43.9	84.8	0.8	0.5	1.0	244.6	175.6	342.3	1.0	0.7	1.4
Senegal	69.1	52.5	85.1	2.1	1.6	2.6	108.2	72.7	147.2	1.5	1.0	2.0
Serbia	175.9	152.0	201.2	3.8	3.3	4.4	111.0	82.8	145.8	2.8	2.1	3.6
Seychelles	0.9	0.8	1.1	2.5	2.1	2.9	1.2	1.0	1.5	2.3	1.9	2.8
Sierra Leone	33.5	23.7	45.2	2.0	1.4	2.7	67.0	46.5	93.8	1.6	1.1	2.3
Singapore	80.5	72.2	89.6	4.2	3.8	4.7	66.9	49.7	86.2	2.2	1.6	2.8
Slovakia	124.7	113.3	136.7	4.7	4.2	5.1	79.7	58.7	105.3	3.0	2.2	4.0
Slovenia	51.1	37.8	69.9	5.0	3.7	6.9	29.3	21.4	39.2	3.3	2.4	4.4
Solomon Islands	13.8	9.0	18.2	9.1	6.0	12.0	35.1	24.0	46.4	10.7	7.3	14.1
Somalia	100.9	70.6	138.1	3.2	2.3	4.4	210.8	130.0	303.1	2.2	1.4	3.2
South Africa	446.3	406.0	485.8	2.3	2.1	2.6	333.5	292.4	381.5	1.1	1.0	1.3
South Sudan	42.7	28.0	63.6	1.6	1.0	2.3	53.3	34.2	83.2	1.3	0.8	2.0
Spain	819.1	760.2	877.0	4.2	3.9	4.5	760.3	554.1	1004.8	3.7	2.7	4.9
Sri Lanka	152.7	132.1	173.6	1.7	1.4	1.9	133.2	95.5	182.5	1.2	0.9	1.7
Sudan	317.7	203.1	414.5	3.5	2.2	4.6	575.8	336.7	854.7	2.8	1.6	4.1
Suriname	3.6	2.8	4.2	1.8	1.4	2.1	5.3	4.2	6.7	1.8	1.4	2.3
Sweden	97.5	90.2	105.7	2.3	2.1	2.5	45.5	37.1	54.5	1.0	0.8	1.2
Switzerland	123.4	112.2	136.1	3.4	3.1	3.8	64.8	46.0	87.6	1.6	1.2	2.2
Syrian Arab Republic	78.7	60.1	100.4	1.4	1.1	1.8	92.6	64.3	131.2	1.2	0.9	1.8
Tajikistan	160.1	144.2	181.4	6.6	6.0	7.5	253.9	199.6	324.7	5.1	4.0	6.5
Thailand	914.5	774.8	1072.4	2.9	2.5	3.4	968.0	682.9	1312.9	2.7	1.9	3.7
Timor-Leste	8.3	5.3	11.5	2.2	1.4	3.0	7.6	2.4	11.2	1.2	0.4	1.7
Togo	35.0	27.5	43.8	2.2	1.7	2.7	74.4	50.3	103.5	1.9	1.3	2.6
Tokelau	0.0	0.0	0.0	3.6	2.4	5.2	0.0	0.0	0.0	3.2	2.0	4.6
Tonga	1.6	1.3	2.0	3.6	2.9	4.6	1.9	1.4	2.6	3.9	2.8	5.4
Trinidad and Tobago	9.8	8.8	10.8	1.6	1.4	1.7	6.7	4.7	9.3	1.0	0.7	1.3
Tunisia	44.7	36.2	55.1	1.1	0.9	1.3	79.0	54.6	108.9	1.3	0.9	1.8
Turkey	1595.0	1295.9	1888.8	5.3	4.3	6.3	1478.5	1143.3	1878.5	3.2	2.5	4.1
Turkmenistan	72.4	66.7	78.7	4.1	3.8	4.5	66.0	51.3	86.1	2.5	1.9	3.3
Tuvalu	0.3	0.2	0.4	6.0	4.5	8.0	0.3	0.2	0.4	4.8	3.2	6.6

Country	Early-onset gastric cancer (15–49 years)											
	1990						2019					
	Incidence number			Incidence rate (per 100 000)			Incidence number			Incidence rate (per 100 000)		
	Value	LUI	UUI	Value	LUI	UUI	Value	LUI	UUI	Value	LUI	UUI
Uganda	116.1	83.6	150.7	1.6	1.1	2.0	293.4	212.2	406.9	1.6	1.1	2.2
Ukraine	2717.5	2552.3	2888.0	10.9	10.2	11.6	1460.2	1183.2	1796.5	7.0	5.7	8.6
United Arab Emirates	13.4	9.9	17.8	1.1	0.8	1.5	93.2	64.0	131.6	1.3	0.9	1.9
United Kingdom	543.0	530.3	558.0	1.9	1.9	2.0	459.4	377.9	557.1	1.5	1.2	1.8
United Republic of Tanzania	199.1	152.6	254.2	1.8	1.3	2.2	366.2	264.3	492.6	1.4	1.0	1.8
United States of America	2188.2	2126.4	2245.8	1.6	1.6	1.7	2453.3	2087.7	2903.0	1.6	1.4	1.9
United States Virgin Islands	1.5	1.2	1.8	2.7	2.2	3.3	1.0	0.7	1.5	2.4	1.7	3.4
Uruguay	55.5	50.7	60.9	3.7	3.4	4.1	42.7	31.3	56.8	2.6	1.9	3.4
Uzbekistan	491.0	456.1	524.1	5.0	4.7	5.4	632.6	513.9	771.8	3.5	2.8	4.3
Vanuatu	3.2	2.0	4.4	4.6	2.9	6.3	7.5	4.8	10.4	5.2	3.3	7.1
Venezuela	285.7	264.9	308.4	3.0	2.8	3.2	518.6	374.1	706.1	3.6	2.6	4.9
Viet Nam	943.0	686.0	1251.7	2.9	2.1	3.8	1585.7	1141.4	2108.0	3.0	2.2	4.0
Yemen	207.3	124.7	294.6	3.8	2.3	5.4	537.0	344.5	770.2	3.4	2.2	4.9
Zambia	77.5	61.3	95.6	2.2	1.7	2.7	156.7	106.0	218.9	1.7	1.2	2.4
Zimbabwe	83.0	67.8	100.1	1.8	1.5	2.2	215.6	148.4	291.7	2.9	2.0	3.9

LUI, lower uncertainty intervals; UUI, upper uncertainty intervals

Table S3. Incidence of intermediate-onset gastric cancer in 203 countries, 2019 versus 1990

Country	Intermediate-onset gastric cancer (50–54 years)											
	1990						2019					
	Incidence number			Incidence rate (per 100 000)			Incidence number			Incidence rate (per 100 000)		
	Value	LUI	UUI	Value	LUI	UUI	Value	LUI	UUI	Value	LUI	UUI
Afghanistan	230.4	132.9	331.1	54.2	31.2	77.8	553.4	317.0	796.5	46.8	26.8	67.4
Albania	26.7	23.0	30.6	20.7	17.8	23.7	26.2	17.5	37.0	14.2	9.5	20.1
Algeria	68.4	48.3	94.2	9.8	6.9	13.5	113.5	78.5	157.2	5.5	3.8	7.6
American Samoa	0.4	0.3	0.5	26.8	19.7	37.0	0.7	0.5	0.9	20.8	14.9	28.2
Andorra	0.4	0.3	0.6	16.1	10.8	22.9	1.0	0.6	1.4	13.3	8.7	19.3
Angola	69.4	46.8	101.2	25.0	16.9	36.5	107.0	73.1	153.4	13.1	8.9	18.7
Antigua and Barbuda	0.5	0.4	0.6	23.5	18.5	29.4	0.8	0.6	1.0	12.2	9.1	16.1
Argentina	280.5	249.4	314.9	18.5	16.4	20.8	314.7	222.4	441.3	13.8	9.7	19.3
Armenia	74.9	67.1	82.8	41.0	36.8	45.4	30.8	23.8	39.3	18.2	14.0	23.2
Australia	94.0	83.5	106.0	11.7	10.4	13.2	121.5	83.0	175.4	8.0	5.4	11.5
Austria	92.0	81.9	103.0	20.6	18.3	23.0	67.5	48.6	91.4	9.5	6.8	12.8
Azerbaijan	202.3	174.8	237.1	56.6	48.9	66.3	180.2	124.9	247.8	27.2	18.8	37.4
Bahamas	2.1	1.7	2.5	23.1	18.9	27.8	3.3	2.5	4.4	13.4	10.1	17.8
Bahrain	2.0	1.4	2.6	14.5	10.7	19.1	4.3	3.0	6.1	4.3	3.0	6.1
Bangladesh	547.9	389.4	742.5	20.6	14.7	27.9	779.3	531.3	1115.2	10.8	7.4	15.5
Barbados	1.8	1.5	2.1	18.8	16.0	22.0	2.5	1.9	3.3	11.9	8.8	15.4
Belarus	470.8	429.6	521.0	68.8	62.8	76.1	182.2	125.8	255.5	28.7	19.8	40.3
Belgium	69.0	60.5	77.9	11.7	10.2	13.2	63.1	44.0	88.7	7.9	5.5	11.1
Belize	0.7	0.6	0.9	16.0	13.1	19.2	2.7	2.2	3.4	15.6	12.4	19.2
Benin	23.3	16.6	31.3	22.0	15.7	29.6	46.2	30.2	69.9	14.7	9.6	22.3
Bermuda	0.5	0.4	0.6	16.8	13.1	21.1	0.4	0.3	0.6	7.9	5.7	10.8
Bhutan	2.6	1.6	3.8	16.2	9.9	23.9	3.2	2.0	4.8	10.2	6.4	15.3
Bolivia	107.5	77.6	140.7	59.6	43.0	78.0	168.8	107.6	246.3	34.8	22.2	50.8
Bosnia and Herzegovina	55.6	48.8	63.3	20.2	17.7	23.0	34.6	24.6	46.2	14.2	10.1	19.0
Botswana	6.0	3.7	8.9	18.0	11.2	26.7	11.6	7.3	17.5	13.4	8.4	20.2
Brazil	1400.5	1334.2	1476.8	27.1	25.8	28.6	1825.4	1709.8	1950.4	14.4	13.5	15.4
Brunei Darussalam	2.6	2.0	3.3	38.7	29.7	49.7	4.4	3.4	5.7	18.3	14.1	23.8
Bulgaria	148.8	133.2	165.3	28.4	25.4	31.6	85.6	59.5	118.3	17.9	12.4	24.7
Burkina Faso	55.9	38.5	79.9	21.9	15.1	31.4	102.0	71.5	144.9	17.2	12.1	24.5
Burundi	30.5	20.9	43.3	23.4	16.0	33.2	41.8	26.7	62.7	13.7	8.7	20.5
Cabo Verde	3.8	2.9	5.0	39.6	30.0	52.2	6.2	4.3	8.6	24.5	17.1	34.3
Cambodia	72.4	51.1	99.3	26.2	18.5	36.0	96.4	64.9	136.2	12.8	8.6	18.0
Cameroon	61.5	44.1	82.9	21.4	15.4	28.9	120.3	75.9	180.9	15.4	9.7	23.2
Canada	160.2	141.5	183.1	12.3	10.9	14.1	230.5	152.7	322.6	9.3	6.2	13.1
Central African Republic	25.7	18.2	35.2	33.2	23.5	45.4	40.2	24.5	60.0	24.3	14.8	36.2
Chad	30.8	22.6	42.2	21.2	15.5	29.0	67.6	44.5	97.5	18.7	12.3	27.0
Chile	216.0	194.4	240.7	41.4	37.2	46.1	238.5	167.2	326.7	20.2	14.1	27.6
China	28590.4	24242.7	33603.5	59.8	50.7	70.3	49663.8	39634.6	60809.4	39.7	31.7	48.6
Colombia	384.5	349.6	423.7	37.0	33.6	40.8	552.7	389.2	768.0	20.0	14.1	27.8
Comoros	1.8	0.6	2.9	14.4	5.1	23.4	2.8	1.8	4.1	9.6	6.1	14.1
Congo	17.9	12.5	24.5	27.6	19.3	37.7	24.0	15.8	35.2	12.6	8.3	18.4
Cook Islands	0.1	0.1	0.1	15.5	11.1	21.1	0.1	0.1	0.2	10.3	6.9	14.4
Costa Rica	46.4	40.5	52.6	50.6	44.1	57.3	77.7	52.3	108.4	28.6	19.2	39.9
Croatia	107.7	94.1	121.9	33.7	29.4	38.1	45.1	31.8	60.8	14.8	10.4	20.0
Cuba	54.9	48.7	61.8	11.1	9.9	12.5	90.2	66.0	119.5	8.9	6.5	11.7
Cyprus	3.6	2.7	4.7	9.9	7.5	12.9	7.2	5.3	9.7	8.9	6.6	12.1
Czechia	114.2	103.6	125.9	22.1	20.0	24.4	63.7	47.1	84.1	9.3	6.9	12.2
Cote d'Ivoire	71.9	51.0	99.0	24.8	17.6	34.2	118.5	77.8	171.9	15.9	10.4	23.0
Democratic People's Republic of Korea	752.7	481.7	1130.1	61.6	39.4	92.5	851.0	552.3	1261.3	42.0	27.3	62.3
Democratic Republic of the Congo	182.8	124.8	257.0	18.0	12.3	25.3	316.5	206.3	462.2	12.7	8.3	18.5
Denmark	43.9	38.2	50.0	15.5	13.5	17.7	30.0	19.8	42.9	7.2	4.7	10.2
Djibouti	1.6	1.0	2.3	14.7	9.2	21.4	5.0	3.0	7.9	11.0	6.7	17.4
Dominica	0.9	0.7	1.1	35.6	27.4	45.2	1.1	0.8	1.5	23.3	16.4	31.6
Dominican Republic	23.2	18.6	29.0	11.1	8.9	13.9	69.9	45.1	104.5	13.4	8.7	20.1
Ecuador	107.9	96.2	119.4	36.1	32.2	40.0	189.8	137.7	263.3	23.7	17.2	32.9
Egypt	135.4	103.9	172.9	7.4	5.7	9.5	271.8	173.3	403.3	6.5	4.2	9.7
El Salvador	39.6	34.7	44.9	25.3	22.2	28.7	68.0	46.6	97.0	23.6	16.2	33.7
Equatorial Guinea	3.5	2.2	5.2	26.6	17.2	39.6	2.5	1.4	4.2	7.8	4.4	12.9
Eritrea	18.6	12.6	25.9	24.1	16.2	33.5	35.0	22.3	51.9	17.8	11.3	26.3
Estonia	51.7	45.9	57.9	50.9	45.2	57.0	19.6	12.9	28.1	24.0	15.8	34.4
Eswatini	3.3	2.3	4.8	17.2	11.8	24.7	5.1	3.1	7.8	14.9	9.2	22.8
Ethiopia	370.0	283.4	462.8	28.8	22.1	36.0	225.8	170.0	302.0	8.9	6.7	11.9

Country	Intermediate-onset gastric cancer (50–54 years)											
	1990						2019					
	Incidence number			Incidence rate (per 100 000)			Incidence number			Incidence rate (per 100 000)		
	Value	LUI	UUI	Value	LUI	UUI	Value	LUI	UUI	Value	LUI	UUI
Fiji	3.0	2.1	4.2	11.8	8.3	16.6	4.6	3.1	6.6	9.6	6.4	13.5
Finland	51.2	45.0	58.4	18.6	16.3	21.2	29.9	19.5	43.4	8.4	5.5	12.2
France	349.1	309.8	391.8	11.8	10.5	13.2	390.7	258.6	556.4	8.9	5.9	12.7
Gabon	6.6	4.7	9.3	21.3	15.0	30.0	7.9	5.2	11.4	11.4	7.5	16.4
Gambia	1.5	0.9	2.3	6.9	4.3	10.4	3.1	2.0	4.6	5.6	3.5	8.1
Georgia	137.8	118.4	161.3	38.1	32.7	44.6	55.4	41.0	72.7	24.2	17.9	31.8
Germany	1130.9	1005.3	1266.9	18.5	16.4	20.7	855.5	582.3	1202.0	12.6	8.6	17.7
Ghana	56.0	39.1	77.6	14.1	9.9	19.6	87.0	58.4	126.3	8.5	5.7	12.3
Greece	129.7	116.6	144.2	20.2	18.2	22.5	105.9	76.1	145.6	13.9	10.0	19.1
Greenland	0.8	0.6	1.0	32.1	24.0	42.8	0.9	0.6	1.2	17.3	12.1	24.0
Grenada	0.6	0.4	0.7	23.1	18.4	28.5	0.8	0.7	1.1	11.7	9.1	14.9
Guam	0.4	0.3	0.6	8.7	6.5	11.4	1.0	0.7	1.4	9.8	6.7	13.6
Guatemala	82.0	69.1	97.2	34.8	29.4	41.3	202.4	149.5	268.3	35.5	26.2	47.0
Guinea	39.9	29.2	54.1	23.2	17.0	31.5	68.8	46.0	95.6	21.6	14.5	30.1
Guinea-Bissau	10.1	6.8	14.3	42.1	28.4	59.7	12.4	8.4	17.6	26.1	17.6	37.0
Guyana	4.2	3.2	5.3	19.4	15.0	24.6	5.4	3.7	7.6	13.8	9.5	19.4
Haiti	72.3	47.1	99.2	37.5	24.4	51.5	96.4	57.6	145.9	22.3	13.3	33.7
Honduras	23.4	17.3	30.8	19.6	14.5	25.9	68.7	43.8	103.2	20.1	12.8	30.3
Hungary	147.2	132.3	163.0	24.6	22.1	27.2	88.5	63.6	120.1	13.6	9.8	18.5
Iceland	1.8	1.4	2.3	17.8	13.8	22.5	1.9	1.4	2.5	8.6	6.3	11.8
India	5108.1	4445.2	5768.1	17.3	15.1	19.6	8031.8	6531.7	9769.6	12.6	10.3	15.3
Indonesia	887.6	717.8	1035.7	13.5	10.9	15.8	1157.8	903.1	1434.6	7.8	6.1	9.7
Iran	491.3	422.1	544.0	31.0	26.7	34.4	809.6	742.5	889.0	18.0	16.5	19.8
Iraq	55.1	37.9	76.5	12.3	8.4	17.0	147.1	98.8	206.5	9.5	6.4	13.3
Ireland	22.6	19.4	26.3	14.4	12.4	16.7	25.4	17.0	36.0	8.1	5.4	11.5
Israel	22.2	19.3	25.5	11.7	10.2	13.4	38.5	26.6	53.9	8.7	6.0	12.2
Italy	852.9	799.8	912.6	23.5	22.0	25.1	690.9	539.1	873.7	14.0	10.9	17.7
Jamaica	11.4	9.0	14.0	15.9	12.6	19.5	19.7	13.3	27.8	12.8	8.7	18.1
Japan	7100.1	6763.4	7425.3	86.5	82.4	90.4	2526.8	2096.7	3040.4	29.1	24.2	35.0
Jordan	11.6	8.4	15.9	11.0	8.0	15.0	31.1	22.2	42.5	6.1	4.3	8.3
Kazakhstan	568.5	514.9	625.4	64.0	58.0	70.4	213.8	166.5	269.7	21.5	16.8	27.1
Kenya	61.6	47.1	82.4	12.6	9.7	16.9	178.6	135.8	223.1	12.1	9.2	15.0
Kiribati	0.7	0.5	1.0	33.2	22.7	45.4	1.2	0.8	1.7	25.8	16.6	37.0
Kuwait	3.1	2.4	3.9	6.4	5.0	8.1	8.6	5.8	12.0	3.3	2.3	4.7
Kyrgyzstan	121.6	107.1	135.7	68.8	60.6	76.8	87.3	68.0	107.9	29.2	22.7	36.1
Lao People's Democratic Republic	37.8	24.8	54.2	30.3	19.9	43.4	31.5	20.4	45.8	10.9	7.1	15.8
Latvia	84.1	76.3	91.8	47.1	42.7	51.4	27.2	20.4	35.5	21.1	15.8	27.5
Lebanon	17.4	12.7	23.4	13.0	9.4	17.4	25.3	17.5	35.5	10.0	6.9	14.1
Lesotho	8.7	5.9	12.7	16.3	10.9	23.6	15.4	9.8	22.7	21.2	13.5	31.2
Liberia	11.3	7.7	15.9	20.1	13.7	28.2	17.3	10.9	26.5	12.8	8.0	19.6
Libya	11.2	7.6	15.9	9.9	6.7	14.0	27.4	17.8	40.1	7.4	4.8	10.9
Lithuania	100.5	89.6	111.5	45.6	40.7	50.6	43.5	31.1	60.0	21.4	15.3	29.6
Luxembourg	3.8	3.1	4.6	16.5	13.5	20.1	3.2	2.3	4.3	6.8	4.9	9.0
Madagascar	43.2	31.7	56.9	14.7	10.7	19.3	83.4	54.1	121.5	10.4	6.7	15.1
Malawi	12.8	9.4	17.8	5.7	4.2	7.9	16.8	11.1	23.6	4.0	2.6	5.6
Malaysia	76.0	59.5	94.7	12.5	9.7	15.5	116.8	81.2	161.2	7.7	5.3	10.6
Maldives	1.0	0.7	1.3	14.3	10.1	19.2	0.8	0.6	1.0	3.6	2.6	4.7
Mali	99.0	73.3	130.6	40.8	30.2	53.8	150.2	101.9	215.2	27.9	18.9	39.9
Malta	2.4	2.0	2.9	12.6	10.3	15.2	1.7	1.3	2.3	6.8	5.1	8.8
Marshall Islands	0.3	0.2	0.4	30.7	22.0	41.4	0.6	0.4	0.9	25.1	17.0	35.6
Mauritania	12.9	9.2	17.3	24.7	17.7	33.1	12.3	7.6	18.2	10.1	6.3	15.0
Mauritius	6.8	5.8	8.0	17.6	14.9	20.5	8.1	6.0	10.9	8.8	6.5	11.8
Mexico	375.4	361.8	389.1	15.1	14.5	15.6	897.4	750.8	1064.2	13.5	11.3	16.0
Micronesia	0.7	0.5	1.0	31.9	22.0	45.1	1.3	0.9	1.9	27.6	18.4	39.6
Monaco	0.3	0.2	0.5	16.9	11.8	23.2	0.4	0.2	0.5	11.0	7.3	15.4
Mongolia	60.9	44.7	80.1	107.9	79.1	141.9	116.8	79.3	164.5	64.1	43.6	90.3
Montenegro	4.6	3.7	5.7	12.7	10.1	15.5	4.4	3.3	5.7	10.7	8.0	13.8
Morocco	52.7	37.7	70.2	6.7	4.8	8.9	93.7	61.7	137.2	4.9	3.2	7.2
Mozambique	31.5	21.7	43.5	8.0	5.5	11.0	60.1	37.6	86.7	8.5	5.3	12.3
Myanmar	339.3	231.8	487.3	25.5	17.4	36.6	279.7	198.1	378.6	9.7	6.9	13.2
Namibia	2.1	1.4	3.0	5.8	3.8	8.1	3.6	2.4	5.2	4.3	2.9	6.2
Nauru	0.1	0.1	0.1	34.3	22.7	49.7	0.1	0.1	0.1	28.2	19.0	40.4
Nepal	105.9	71.7	145.4	17.5	11.9	24.1	148.4	99.5	209.3	12.0	8.0	16.9

Country	Intermediate-onset gastric cancer (50–54 years)											
	1990						2019					
	Incidence number			Incidence rate (per 100 000)			Incidence number			Incidence rate (per 100 000)		
	Value	LUI	UUI	Value	LUI	UUI	Value	LUI	UUI	Value	LUI	UUI
Netherlands	123.4	109.0	139.5	15.8	14.0	17.9	127.9	87.4	176.0	10.1	6.9	13.8
New Zealand	20.8	17.7	24.1	13.5	11.5	15.6	25.4	18.8	32.8	8.5	6.3	11.0
Nicaragua	18.2	14.7	22.1	20.4	16.5	24.8	46.8	33.9	64.2	17.5	12.7	24.1
Niger	41.7	29.1	56.9	22.9	16.0	31.2	79.0	52.2	119.8	15.7	10.4	23.9
Nigeria	143.8	104.5	194.0	5.5	4.0	7.4	233.4	171.3	313.1	3.7	2.7	5.0
Niue	0.0	0.0	0.0	18.9	12.9	26.3	0.0	0.0	0.0	15.0	9.8	21.4
North Macedonia	33.2	28.0	38.7	31.7	26.8	37.0	29.5	21.1	40.5	19.5	14.0	26.7
Northern Mariana Islands	0.4	0.3	0.5	25.0	17.8	34.4	0.7	0.5	1.0	15.9	11.0	21.6
Norway	28.0	25.9	30.3	14.1	13.0	15.3	25.5	20.2	31.5	6.9	5.5	8.6
Oman	8.4	5.5	12.4	16.7	10.9	24.7	10.2	7.2	14.4	6.7	4.7	9.4
Pakistan	310.4	242.1	391.2	9.3	7.3	11.7	687.3	518.2	915.7	9.3	7.0	12.4
Palau	0.1	0.1	0.2	22.7	15.1	34.3	0.3	0.2	0.5	20.4	13.4	30.7
Palestine	7.6	5.1	11.0	16.5	11.1	23.7	17.1	12.6	22.4	9.8	7.2	12.8
Panama	19.0	16.1	21.9	23.5	20.0	27.2	36.4	25.0	50.3	16.6	11.4	23.0
Papua New Guinea	25.0	15.5	37.7	20.9	13.0	31.6	70.2	42.4	106.3	19.7	11.9	29.8
Paraguay	16.8	13.2	21.2	14.0	11.1	17.7	38.5	25.6	54.8	12.7	8.4	18.1
Peru	214.1	172.4	265.8	31.9	25.7	39.6	343.1	228.9	510.4	20.1	13.4	29.8
Philippines	217.5	190.0	249.2	11.4	9.9	13.0	327.0	256.4	414.4	6.5	5.1	8.3
Poland	550.5	529.4	570.3	28.6	27.5	29.6	308.7	249.3	386.9	13.5	10.9	16.9
Portugal	211.6	190.1	234.5	36.4	32.7	40.3	196.3	138.0	269.3	25.2	17.7	34.6
Puerto Rico	20.5	17.6	23.7	12.5	10.7	14.4	16.7	11.4	23.2	7.0	4.8	9.8
Qatar	1.3	0.9	1.8	10.2	7.2	14.1	5.2	3.4	7.6	4.3	2.8	6.3
Republic of Korea	2421.3	2155.9	2715.5	118.7	105.7	133.1	1661.7	1239.2	2151.1	37.2	27.8	48.2
Republic of Moldova	102.1	90.1	115.8	42.1	37.1	47.7	42.2	32.4	53.0	18.1	13.9	22.7
Romania	359.5	318.6	402.0	24.5	21.7	27.4	265.7	193.9	348.9	18.9	13.8	24.8
Russian Federation	6254.7	5861.6	6462.6	59.5	55.8	61.5	2303.3	1963.3	2720.5	26.2	22.3	31.0
Rwanda	41.2	29.9	54.6	24.2	17.5	32.0	37.0	24.6	53.7	10.0	6.6	14.4
Saint Kitts and Nevis	0.3	0.2	0.3	24.0	19.3	29.8	0.6	0.4	0.8	13.8	9.9	18.9
Saint Lucia	1.1	0.9	1.3	27.8	22.5	34.4	2.0	1.5	2.7	16.4	12.0	21.6
Saint Vincent and the Grenadines	0.6	0.5	0.7	19.9	15.9	24.4	1.2	1.0	1.6	17.3	13.3	22.1
Samoa	1.2	0.8	1.7	24.1	16.0	34.3	1.7	1.1	2.5	18.8	12.4	27.4
San Marino	0.5	0.3	0.6	34.3	25.3	45.1	0.7	0.4	1.0	26.7	17.4	39.1
Sao Tome and Principe	0.7	0.5	1.0	20.7	13.3	28.9	1.2	0.8	1.8	17.9	11.9	25.5
Saudi Arabia	28.2	18.8	40.7	7.1	4.7	10.3	86.0	58.1	122.5	5.3	3.6	7.6
Senegal	40.3	27.9	54.5	21.4	14.9	29.0	63.8	42.7	91.1	14.2	9.5	20.3
Serbia	128.5	99.3	164.1	20.9	16.1	26.6	87.8	62.3	122.1	16.1	11.5	22.5
Seychelles	0.5	0.3	0.6	18.1	13.5	23.6	0.7	0.5	1.0	10.5	7.7	14.0
Sierra Leone	20.3	13.7	28.7	19.8	13.3	28.0	33.8	22.2	49.6	15.2	10.0	22.3
Singapore	38.8	31.8	46.9	29.0	23.8	35.1	42.5	29.5	60.2	10.1	7.0	14.4
Slovakia	76.7	66.8	88.3	30.9	26.9	35.5	48.8	33.8	68.8	14.0	9.7	19.7
Slovenia	37.5	26.7	51.6	32.6	23.2	44.9	24.4	16.1	35.0	15.9	10.5	22.8
Solomon Islands	4.6	2.8	6.6	49.4	30.4	71.6	9.7	6.2	13.5	44.0	28.2	61.5
Somalia	46.8	29.5	69.5	24.8	15.6	36.8	65.4	40.5	95.2	18.2	11.3	26.5
South Africa	155.1	134.7	176.6	13.7	11.9	15.6	191.8	157.0	229.2	7.6	6.2	9.0
South Sudan	19.6	11.9	30.4	14.4	8.8	22.3	26.7	16.3	41.0	9.3	5.7	14.2
Spain	477.7	426.3	539.0	24.3	21.7	27.4	520.0	351.8	731.8	14.5	9.8	20.4
Sri Lanka	77.1	59.6	96.7	12.1	9.4	15.2	92.1	60.3	135.4	7.0	4.6	10.4
Sudan	137.8	86.1	189.2	26.1	16.3	35.8	245.7	138.5	380.5	19.6	11.0	30.3
Suriname	2.3	1.7	2.8	14.9	11.4	18.5	3.9	2.7	5.4	10.4	7.3	14.7
Sweden	50.6	45.2	56.7	10.8	9.7	12.1	35.9	27.6	45.7	5.3	4.1	6.8
Switzerland	66.9	57.8	77.8	17.0	14.7	19.8	51.7	33.8	76.4	7.7	5.0	11.4
Syrian Arab Republic	25.1	18.5	33.4	8.1	6.0	10.8	50.2	33.6	73.1	5.8	3.9	8.5
Tajikistan	105.3	91.3	122.7	62.6	54.3	73.0	115.1	83.6	159.3	29.2	21.2	40.4
Thailand	433.9	337.3	539.9	18.8	14.6	23.3	525.9	344.2	783.2	9.3	6.1	13.9
Timor-Leste	3.6	2.5	5.2	16.4	11.0	23.6	3.9	2.2	5.9	9.5	5.4	14.3
Togo	17.6	12.4	25.0	21.8	15.4	31.1	42.8	27.1	63.0	16.5	10.4	24.2
Tokelau	0.0	0.0	0.0	18.6	12.4	26.9	0.0	0.0	0.0	12.7	8.3	18.6
Tonga	0.7	0.5	0.9	21.0	14.9	28.3	0.8	0.5	1.2	17.7	11.6	26.2
Trinidad and Tobago	5.5	4.5	6.6	12.8	10.5	15.4	4.5	2.9	6.4	5.2	3.4	7.4
Tunisia	23.4	17.0	32.7	8.4	6.1	11.8	43.3	27.4	62.7	6.3	4.0	9.2
Turkey	696.5	513.9	913.9	33.6	24.8	44.1	859.6	623.6	1166.8	18.0	13.1	24.4
Turkmenistan	47.6	41.9	54.3	41.3	36.3	47.1	33.5	23.9	46.3	13.1	9.3	18.1
Tuvalu	0.1	0.1	0.1	28.0	19.8	38.1	0.1	0.1	0.2	19.7	12.8	29.7

Country	Intermediate-onset gastric cancer (50–54 years)											
	1990						2019					
	Incidence number			Incidence rate (per 100 000)			Incidence number			Incidence rate (per 100 000)		
	Value	LUI	UUI	Value	LUI	UUI	Value	LUI	UUI	Value	LUI	UUI
Uganda	56.2	39.0	77.5	14.2	9.9	19.6	119.5	78.6	171.7	12.8	8.4	18.3
Ukraine	2494.1	2265.8	2745.2	63.6	57.8	70.0	906.9	700.8	1179.6	31.5	24.3	41.0
United Arab Emirates	5.9	4.0	8.3	12.5	8.6	17.8	40.6	25.4	60.9	7.2	4.5	10.9
United Kingdom	390.7	379.1	402.1	12.5	12.1	12.9	327.1	262.3	410.1	7.0	5.6	8.8
United Republic of Tanzania	98.3	69.4	135.5	15.1	10.7	20.8	167.6	109.5	242.2	10.5	6.8	15.1
United States of America	1137.4	1098.0	1176.8	9.9	9.6	10.3	1640.7	1359.3	1971.5	7.9	6.5	9.5
United States Virgin Islands	0.8	0.6	1.1	15.0	11.1	20.2	1.2	0.8	1.7	15.6	10.8	22.3
Uruguay	39.2	33.9	45.1	24.5	21.1	28.1	29.7	21.1	40.7	15.1	10.7	20.6
Uzbekistan	299.0	268.3	335.1	44.9	40.3	50.3	325.1	249.1	411.4	20.4	15.6	25.8
Vanuatu	0.9	0.6	1.5	23.7	14.8	36.9	2.3	1.5	3.5	23.2	14.6	34.5
Venezuela	139.3	123.3	156.2	25.0	22.1	28.0	289.4	195.1	409.1	18.1	12.2	25.7
Viet Nam	571.0	394.4	796.1	29.2	20.1	40.6	1082.1	736.3	1530.0	18.4	12.5	26.0
Yemen	102.2	64.0	155.4	34.5	21.6	52.4	225.0	137.4	345.8	28.0	17.1	43.0
Zambia	34.0	24.3	45.0	18.0	12.9	23.8	60.5	40.2	86.8	13.3	8.8	19.1
Zimbabwe	45.6	35.4	59.0	18.0	14.0	23.3	83.8	56.9	117.5	20.5	13.9	28.8

LUI, lower uncertainty intervals; UUI, upper uncertainty intervals

Table S4. Incidence of later-onset gastric cancer in 203 countries, 2019 versus 1990

Country	Later-onset gastric cancer (≥ 55 years)											
	1990						2019					
	Incidence number			Incidence rate[a]			Incidence number			Incidence rate[a]		
	Value	LUI	UUI	Value	LUI	UUI	Value	LUI	UUI	Value	LUI	UUI
Afghanistan	1541.3	1135.9	1949.7	124.3	91.6	157.3	1919.4	1414.9	2378.8	117.2	86.4	145.2
Albania	245.1	227.8	262.2	70.5	65.5	75.4	418.7	314.8	554.1	54.4	40.9	72.0
Algeria	698.7	561.0	856.7	33.5	26.9	41.1	1193.3	965.9	1469.0	21.2	17.2	26.1
American Samoa	2.7	2.4	3.1	73.5	64.6	83.9	5.1	4.4	5.9	61.3	52.6	71.2
Andorra	6.4	5.0	8.7	66.7	51.8	90.8	14.6	11.1	18.7	60.7	46.1	78.0
Angola	346.6	268.2	437.8	56.2	43.5	71.0	618.2	496.7	777.2	34.3	27.6	43.1
Antigua and Barbuda	7.4	6.7	8.2	86.3	78.0	94.6	9.4	8.0	10.8	53.1	45.2	61.4
Argentina	3762.2	3588.0	3934.2	67.6	64.4	70.7	4454.5	3549.8	5559.0	49.2	39.2	61.3
Armenia	461.5	437.8	487.3	95.6	90.7	100.9	471.8	392.7	554.4	61.8	51.4	72.6
Australia	1695.2	1606.4	1777.0	51.7	49.0	54.2	2594.5	2023.1	3278.3	37.5	29.3	47.4
Austria	2187.1	2077.6	2292.9	112.3	106.7	117.7	1386.4	1124.7	1672.3	48.2	39.1	58.1
Azerbaijan	1095.2	1007.8	1184.3	125.7	115.7	135.9	1505.0	1241.4	1844.2	87.3	72.0	106.9
Bahamas	15.4	13.9	17.1	62.9	56.9	69.6	26.6	21.6	32.4	39.4	31.9	48.0
Bahrain	15.4	13.1	17.8	54.5	46.4	63.1	35.2	28.0	44.3	19.2	15.2	24.1
Bangladesh	3571.4	2977.9	4156.4	47.6	39.7	55.4	6087.0	4660.3	7993.5	27.9	21.3	36.6
Barbados	41.9	37.9	45.7	89.3	80.9	97.5	47.0	38.7	55.4	54.1	44.6	63.9
Belarus	3509.6	3366.0	3658.0	151.2	145.0	157.6	2318.1	1822.9	2952.3	80.9	63.6	103.0
Belgium	1858.1	1736.5	1964.5	70.8	66.2	74.9	1505.3	1206.0	1883.6	41.0	32.8	51.2
Belize	9.2	8.2	10.3	59.9	53.3	66.8	20.0	17.2	23.0	43.7	37.5	50.4
Benin	241.0	208.3	279.5	75.3	65.1	87.3	424.5	339.5	522.3	55.4	44.3	68.1
Bermuda	6.8	6.2	7.5	64.2	58.3	70.4	7.9	6.6	9.5	35.4	29.6	42.3
Bhutan	14.8	10.7	19.5	37.3	26.8	49.0	33.5	24.9	43.1	36.1	26.8	46.4
Bolivia	1083.1	893.1	1266.9	206.4	170.2	241.4	2388.2	1894.9	2958.9	160.1	127.0	198.3
Bosnia and Herzegovina	427.4	403.5	453.1	58.6	55.3	62.1	555.2	442.7	702.5	52.2	41.7	66.1
Botswana	45.1	34.5	56.9	47.6	36.4	60.1	80.0	60.5	100.1	35.7	27.0	44.7
Brazil	12027.8	11503.9	12433.7	81.5	78.0	84.3	19226.9	17876.7	20208.8	47.2	43.9	49.6
Brunei Darussalam	22.5	19.7	25.7	150.7	131.9	172.0	33.0	28.5	37.9	63.4	54.7	72.8
Bulgaria	2035.5	1918.6	2151.7	89.9	84.8	95.1	1436.1	1154.9	1780.0	59.0	47.4	73.1
Burkina Faso	540.5	441.9	642.8	73.8	60.3	87.8	892.9	732.4	1070.7	60.9	49.9	73.0
Burundi	206.2	168.3	253.6	54.6	44.6	67.1	257.6	199.5	329.0	34.5	26.7	44.1
Cabo Verde	60.5	54.1	67.4	152.6	136.3	170.0	84.1	73.5	94.9	117.4	102.6	132.4
Cambodia	456.4	358.4	560.0	62.0	48.7	76.1	768.4	610.2	918.1	37.9	30.1	45.3
Cameroon	494.6	409.7	581.6	67.0	55.5	78.8	1065.9	832.8	1361.7	55.9	43.7	71.4
Canada	2942.7	2765.6	3106.3	53.9	50.7	56.9	4866.0	3819.2	6117.1	41.3	32.4	51.9
Central African Republic	129.3	104.7	156.5	67.5	54.7	81.8	162.2	121.4	209.2	47.8	35.8	61.7
Chad	330.6	277.7	390.0	71.4	60.0	84.3	590.0	466.5	735.2	66.3	52.4	82.6
Chile	2722.4	2593.2	2842.3	162.6	154.9	169.7	4202.5	3359.4	5247.5	100.3	80.2	125.2
China	2410054.8	211829.2	272046.5	166.8	146.6	188.3	496398.6	418049.7	585959.5	141.1	118.9	166.6
Colombia	3857.7	3658.7	4028.2	134.5	127.6	140.5	6225.4	4857.2	7865.2	68.5	53.4	86.5
Comoros	15.1	9.8	19.6	41.9	27.1	54.4	23.7	19.4	29.7	30.1	24.7	37.7
Congo	107.1	87.3	128.3	60.4	49.2	72.4	139.4	112.7	171.9	33.9	27.4	41.8
Cook Islands	1.0	0.9	1.1	45.4	39.7	51.2	1.4	1.2	1.7	31.3	26.8	36.9
Costa Rica	538.6	503.5	569.7	189.3	177.0	200.2	964.0	757.7	1216.0	107.7	84.7	135.9
Croatia	1298.1	1214.1	1387.7	112.6	105.3	120.4	979.6	784.2	1208.4	66.6	53.3	82.2
Cuba	656.5	617.2	689.9	38.6	36.3	40.6	1085.3	887.5	1317.4	33.5	27.4	40.7
Cyprus	59.8	51.3	66.9	42.8	36.7	47.9	153.1	133.3	176.4	46.4	40.4	53.4
Czechia	2201.3	2110.4	2288.2	92.9	89.1	96.6	1495.2	1222.3	1808.9	43.3	35.4	52.4
Cote d'Ivoire	455.1	370.8	546.5	71.0	57.8	85.2	927.6	742.3	1151.6	55.2	44.2	68.5
DPRK	3426.5	2698.3	4293.2	129.2	101.8	161.9	5728.3	4651.4	6903.6	106.2	86.3	128.0
Democratic Republic of the Congo	1220.2	984.6	1497.4	45.5	36.7	55.8	1848.5	1425.1	2358.1	32.1	24.8	41.0
Denmark	766.6	726.9	801.4	58.7	55.7	61.4	632.0	495.5	802.2	34.0	26.6	43.1
Djibouti	8.2	6.2	10.9	37.7	28.4	50.0	29.5	22.5	39.6	31.1	23.7	41.7
Dominica	17.3	15.5	19.1	147.5	132.0	162.3	15.3	12.6	18.4	97.8	81.0	117.9
Dominican Republic	185.3	161.2	209.6	30.2	26.3	34.2	597.4	457.4	777.9	38.6	29.6	50.3
Ecuador	1207.9	1125.9	1281.2	139.4	129.9	147.8	2631.2	2091.6	3314.9	102.7	81.7	129.4
Egypt	1121.6	1014.3	1219.9	23.5	21.2	25.6	2241.5	1666.9	2968.3	20.7	15.4	27.4
El Salvador	357.1	327.8	382.9	73.2	67.2	78.5	838.0	644.1	1063.5	85.5	65.7	108.5
Equatorial Guinea	18.7	14.0	23.6	57.6	43.1	72.8	19.8	14.7	27.4	26.5	19.6	36.5
Eritrea	71.1	53.1	92.0	46.7	34.9	60.4	165.7	125.7	210.4	39.6	30.0	50.3
Estonia	467.2	444.7	489.0	130.3	124.0	136.4	337.7	267.2	423.5	77.9	61.7	97.7
Eswatini	20.8	16.6	25.2	45.3	36.2	55.0	33.0	25.5	42.2	35.4	27.3	45.3
Ethiopia	1845.3	1479.3	2288.0	57.5	46.1	71.3	1747.8	1443.4	2170.6	27.0	22.3	33.5

Country	Later-onset gastric cancer (≥ 55 years)											
	1990						2019					
	Incidence number			Incidence rate[a]			Incidence number			Incidence rate[a]		
	Value	LUI	UUI	Value	LUI	UUI	Value	LUI	UUI	Value	LUI	UUI
Fiji	17.7	14.4	21.8	31.4	25.5	38.7	34.4	27.3	43.2	26.2	20.8	33.0
Finland	1003.7	952.0	1052.5	84.6	80.2	88.7	790.9	622.6	983.8	40.2	31.6	50.0
France	8370.8	7861.7	8786.5	60.1	56.4	63.1	8509.2	6660.5	10656.2	39.5	30.9	49.4
Gabon	52.7	42.3	65.7	54.6	43.9	68.0	58.3	45.4	71.9	33.5	26.1	41.3
Gambia	14.9	11.8	18.8	26.8	21.2	33.7	37.1	29.6	45.3	24.7	19.7	30.1
Georgia	928.5	845.7	1013.2	84.5	77.0	92.3	695.6	585.0	827.5	65.8	55.3	78.3
Germany	18431.9	17401.3	19246.2	87.9	82.9	91.7	17328.7	13682.4	21990.4	56.6	44.7	71.8
Ghana	469.8	381.8	567.8	46.0	37.3	55.5	861.7	684.2	1077.1	33.2	26.4	41.5
Greece	2266.4	2139.2	2390.8	84.7	80.0	89.4	2356.5	1871.5	2957.3	64.7	51.4	81.2
Greenland	4.3	3.9	4.9	78.1	69.3	87.8	6.0	4.9	7.2	46.9	38.3	55.8
Grenada	9.8	8.8	10.8	80.8	72.5	89.0	9.2	8.2	10.4	47.3	42.2	53.1
Guam	4.1	3.6	4.7	32.1	28.0	36.6	8.2	6.9	9.8	24.7	20.6	29.5
Guatemala	718.5	640.6	809.9	117.1	104.4	132.0	2215.0	1766.9	2743.8	122.5	97.7	151.8
Guinea	380.5	322.6	447.2	68.8	58.3	80.9	594.5	473.9	733.1	66.8	53.2	82.4
Guinea-Bissau	71.2	55.7	86.9	109.6	85.8	133.7	86.2	68.0	106.8	76.4	60.2	94.6
Guyana	39.5	34.5	45.0	64.5	56.2	73.4	38.2	30.0	47.9	35.2	27.7	44.2
Haiti	516.2	357.0	621.1	95.3	65.9	114.6	727.4	474.0	986.2	63.8	41.6	86.5
Honduras	179.8	148.1	210.5	53.6	44.2	62.7	717.9	592.0	904.8	71.8	59.2	90.5
Hungary	2531.5	2422.5	2637.4	98.3	94.1	102.4	1648.8	1370.6	1965.2	51.5	42.8	61.4
Iceland	41.7	38.1	45.7	87.4	79.8	95.6	35.3	30.3	40.5	37.8	32.5	43.4
India	30049.1	26657.0	33440.7	40.1	35.5	44.6	56456.9	48315.2	65743.7	29.5	25.3	34.4
Indonesia	5598.0	4635.9	6431.8	34.7	28.7	39.8	9776.7	8129.5	11163.7	26.6	22.1	30.4
Iran	4548.4	3907.8	5069.2	98.7	84.8	110.0	9428.6	8674.7	10213.6	77.7	71.5	84.2
Iraq	353.0	285.0	434.4	28.3	22.9	34.9	868.1	681.6	1037.2	23.6	18.5	28.2
Ireland	529.3	498.7	560.0	77.8	73.3	82.3	588.7	459.9	737.5	47.2	36.9	59.2
Israel	462.2	430.7	491.5	57.0	53.1	60.6	769.7	604.1	974.1	40.4	31.7	51.2
Italy	17395.4	16657.6	17980.0	114.3	109.5	118.2	16922.7	13958.1	20019.6	76.7	63.3	90.8
Jamaica	224.9	208.9	241.1	76.4	70.9	81.9	244.7	196.2	301.3	48.2	38.7	59.4
Japan	84126.0	80262.5	86394.5	284.0	270.9	291.6	96068.7	78742.8	113077.9	185.7	152.2	218.5
Jordan	69.0	57.3	81.8	32.1	26.6	38.0	229.1	189.8	276.7	21.5	17.8	25.9
Kazakhstan	3142.6	2979.3	3307.1	150.3	142.5	158.2	2021.1	1734.8	2320.1	66.4	57.0	76.3
Kenya	520.1	418.1	616.4	39.1	31.4	46.3	1306.5	1063.5	1549.5	36.9	30.1	43.8
Kiribati	4.1	3.3	4.9	68.8	55.8	83.0	6.3	4.9	8.0	54.3	42.0	68.7
Kuwait	24.3	21.6	27.7	26.4	23.4	30.1	64.0	52.3	77.6	16.1	13.2	19.6
Kyrgyzstan	686.9	645.9	727.7	131.1	123.3	138.9	570.1	491.7	649.6	72.2	62.3	82.3
Lao People's Democratic Republic	227.9	173.6	285.0	66.4	50.6	83.1	233.3	177.7	289.1	32.3	24.6	40.0
Latvia	741.7	711.3	773.4	118.5	113.7	123.6	466.1	390.5	559.6	70.3	58.9	84.4
Lebanon	167.8	139.5	203.0	42.4	35.2	51.2	344.0	278.4	449.3	39.7	32.1	51.9
Lesotho	74.1	58.8	91.4	44.6	35.4	55.0	100.8	76.5	129.2	47.8	36.3	61.3
Liberia	130.3	108.8	154.3	70.4	58.8	83.4	152.3	116.4	195.5	48.7	37.2	62.5
Libya	95.4	71.4	120.5	31.2	23.3	39.4	200.8	155.0	253.7	25.4	19.6	32.1
Lithuania	934.8	894.1	973.0	117.8	112.7	122.6	667.0	544.1	806.6	69.2	56.4	83.7
Luxembourg	65.2	60.4	70.7	69.8	64.6	75.7	59.4	48.2	72.1	35.9	29.1	43.5
Madagascar	302.8	257.8	351.7	35.5	30.2	41.2	457.7	344.7	594.4	26.1	19.7	33.9
Malawi	124.2	105.4	144.1	19.7	16.7	22.8	163.8	134.7	195.1	14.3	11.8	17.1
Malaysia	652.8	594.6	718.7	44.6	40.7	49.2	1477.9	1189.3	1831.0	31.9	25.7	39.6
Maldives	5.4	4.3	6.3	35.2	27.9	41.4	8.1	6.6	9.7	16.7	13.6	20.0
Mali	639.4	535.4	755.4	92.3	77.3	109.1	1031.8	841.7	1283.3	73.9	60.3	92.0
Malta	48.4	43.9	52.6	66.7	60.4	72.4	57.7	48.5	67.8	37.5	31.5	44.1
Marshall Islands	2.1	1.7	2.5	82.0	67.1	96.6	3.2	2.4	4.1	55.9	42.4	71.7
Mauritania	127.2	106.0	149.9	76.5	63.7	90.1	162.5	124.9	204.0	47.4	36.4	59.4
Mauritius	82.8	77.7	88.4	67.5	63.3	72.1	109.3	87.9	133.1	34.3	27.6	41.7
Mexico	4318.0	4098.9	4457.2	61.9	58.8	63.9	8468.1	7380.3	9742.4	43.0	37.4	49.4
Micronesia	5.8	4.6	7.2	76.2	60.3	93.8	7.2	5.4	9.2	58.6	44.0	74.3
Monaco	9.4	7.4	11.2	84.3	66.5	101.2	8.6	6.9	10.2	56.6	45.3	67.1
Mongolia	520.1	434.7	618.6	300.2	251.0	357.1	677.8	527.5	867.8	175.9	136.9	225.2
Montenegro	45.2	39.7	50.7	41.5	36.5	46.6	71.3	59.3	85.0	40.9	34.0	48.7
Morocco	546.9	412.8	629.6	24.2	18.2	27.8	1111.8	844.2	1320.1	20.4	15.5	24.2
Mozambique	292.3	240.6	345.2	29.8	24.5	35.2	536.6	428.9	660.3	30.9	24.7	38.1
Myanmar	2262.5	1768.2	2968.6	57.7	45.1	75.8	2302.8	1940.6	2794.0	29.1	24.5	35.3
Namibia	22.1	18.0	25.9	18.4	15.0	21.6	35.8	29.1	43.8	15.8	12.8	19.3
Nauru	0.4	0.3	0.5	68.8	54.0	85.8	0.3	0.3	0.4	53.2	41.8	66.5
Nepal	587.3	453.9	739.7	38.2	29.5	48.1	1380.6	1074.0	1748.6	36.6	28.5	46.3

Country	Later-onset gastric cancer (≥ 55 years)											
	1990						2019					
	Incidence number			Incidence rate[a]			Incidence number			Incidence rate[a]		
	Value	LUI	UUI	Value	LUI	UUI	Value	LUI	UUI	Value	LUI	UUI
Netherlands	2696.1	2539.4	2830.7	81.4	76.6	85.4	3231.3	2548.2	4007.6	57.2	45.1	71.0
New Zealand	361.6	336.8	385.3	54.9	51.2	58.6	465.9	383.6	554.6	35.3	29.1	42.0
Nicaragua	177.4	159.1	194.9	71.6	64.2	78.6	540.1	443.0	644.9	73.1	60.0	87.3
Niger	301.5	242.3	355.9	68.5	55.1	80.9	712.7	541.4	895.7	55.0	41.8	69.1
Nigeria	1463.9	1158.4	1819.7	20.3	16.1	25.2	2326.6	1889.2	2854.5	16.7	13.5	20.4
Niue	0.2	0.2	0.3	62.9	53.5	74.7	0.2	0.2	0.2	48.3	40.8	56.0
North Macedonia	335.3	306.6	359.6	102.2	93.4	109.6	447.3	354.2	561.8	78.2	61.9	98.2
Northern Mariana Islands	1.6	1.4	1.9	65.2	55.7	77.2	4.5	3.8	5.2	44.9	37.9	51.5
Norway	809.7	763.3	847.7	74.8	70.5	78.3	582.4	493.7	682.9	37.4	31.7	43.9
Oman	58.0	43.2	73.7	58.0	43.2	73.7	87.4	76.0	100.6	31.7	27.6	36.6
Pakistan	2696.5	2114.9	3207.6	29.4	23.0	34.9	4664.4	3836.6	5703.3	26.2	21.6	32.1
Palau	0.9	0.7	1.1	54.3	43.7	68.3	1.6	1.3	2.0	41.4	33.0	51.9
Palestine	68.9	52.5	88.1	47.4	36.2	60.6	113.1	96.5	131.2	29.0	24.8	33.7
Panama	202.1	187.7	215.7	83.1	77.2	88.7	404.6	315.4	512.0	58.1	45.3	73.5
Papua New Guinea	154.3	115.1	202.3	51.0	38.0	66.9	369.2	265.1	491.9	49.5	35.6	66.0
Paraguay	171.7	151.1	191.9	47.3	41.6	52.9	381.9	296.0	486.2	40.6	31.5	51.7
Peru	2270.1	1959.1	2625.9	115.3	99.5	133.4	5142.0	3882.5	6630.5	96.5	72.9	124.5
Philippines	1332.1	1176.6	1506.5	27.8	24.6	31.5	2356.2	1946.9	2835.8	18.0	14.8	21.6
Poland	6887.5	6643.1	7076.0	89.4	86.2	91.8	5690.4	4830.1	6729.5	47.2	40.0	55.8
Portugal	3241.6	3092.7	3375.0	132.8	126.7	138.3	2857.0	2253.1	3581.9	75.4	59.5	94.5
Puerto Rico	364.2	338.3	385.9	59.9	55.6	63.5	381.3	298.8	487.0	32.4	25.4	41.4
Qatar	7.2	5.7	8.8	44.3	35.2	54.7	34.1	25.3	45.4	19.7	14.7	26.3
Republic of Korea	12864.6	12236.7	13586.0	258.3	245.6	272.7	20722.2	17007.0	24730.1	129.2	106.1	154.2
Republic of Moldova	658.5	627.8	688.7	85.1	81.2	89.0	481.1	413.1	556.2	46.2	39.7	53.5
Romania	3408.8	3254.7	3561.3	68.1	65.0	71.1	3651.5	2998.4	4424.2	59.5	48.8	72.0
Russian Federation	44792.7	43440.5	45603.1	142.0	137.7	144.6	30691.3	27058.2	34919.1	72.3	63.7	82.2
Rwanda	262.4	215.5	314.3	54.9	45.1	65.8	285.8	225.6	354.3	28.5	22.5	35.3
Saint Kitts and Nevis	6.0	5.3	6.6	93.6	83.9	103.1	5.6	4.8	6.5	46.5	39.7	54.1
Saint Lucia	13.9	12.7	15.0	94.7	86.5	102.6	20.7	17.4	24.2	56.4	47.3	65.9
Saint Vincent and the Grenadines	9.3	8.4	10.3	77.4	69.8	85.5	11.9	10.3	13.7	49.9	43.5	57.7
Samoa	10.1	8.4	12.2	68.9	57.2	82.6	12.9	10.6	15.4	52.4	43.1	62.9
San Marino	10.4	8.9	11.9	183.9	156.5	210.4	15.8	12.2	20.4	156.4	121.1	202.1
Sao Tome and Principe	8.5	7.1	9.9	75.5	63.3	87.9	12.6	10.2	15.7	74.2	60.3	92.4
Saudi Arabia	256.3	196.4	317.9	28.7	22.0	35.6	425.2	347.7	516.6	15.6	12.8	19.0
Senegal	377.2	302.5	445.4	71.4	57.3	84.3	707.8	557.2	873.0	57.1	44.9	70.4
Serbia	1230.8	1043.0	1366.1	59.2	50.2	65.7	1308.2	1049.5	1628.0	47.8	38.3	59.5
Seychelles	5.0	4.5	5.7	52.0	46.5	59.5	5.8	5.0	6.7	30.4	26.2	34.9
Sierra Leone	222.5	180.1	266.2	71.8	58.1	85.9	318.3	246.7	406.0	56.2	43.5	71.6
Singapore	460.9	433.2	489.1	127.1	119.5	134.9	701.2	562.8	874.2	50.4	40.5	62.9
Slovakia	1062.6	1010.3	1118.9	102.4	97.4	107.9	987.5	779.7	1216.5	61.5	48.5	75.7
Slovenia	445.7	348.3	556.7	104.0	81.3	129.9	452.8	357.4	584.2	63.4	50.1	81.8
Solomon Islands	19.8	14.0	25.4	88.3	62.4	113.7	38.3	29.9	47.7	81.3	63.4	101.3
Somalia	203.3	151.5	259.3	52.9	39.4	67.5	422.0	309.3	561.4	41.5	30.4	55.1
South Africa	1110.6	976.6	1247.2	32.6	28.6	36.6	1819.6	1690.9	1960.0	24.1	22.4	25.9
South Sudan	162.6	121.7	208.8	42.8	32.0	54.9	173.6	127.2	233.6	28.5	20.9	38.3
Spain	8969.0	8384.5	9420.1	93.8	87.7	98.5	9669.2	7594.2	12107.2	64.5	50.7	80.8
Sri Lanka	687.9	615.5	771.9	38.5	34.5	43.2	1117.9	843.3	1473.9	24.5	18.5	32.3
Sudan	1105.4	750.3	1348.8	74.3	50.4	90.7	1934.8	1319.0	2485.5	66.5	45.3	85.4
Suriname	19.3	17.4	21.0	43.7	39.4	47.7	33.0	27.5	39.4	31.7	26.5	37.9
Sweden	1572.1	1470.6	1654.4	66.2	61.9	69.7	973.7	816.5	1131.7	29.8	25.0	34.7
Switzerland	1309.9	1225.0	1391.0	77.5	72.5	82.3	1032.7	801.3	1287.0	36.9	28.6	46.0
Syrian Arab Republic	201.0	159.3	247.9	22.7	18.0	28.0	426.0	319.6	565.7	19.8	14.8	26.2
Tajikistan	640.7	591.8	694.2	133.5	123.3	144.6	794.4	643.2	971.0	90.7	73.4	110.9
Thailand	2619.4	2292.1	2976.0	44.2	38.6	50.2	4430.8	3318.6	5792.5	24.4	18.3	32.0
Timor-Leste	17.3	13.0	21.7	39.7	29.8	49.8	45.4	33.5	55.9	33.3	24.6	41.1
Togo	135.1	112.0	160.4	69.0	57.2	82.0	318.0	253.4	404.5	54.4	43.4	69.3
Tokelau	0.1	0.1	0.2	59.9	49.2	72.6	0.1	0.1	0.1	40.8	33.2	50.3
Tonga	6.0	4.9	7.0	62.6	51.5	73.9	7.8	6.5	9.1	59.5	49.6	70.0
Trinidad and Tobago	76.9	70.9	83.0	56.0	51.6	60.4	70.2	53.8	90.6	21.0	16.1	27.1
Tunisia	253.7	208.7	305.4	29.1	23.9	35.0	560.4	416.7	755.1	25.4	18.9	34.3
Turkey	5060.5	4313.1	5871.7	82.4	70.2	95.6	8149.2	6510.7	9968.8	53.8	43.0	65.8
Turkmenistan	317.5	301.0	334.7	97.6	92.6	102.9	275.5	217.2	346.2	40.8	32.2	51.2
Tuvalu	0.8	0.7	1.0	70.2	56.8	85.8	0.9	0.7	1.2	51.2	40.1	65.3

Country	Later-onset gastric cancer (≥ 55 years)											
	1990						2019					
	Incidence number			Incidence rate[a]			Incidence number			Incidence rate[a]		
	Value	LUI	UUI	Value	LUI	UUI	Value	LUI	UUI	Value	LUI	UUI
Uganda	451.9	373.5	528.6	43.1	35.6	50.4	763.2	620.6	917.3	34.3	27.9	41.3
Ukraine	16423.0	15681.0	17139.3	131.8	125.8	137.5	9580.0	8064.0	11197.8	71.1	59.9	83.1
United Arab Emirates	28.9	22.3	35.9	54.5	42.1	67.8	143.6	106.4	188.0	26.5	19.6	34.7
United Kingdom	11499.7	11046.5	11795.3	77.1	74.0	79.1	8693.4	7290.8	10291.6	43.0	36.1	50.9
United Republic of Tanzania	762.7	638.8	898.1	42.2	35.3	49.7	1226.7	1000.9	1444.3	31.5	25.7	37.0
United States of America	23580.1	22444.6	24262.0	44.8	42.6	46.1	28054.5	24150.0	32528.5	29.1	25.0	33.7
United States Virgin Islands	6.9	5.8	8.1	49.2	41.5	57.6	16.7	14.0	19.3	50.5	42.5	58.4
Uruguay	589.7	556.8	622.1	87.4	82.5	92.2	537.4	425.3	665.6	60.4	47.8	74.8
Uzbekistan	1819.8	1716.4	1916.3	94.4	89.0	99.4	1982.8	1648.0	2316.7	53.5	44.5	62.5
Vanuatu	6.4	4.7	8.3	62.1	45.2	80.0	16.2	11.8	21.1	57.4	41.7	74.7
Venezuela	1428.8	1337.9	1507.6	91.2	85.4	96.2	3189.8	2444.9	4080.7	63.3	48.5	81.0
Viet Nam	5674.1	4531.4	6821.3	81.3	64.9	97.8	7301.4	5886.8	8772.4	45.2	36.5	54.3
Yemen	695.1	503.5	901.3	86.8	62.9	112.6	1773.7	1363.1	2329.3	84.1	64.6	110.4
Zambia	216.5	180.2	255.2	47.0	39.1	55.4	350.4	279.0	428.8	34.3	27.3	42.0
Zimbabwe	363.1	316.8	410.3	54.8	47.8	61.9	611.0	485.5	759.6	54.8	43.6	68.2

LUI, lower uncertainty intervals; UUI, upper uncertainty intervals; [a]per 100 000.

Table S5. Deaths due to early-onset gastric cancer in 203 countries, 2019 versus 1990

Country	Early-onset gastric cancer (15–49 years)											
	1990						2019					
	Death number			Death rate (per 100 000)			Death number			Death rate (per 100 000)		
	Value	LUI	UUI	Value	LUI	UUI	Value	LUI	UUI	Value	LUI	UUI
Afghanistan	347.6	174.1	498.9	7.3	3.7	10.5	1067.6	591.8	1576.4	5.9	3.3	8.7
Albania	47.9	43.5	52.2	2.8	2.5	3.1	32.8	23.9	43.9	2.5	1.8	3.4
Algeria	147.2	113.7	187.1	1.2	1.0	1.6	190.4	143.1	248.9	0.8	0.6	1.1
American Samoa	1.0	0.8	1.2	4.0	3.3	4.9	1.1	0.8	1.5	4.0	2.9	5.5
Andorra	0.6	0.4	0.8	1.8	1.3	2.4	0.7	0.5	0.9	1.7	1.2	2.3
Angola	115.5	81.1	157.8	2.5	1.7	3.4	181.7	125.1	248.5	1.3	0.9	1.8
Antigua and Barbuda	0.7	0.6	0.8	2.2	1.9	2.5	0.7	0.6	0.9	1.5	1.2	1.8
Argentina	385.4	362.3	409.7	2.4	2.3	2.6	402.8	359.2	452.8	1.7	1.5	2.0
Armenia	87.1	80.8	93.6	5.1	4.7	5.5	40.1	32.3	48.8	2.7	2.2	3.3
Australia	88.9	83.7	94.7	1.0	0.9	1.1	82.3	70.9	95.6	0.7	0.6	0.8
Austria	113.2	105.9	121.0	2.8	2.6	3.0	34.0	29.6	38.7	0.8	0.7	1.0
Azerbaijan	211.4	191.1	233.1	5.8	5.2	6.3	204.8	162.2	255.8	3.7	2.9	4.6
Bahamas	3.5	3.1	4.0	2.5	2.2	2.8	4.6	3.6	5.9	2.3	1.7	2.9
Bahrain	3.3	2.7	3.9	1.1	0.9	1.3	6.9	5.3	9.0	0.7	0.6	1.0
Bangladesh	1021.4	737.5	1247.6	2.0	1.5	2.5	1106.4	786.5	1638.2	1.3	0.9	1.9
Barbados	3.4	3.0	3.8	2.5	2.2	2.8	2.8	2.1	3.6	2.0	1.5	2.5
Belarus	463.4	435.4	491.1	9.2	8.6	9.7	165.1	123.4	221.7	3.8	2.8	5.1
Belgium	70.0	64.1	76.0	1.4	1.3	1.5	38.2	32.6	44.3	0.8	0.7	0.9
Belize	1.2	1.0	1.3	1.4	1.2	1.5	4.4	3.6	5.2	1.9	1.6	2.3
Benin	38.0	30.3	47.3	1.9	1.5	2.4	78.2	53.2	110.8	1.3	0.9	1.9
Bermuda	0.6	0.5	0.7	1.7	1.5	2.0	0.2	0.2	0.3	0.9	0.7	1.1
Bhutan	5.6	3.4	7.9	1.8	1.1	2.6	6.2	3.8	9.4	1.4	0.9	2.1
Bolivia	198.0	150.2	244.4	6.6	5.0	8.2	256.2	166.3	360.2	4.2	2.7	5.9
Bosnia and Herzegovina	61.4	56.3	67.6	2.5	2.3	2.8	35.2	26.1	47.0	2.3	1.7	3.1
Botswana	9.5	6.3	14.0	1.6	1.0	2.3	20.4	12.4	31.1	1.5	0.9	2.3
Brazil	2121.6	2047.3	2198.6	2.8	2.7	2.9	2353.5	2232.5	2467.4	2.0	1.9	2.1
Brunei Darussalam	7.6	6.3	9.0	5.2	4.3	6.1	6.8	5.7	8.2	2.6	2.1	3.1
Bulgaria	182.9	167.8	199.0	4.4	4.0	4.8	90.4	68.5	119.4	3.0	2.3	3.9
Burkina Faso	80.5	60.8	105.9	2.1	1.6	2.8	170.4	122.4	222.8	1.7	1.2	2.2
Burundi	61.5	45.1	79.5	2.5	1.9	3.3	85.0	60.1	116.7	1.6	1.1	2.1
Cabo Verde	5.3	4.5	6.3	3.6	3.1	4.3	6.8	5.0	9.1	2.2	1.6	3.0
Cambodia	133.3	92.7	171.7	2.9	2.0	3.7	136.5	100.6	184.6	1.6	1.1	2.1
Cameroon	91.7	71.0	112.8	2.0	1.6	2.5	212.9	138.0	311.1	1.5	1.0	2.2
Canada	153.2	144.5	162.4	1.0	1.0	1.1	118.3	101.0	136.7	0.7	0.6	0.8
Central African Republic	39.1	29.3	49.4	3.1	2.3	4.0	62.6	42.7	89.8	2.4	1.7	3.5
Chad	47.7	37.4	59.5	1.9	1.5	2.4	106.4	75.8	144.7	1.6	1.1	2.1
Chile	265.1	248.6	284.9	3.7	3.5	4.0	209.5	186.9	235.2	2.3	2.0	2.6
China	39449.1	33804.0	45353.5	5.9	5.1	6.8	31113.9	25687.5	37122.8	4.3	3.6	5.2
Colombia	637.5	601.7	673.9	3.8	3.6	4.0	712.8	516.3	941.4	2.9	2.1	3.8
Comoros	3.2	0.9	4.9	1.6	0.4	2.4	5.1	3.1	7.1	1.4	0.8	1.9
Congo	28.6	20.6	37.8	2.6	1.9	3.4	41.2	27.3	59.2	1.5	1.0	2.2
Cook Islands	0.2	0.2	0.3	2.4	1.8	3.2	0.2	0.1	0.2	1.9	1.2	2.7
Costa Rica	70.9	65.7	76.4	4.6	4.3	5.0	83.5	62.3	110.6	3.3	2.5	4.4
Croatia	92.9	84.1	102.5	3.8	3.4	4.2	29.4	21.6	38.8	1.6	1.2	2.1
Cuba	75.4	69.6	81.9	1.2	1.1	1.3	53.5	41.3	67.9	1.0	0.8	1.3
Cyprus	3.8	3.2	4.5	0.9	0.8	1.1	5.8	4.8	7.0	0.8	0.7	1.0
Czechia	161.7	150.2	173.6	3.1	2.9	3.3	58.9	44.3	76.3	1.2	0.9	1.6
Cote d'Ivoire	119.3	89.4	152.5	2.1	1.6	2.7	206.8	140.5	285.2	1.6	1.1	2.2
Democratic People's Republic of Korea	858.7	571.6	1208.1	8.0	5.4	11.3	813.2	526.5	1214.3	5.8	3.8	8.7
Democratic Republic of the Congo	283.3	209.4	369.7	1.7	1.2	2.2	503.5	363.5	682.6	1.2	0.9	1.6
Denmark	50.2	45.9	55.1	1.9	1.7	2.1	16.4	13.8	19.5	0.6	0.5	0.8
Djibouti	3.4	2.3	4.8	1.4	1.0	2.1	9.7	5.6	15.7	1.5	0.9	2.4
Dominica	1.5	1.2	1.8	4.2	3.4	5.1	1.2	0.9	1.5	3.5	2.6	4.5
Dominican Republic	53.0	45.3	60.8	1.5	1.2	1.7	110.9	77.4	156.6	1.9	1.3	2.7
Ecuador	211.9	196.2	227.0	4.3	4.0	4.6	330.5	245.8	436.9	3.6	2.7	4.8
Egypt	253.3	221.7	289.8	0.9	0.8	1.1	351.7	251.1	480.6	0.7	0.5	0.9
El Salvador	77.1	71.0	83.5	3.1	2.8	3.3	98.5	72.2	134.6	3.0	2.2	4.1
Equatorial Guinea	4.7	3.3	6.4	2.6	1.8	3.5	5.0	2.9	8.4	0.7	0.4	1.1
Eritrea	33.9	24.8	43.4	2.5	1.8	3.2	71.4	47.8	101.8	2.1	1.4	2.9
Estonia	51.3	47.2	55.4	6.8	6.2	7.3	16.0	11.6	21.4	2.8	2.0	3.7
Eswatini	4.9	3.6	6.5	1.4	1.0	1.8	9.4	5.7	14.2	1.6	0.9	2.3
Ethiopia	739.2	448.0	925.8	3.3	2.0	4.2	534.0	399.5	739.7	1.0	0.8	1.4

Country	Early-onset gastric cancer (15–49 years)											
	1990						2019					
	Death number			Death rate (per 100 000)			Death number			Death rate (per 100 000)		
	Value	LUI	UUI	Value	LUI	UUI	Value	LUI	UUI	Value	LUI	UUI
Fiji	7.9	6.2	10.0	2.0	1.6	2.5	8.0	5.8	10.6	1.7	1.3	2.3
Finland	67.1	60.8	73.1	2.6	2.4	2.8	19.1	16.2	22.5	0.8	0.7	1.0
France	407.8	382.8	434.0	1.4	1.3	1.5	265.7	224.4	309.6	0.9	0.8	1.1
Gabon	9.1	6.8	11.8	2.0	1.5	2.6	11.2	7.6	16.0	1.2	0.8	1.7
Gambia	2.6	1.8	3.6	0.6	0.4	0.8	5.6	3.9	7.8	0.5	0.4	0.7
Georgia	146.3	129.1	164.7	5.5	4.8	6.1	66.1	51.9	82.0	4.0	3.1	5.0
Germany	915.4	872.8	960.2	2.3	2.2	2.4	447.3	388.8	508.8	1.2	1.1	1.4
Ghana	110.2	81.9	143.4	1.6	1.2	2.1	163.2	111.7	222.5	1.0	0.7	1.3
Greece	111.7	103.8	120.8	2.2	2.1	2.4	75.2	66.1	84.5	1.7	1.5	1.9
Greenland	1.3	1.0	1.7	4.0	2.9	5.0	0.6	0.4	0.8	2.1	1.6	2.9
Grenada	0.9	0.8	1.1	2.4	2.1	2.8	0.9	0.7	1.1	1.6	1.3	2.0
Guam	1.0	0.9	1.2	1.3	1.1	1.6	1.5	1.2	1.9	1.9	1.5	2.4
Guatemala	196.1	168.0	223.6	5.7	4.8	6.4	457.1	345.6	585.2	4.8	3.6	6.1
Guinea	70.0	55.5	85.8	2.7	2.1	3.3	131.5	94.4	178.8	2.3	1.6	3.1
Guinea-Bissau	16.0	11.8	21.3	3.6	2.7	4.8	22.4	16.2	30.2	2.4	1.7	3.2
Guyana	8.7	7.2	10.5	2.2	1.8	2.6	8.3	5.9	11.2	2.0	1.4	2.7
Haiti	128.7	76.9	162.7	4.4	2.6	5.6	185.2	109.7	261.6	2.8	1.7	4.0
Honduras	67.2	51.8	81.2	3.3	2.5	4.0	104.9	63.1	161.8	2.0	1.2	3.1
Hungary	193.8	178.1	210.6	3.8	3.5	4.1	72.1	55.7	92.5	1.6	1.3	2.1
Iceland	2.2	2.0	2.5	1.7	1.5	1.9	1.2	1.0	1.4	0.7	0.6	0.9
India	11056.6	9720.0	12299.5	2.6	2.3	2.9	15428.8	12981.1	18057.1	2.0	1.7	2.4
Indonesia	1681.8	1297.9	1921.6	1.8	1.4	2.0	1657.5	1326.4	2098.2	1.2	0.9	1.5
Iran	756.5	667.7	843.7	2.9	2.5	3.2	1089.8	1021.7	1175.9	2.3	2.2	2.5
Iraq	106.7	81.1	139.1	1.4	1.0	1.8	275.1	196.3	387.7	1.2	0.8	1.7
Ireland	25.2	23.1	27.6	1.4	1.3	1.5	18.5	15.9	21.1	0.8	0.7	0.9
Israel	34.6	31.3	38.0	1.4	1.3	1.6	44.5	39.1	50.0	1.0	0.9	1.1
Italy	774.3	755.8	793.2	2.7	2.6	2.8	389.5	366.8	417.5	1.5	1.4	1.6
Jamaica	12.7	11.0	14.6	1.1	0.9	1.3	21.0	15.3	28.3	1.4	1.0	1.8
Japan	4643.8	4556.5	4730.3	7.1	7.0	7.3	1173.1	1108.2	1224.7	2.3	2.1	2.4
Jordan	19.2	15.3	23.5	1.1	0.9	1.3	53.4	42.0	65.9	0.8	0.7	1.0
Kazakhstan	559.1	523.0	598.6	6.8	6.4	7.3	262.0	215.4	316.3	2.8	2.3	3.4
Kenya	118.3	91.3	163.4	1.2	0.9	1.6	367.2	283.4	480.0	1.4	1.1	1.8
Kiribati	2.3	1.8	2.8	6.1	4.8	7.7	3.4	2.6	4.5	5.7	4.2	7.4
Kuwait	6.2	5.3	7.0	0.6	0.5	0.7	11.6	9.3	14.5	0.4	0.3	0.5
Kyrgyzstan	159.6	147.5	173.5	7.6	7.0	8.3	123.9	103.0	148.1	3.7	3.1	4.4
Lao People's Democratic Republic	68.2	46.9	89.6	3.7	2.5	4.8	58.1	38.5	77.8	1.5	1.0	2.0
Latvia	94.4	87.7	101.1	7.3	6.8	7.9	25.2	19.8	31.9	3.1	2.4	3.9
Lebanon	26.2	20.1	32.8	1.7	1.3	2.2	32.8	24.2	43.5	1.2	0.9	1.6
Lesotho	11.0	8.0	14.3	1.3	1.0	1.7	24.6	16.3	34.4	2.1	1.4	3.0
Liberia	16.6	12.5	21.4	2.0	1.5	2.6	34.3	22.8	47.6	1.4	0.9	1.9
Libya	20.8	15.5	27.1	1.1	0.8	1.4	48.1	34.1	66.3	1.2	0.8	1.6
Lithuania	116.6	108.3	125.0	6.4	5.9	6.8	37.6	28.5	48.4	3.1	2.4	4.0
Luxembourg	4.2	3.8	4.7	2.1	1.9	2.4	2.3	1.9	2.7	0.7	0.6	0.9
Madagascar	107.2	88.2	126.3	2.0	1.7	2.4	179.7	125.5	238.9	1.4	1.0	1.8
Malawi	28.1	22.2	35.2	0.7	0.5	0.8	38.1	26.6	52.6	0.4	0.3	0.6
Malaysia	127.8	110.9	146.1	1.4	1.2	1.6	167.8	127.7	220.2	1.0	0.7	1.3
Maldives	1.4	0.9	1.7	1.5	1.0	1.8	1.3	1.0	1.6	0.4	0.3	0.5
Mali	157.6	126.6	190.4	4.4	3.5	5.3	257.0	180.1	350.2	2.7	1.9	3.6
Malta	2.8	2.6	3.2	1.5	1.3	1.7	1.7	1.4	2.0	0.8	0.7	1.0
Marshall Islands	0.9	0.8	1.1	4.6	3.7	5.5	1.7	1.2	2.2	5.5	3.9	7.4
Mauritania	18.6	14.5	23.5	2.0	1.6	2.6	17.8	11.0	26.8	0.9	0.6	1.4
Mauritius	12.6	11.3	14.0	2.1	1.9	2.3	9.1	7.0	11.9	1.4	1.1	1.8
Mexico	777.5	755.5	800.0	1.8	1.8	1.9	1333.7	1126.4	1563.7	2.0	1.7	2.4
Micronesia	2.6	1.9	3.5	5.6	4.1	7.3	2.7	0.8	3.9	5.0	1.5	7.3
Monaco	0.3	0.3	0.4	2.4	1.9	3.0	0.2	0.1	0.3	1.3	1.0	1.9
Mongolia	85.5	67.6	107.1	8.3	6.6	10.4	168.8	126.2	226.9	9.3	7.0	12.6
Montenegro	5.2	4.6	6.0	1.6	1.4	1.9	3.7	3.0	4.6	1.3	1.0	1.6
Morocco	98.3	78.5	121.5	0.8	0.6	1.0	126.6	88.2	192.7	0.7	0.5	1.0
Mozambique	43.5	32.7	55.6	0.8	0.6	1.0	98.0	65.0	140.7	0.7	0.5	1.1
Myanmar	685.8	494.8	910.8	3.4	2.5	4.5	431.8	308.6	581.8	1.5	1.1	2.0
Namibia	3.6	2.4	4.8	0.6	0.4	0.7	6.2	3.9	9.1	0.5	0.3	0.7
Nauru	0.3	0.2	0.4	6.1	4.5	8.2	0.3	0.2	0.4	5.4	4.0	7.2
Nepal	217.6	156.6	285.3	2.4	1.8	3.2	268.1	184.8	375.4	1.7	1.1	2.3

Country	Early-onset gastric cancer (15–49 years)											
	1990						2019					
	Death number			Death rate (per 100 000)			Death number			Death rate (per 100 000)		
	Value	LUI	UUI	Value	LUI	UUI	Value	LUI	UUI	Value	LUI	UUI
Netherlands	131.0	121.8	140.7	1.6	1.5	1.7	70.6	60.3	82.3	0.9	0.8	1.1
New Zealand	29.4	26.6	32.6	1.6	1.5	1.8	19.8	17.6	22.2	1.0	0.9	1.1
Nicaragua	39.2	34.7	44.1	2.3	2.0	2.6	58.0	44.2	75.1	1.7	1.3	2.1
Niger	69.7	53.9	88.2	2.1	1.6	2.6	120.8	82.7	168.7	1.3	0.9	1.8
Nigeria	209.2	153.3	280.8	0.5	0.4	0.7	363.7	260.6	507.0	0.4	0.3	0.5
Niue	0.0	0.0	0.1	3.7	2.4	5.0	0.0	0.0	0.0	2.7	1.6	4.1
North Macedonia	47.3	42.0	52.4	4.5	4.0	5.0	32.0	24.0	41.7	3.0	2.2	3.9
Northern Mariana Islands	1.3	0.9	1.7	4.4	3.0	5.8	0.7	0.5	0.9	3.2	2.3	4.3
Norway	35.1	33.9	36.5	1.6	1.6	1.7	15.4	14.0	16.7	0.6	0.6	0.7
Oman	13.9	10.0	18.8	1.4	1.0	2.0	18.2	14.9	22.2	0.6	0.5	0.7
Pakistan	614.1	517.3	717.7	1.2	1.0	1.4	1504.7	1190.0	1885.4	1.3	1.1	1.7
Palau	0.4	0.2	0.5	4.2	2.8	5.6	0.5	0.3	0.7	5.5	3.7	7.4
Palestine	16.6	12.0	22.3	1.9	1.4	2.5	28.8	23.6	34.5	1.1	0.9	1.4
Panama	35.1	31.6	39.0	2.9	2.6	3.2	47.9	34.2	64.5	2.3	1.6	3.1
Papua New Guinea	66.5	46.7	90.4	3.3	2.4	4.6	179.3	120.3	257.7	3.5	2.4	5.1
Paraguay	32.5	28.1	37.6	1.7	1.5	2.0	55.4	40.6	74.1	1.5	1.1	2.0
Peru	447.4	380.8	521.0	4.2	3.6	4.9	598.7	423.2	833.4	3.4	2.4	4.7
Philippines	493.7	439.8	549.4	1.6	1.4	1.8	586.7	472.6	707.7	1.0	0.8	1.2
Poland	690.9	668.4	712.9	3.6	3.5	3.8	364.8	297.8	440.8	2.0	1.6	2.4
Portugal	277.1	262.0	293.2	5.5	5.2	5.9	133.4	118.3	151.4	2.8	2.5	3.2
Puerto Rico	28.5	25.6	31.6	1.5	1.4	1.7	13.3	9.8	17.9	0.8	0.6	1.1
Qatar	2.8	2.1	3.6	1.0	0.7	1.2	9.9	7.4	13.2	0.5	0.3	0.6
Republic of Korea	3019.6	2801.7	3178.6	11.7	10.8	12.3	867.5	765.2	984.3	3.3	2.9	3.8
Republic of Moldova	132.7	123.8	141.7	6.0	5.6	6.4	52.1	42.7	62.3	2.8	2.3	3.4
Romania	374.0	348.5	401.8	3.3	3.1	3.5	237.5	183.1	304.6	2.7	2.1	3.5
Russian Federation	4523.5	4128.7	4706.4	6.1	5.6	6.3	2311.2	1944.7	2712.4	3.4	2.8	4.0
Rwanda	80.8	61.1	99.4	2.6	1.9	3.2	76.9	51.7	106.6	1.2	0.8	1.6
Saint Kitts and Nevis	0.5	0.5	0.6	2.7	2.3	3.0	0.4	0.1	0.7	1.3	0.5	2.1
Saint Lucia	2.0	1.8	2.3	3.0	2.7	3.5	2.5	2.0	3.1	2.6	2.1	3.3
Saint Vincent and the Grenadines	1.3	1.1	1.5	2.4	2.1	2.8	1.4	1.2	1.7	2.5	2.0	3.0
Samoa	1.8	1.2	2.5	2.3	1.5	3.2	2.4	1.5	3.5	2.3	1.4	3.4
San Marino	0.4	0.3	0.5	3.4	2.6	4.3	0.4	0.2	0.6	2.7	1.6	4.3
Sao Tome and Principe	0.7	0.5	1.0	1.5	0.9	1.9	1.7	1.2	2.4	1.6	1.1	2.2
Saudi Arabia	64.6	42.7	91.0	0.8	0.5	1.1	171.8	122.4	241.6	0.7	0.5	1.0
Senegal	61.2	46.6	75.7	1.9	1.4	2.3	95.6	64.7	130.5	1.3	0.9	1.8
Serbia	144.7	124.3	166.2	3.2	2.7	3.6	72.2	53.5	95.2	1.8	1.3	2.4
Seychelles	0.8	0.7	0.9	2.1	1.8	2.4	0.9	0.8	1.1	1.8	1.5	2.1
Sierra Leone	29.8	21.1	40.6	1.8	1.3	2.5	59.3	41.3	82.9	1.4	1.0	2.0
Singapore	44.7	40.6	49.1	2.4	2.1	2.6	21.7	18.6	25.2	0.7	0.6	0.8
Slovakia	89.9	81.8	99.1	3.4	3.1	3.7	44.1	32.6	58.7	1.7	1.2	2.2
Slovenia	35.5	26.1	48.2	3.5	2.6	4.8	13.1	9.6	17.6	1.5	1.1	2.0
Solomon Islands	12.0	7.8	16.0	7.9	5.2	10.6	30.1	20.4	39.8	9.2	6.2	12.1
Somalia	91.5	63.1	126.1	2.9	2.0	4.1	197.9	134.8	276.8	2.1	1.4	2.9
South Africa	392.5	357.7	425.5	2.1	1.9	2.2	287.6	252.9	325.6	0.9	0.8	1.1
South Sudan	38.3	24.9	57.7	1.4	0.9	2.1	48.7	30.4	77.0	1.2	0.7	1.8
Spain	482.8	455.9	508.5	2.5	2.3	2.6	306.1	261.9	351.6	1.5	1.3	1.7
Sri Lanka	129.5	112.0	147.5	1.4	1.2	1.6	91.8	65.0	124.9	0.8	0.6	1.1
Sudan	281.4	181.7	370.3	3.1	2.0	4.1	466.6	270.6	710.7	2.2	1.3	3.4
Suriname	3.1	2.4	3.6	1.6	1.2	1.9	4.5	3.5	5.6	1.5	1.2	2.0
Sweden	64.2	59.6	69.4	1.5	1.4	1.7	22.5	20.1	25.0	0.5	0.4	0.6
Switzerland	68.6	63.2	73.9	1.9	1.7	2.0	26.9	22.6	31.4	0.7	0.6	0.8
Syrian Arab Republic	66.5	50.4	85.1	1.2	0.9	1.5	71.2	49.4	101.1	1.0	0.7	1.4
Tajikistan	139.8	125.7	158.6	5.8	5.2	6.6	221.4	174.2	282.1	4.5	3.5	5.7
Thailand	779.7	660.0	915.4	2.5	2.1	2.9	668.0	470.0	903.8	1.9	1.3	2.5
Timor-Leste	7.3	4.7	10.2	1.9	1.2	2.7	6.5	2.0	9.6	1.0	0.3	1.5
Togo	30.9	24.3	38.8	1.9	1.5	2.4	66.1	45.1	91.6	1.7	1.1	2.3
Tokelau	0.0	0.0	0.0	3.1	2.1	4.4	0.0	0.0	0.0	2.6	1.6	3.7
Tonga	1.4	1.1	1.7	3.1	2.5	3.9	1.6	1.1	2.2	3.2	2.3	4.5
Trinidad and Tobago	8.3	7.4	9.2	1.3	1.2	1.5	5.4	3.7	7.5	0.8	0.5	1.1
Tunisia	37.6	30.5	46.4	0.9	0.7	1.1	56.3	38.8	78.2	0.9	0.6	1.3
Turkey	1389.6	1132.6	1645.9	4.6	3.8	5.5	1046.4	812.0	1329.9	2.3	1.8	2.9
Turkmenistan	62.7	57.6	68.2	3.6	3.3	3.9	56.0	43.3	72.9	2.1	1.6	2.8
Tuvalu	0.2	0.2	0.3	5.3	3.9	6.9	0.2	0.2	0.3	4.0	2.7	5.5

Country	Early-onset gastric cancer (15–49 years)											
	1990						2019					
	Death number			Death rate (per 100 000)			Death number			Death rate (per 100 000)		
	Value	LUI	UUI	Value	LUI	UUI	Value	LUI	UUI	Value	LUI	UUI
Uganda	102.1	73.2	134.0	1.4	1.0	1.8	256.6	186.8	354.8	1.4	1.0	1.9
Ukraine	2269.1	2129.6	2407.8	9.1	8.5	9.7	1099.7	872.2	1359.3	5.3	4.2	6.5
United Arab Emirates	11.7	8.6	15.5	1.0	0.7	1.3	76.5	52.6	108.2	1.1	0.8	1.6
United Kingdom	356.1	349.0	364.7	1.3	1.2	1.3	231.3	224.0	240.1	0.8	0.7	0.8
United Republic of Tanzania	174.4	133.5	225.3	1.5	1.2	2.0	323.7	235.7	441.9	1.2	0.9	1.7
United States of America	1215.5	1181.8	1245.2	0.9	0.9	0.9	1107.4	1060.5	1159.7	0.7	0.7	0.8
United States Virgin Islands	1.3	1.0	1.5	2.3	1.8	2.8	0.8	0.6	1.1	1.9	1.3	2.6
Uruguay	44.4	40.5	48.8	3.0	2.7	3.3	30.5	26.5	35.0	1.9	1.6	2.1
Uzbekistan	423.4	393.0	451.9	4.3	4.0	4.6	536.7	435.2	656.7	3.0	2.4	3.6
Vanuatu	2.8	1.8	3.9	4.0	2.6	5.6	6.6	4.2	9.0	4.5	2.9	6.2
Venezuela	236.3	219.1	255.4	2.5	2.3	2.7	357.1	258.0	482.3	2.5	1.8	3.4
Viet Nam	819.3	596.0	1089.0	2.5	1.8	3.3	1196.9	872.3	1598.5	2.3	1.6	3.0
Yemen	186.4	113.2	264.3	3.4	2.1	4.8	471.4	302.5	675.3	3.0	1.9	4.3
Zambia	68.1	53.2	85.0	1.9	1.5	2.4	138.5	93.1	194.0	1.5	1.0	2.1
Zimbabwe	70.9	57.6	84.9	1.5	1.3	1.8	186.9	126.6	254.3	2.5	1.7	3.4

LUI, lower uncertainty intervals; UUI, upper uncertainty intervals

Table S6. Deaths due to intermediate-onset gastric cancer in 203 countries, 2019 versus 1990

Country	Intermediate-onset gastric cancer (50–54 years)											
	1990						2019					
	Death number			Death rate (per 100 000)			Death number			Death rate (per 100 000)		
	Value	LUI	UUI	Value	LUI	UUI	Value	LUI	UUI	Value	LUI	UUI
Afghanistan	214.7	123.8	309.1	50.5	29.1	72.7	512.4	293.6	736.7	43.4	24.8	62.3
Albania	24.1	20.7	27.6	18.6	16.0	21.3	21.1	14.2	29.6	11.4	7.7	16.0
Algeria	61.8	43.7	85.1	8.9	6.3	12.2	94.4	65.1	129.8	4.6	3.2	6.3
American Samoa	0.4	0.3	0.5	23.9	17.6	32.9	0.6	0.4	0.8	18.0	12.8	24.3
Andorra	0.3	0.2	0.4	11.1	7.5	15.9	0.5	0.4	0.8	7.2	4.9	10.2
Angola	66.5	42.6	96.5	24.0	15.4	34.8	99.4	68.0	142.8	12.1	8.3	17.4
Antigua and Barbuda	0.4	0.3	0.5	20.6	16.3	25.8	0.6	0.5	0.8	9.9	7.4	13.2
Argentina	247.8	221.2	276.2	16.3	14.6	18.2	254.4	212.1	302.1	11.1	9.3	13.2
Armenia	67.4	60.3	74.6	36.9	33.0	40.8	26.1	20.2	33.4	15.4	11.9	19.7
Australia	57.7	52.3	63.3	7.2	6.5	7.9	57.2	45.4	70.8	3.8	3.0	4.6
Austria	66.5	59.8	73.6	14.9	13.4	16.4	36.8	30.7	43.2	5.2	4.3	6.1
Azerbaijan	185.0	159.8	216.9	51.8	44.7	60.7	159.7	110.7	218.4	24.1	16.7	32.9
Bahamas	1.9	1.5	2.3	20.5	16.7	24.7	2.8	2.1	3.7	11.4	8.5	14.9
Bahrain	1.8	1.3	2.3	12.9	9.5	17.1	3.3	2.3	4.7	3.3	2.3	4.6
Bangladesh	508.1	361.1	688.2	19.1	13.6	25.9	703.0	478.6	1003.5	9.8	6.6	13.9
Barbados	1.6	1.3	1.8	16.3	13.9	19.1	2.0	1.5	2.7	9.5	7.1	12.5
Belarus	401.1	365.5	443.5	58.6	53.4	64.8	129.7	89.9	179.8	20.4	14.2	28.3
Belgium	50.9	45.0	57.3	8.6	7.6	9.7	36.3	30.3	44.1	4.5	3.8	5.5
Belize	0.7	0.6	0.8	14.6	12.3	17.3	2.4	1.9	2.9	13.5	10.8	16.7
Benin	21.8	15.5	29.3	20.6	14.7	27.7	42.7	28.0	64.9	13.6	8.9	20.7
Bermuda	0.4	0.3	0.5	14.1	11.0	17.6	0.3	0.2	0.3	5.0	3.7	6.8
Bhutan	2.4	1.5	3.6	15.2	9.2	22.3	2.9	1.8	4.3	9.2	5.7	13.7
Bolivia	100.6	72.5	131.7	55.8	40.2	73.0	153.9	97.9	225.0	31.7	20.2	46.4
Bosnia and Herzegovina	49.9	43.8	56.8	18.1	15.9	20.6	28.6	20.4	38.5	11.8	8.4	15.8
Botswana	5.5	3.5	8.2	16.7	10.4	24.8	10.4	6.6	15.7	12.0	7.6	18.1
Brazil	1263.7	1198.1	1328.4	24.5	23.2	25.7	1528.0	1430.2	1636.5	12.1	11.3	12.9
Brunei Darussalam	2.0	1.5	2.6	30.1	23.1	38.7	2.9	2.2	3.7	12.1	9.4	15.6
Bulgaria	133.1	119.0	147.7	25.4	22.7	28.2	72.2	50.4	99.3	15.1	10.5	20.7
Burkina Faso	52.2	35.9	74.7	20.5	14.1	29.3	94.5	66.3	134.4	16.0	11.2	22.7
Burundi	29.0	20.0	40.8	22.2	15.4	31.3	39.3	25.1	58.9	12.9	8.2	19.3
Cabo Verde	3.5	2.7	4.6	36.2	27.5	47.7	5.4	3.7	7.6	21.6	14.9	30.0
Cambodia	67.5	47.8	92.5	24.5	17.3	33.5	86.2	58.2	121.3	11.4	7.7	16.1
Cameroon	57.5	41.3	77.6	20.0	14.4	27.0	110.8	70.1	166.4	14.2	9.0	21.4
Canada	96.6	89.0	105.1	7.4	6.9	8.1	99.5	76.1	122.8	4.0	3.1	5.0
Central African Republic	24.9	17.6	34.1	32.1	22.6	43.9	38.9	23.8	57.8	23.5	14.4	34.9
Chad	28.8	21.1	39.4	19.8	14.5	27.1	63.0	41.6	91.2	17.4	11.5	25.2
Chile	184.4	166.3	204.1	35.3	31.8	39.1	173.1	141.6	208.4	14.6	12.0	17.6
China	24930.4	20978.5	29087.7	52.2	43.9	60.9	27627.0	22222.5	33723.8	22.1	17.8	27.0
Colombia	337.9	308.0	370.6	32.5	29.6	35.7	403.1	284.4	557.1	14.6	10.3	20.2
Comoros	1.7	0.6	2.8	13.6	4.7	22.0	2.6	1.6	3.8	8.9	5.6	13.1
Congo	16.9	11.8	23.2	26.1	18.3	35.8	22.2	14.6	32.6	11.6	7.6	17.1
Cook Islands	0.1	0.1	0.1	13.1	9.4	17.8	0.1	0.1	0.1	7.6	5.2	10.6
Costa Rica	37.7	33.1	42.6	41.1	36.1	46.4	53.6	36.2	74.9	19.7	13.3	27.6
Croatia	86.1	75.6	97.2	26.9	23.6	30.4	29.8	20.7	39.9	9.8	6.8	13.1
Cuba	46.6	41.2	52.3	9.4	8.3	10.6	67.2	49.6	89.9	6.6	4.9	8.8
Cyprus	3.0	2.3	3.9	8.2	6.2	10.7	4.2	3.2	5.5	5.2	3.9	6.8
Czechia	94.0	85.5	102.8	18.2	16.5	19.9	41.5	30.8	53.9	6.0	4.5	7.8
Cote d'Ivoire	67.1	47.7	92.6	23.1	16.4	32.0	109.3	71.6	158.1	14.6	9.6	21.2
Democratic People's Republic of Korea	661.0	423.2	990.5	54.1	34.6	81.1	722.6	468.3	1064.8	35.7	23.1	52.6
Democratic Republic of the Congo	171.7	117.1	241.6	16.9	11.5	23.8	295.6	192.1	430.9	11.9	7.7	17.3
Denmark	32.3	28.4	36.2	11.4	10.0	12.8	17.3	13.2	22.4	4.1	3.2	5.3
Djibouti	1.5	0.9	2.2	13.8	8.6	20.2	4.6	2.8	7.3	10.3	6.2	16.3
Dominica	0.8	0.6	1.0	31.8	24.5	40.1	0.9	0.7	1.3	20.3	14.3	27.5
Dominican Republic	21.4	17.1	26.7	10.3	8.2	12.8	60.9	39.2	91.8	11.7	7.5	17.6
Ecuador	99.8	88.9	110.5	33.4	29.8	37.0	167.1	121.4	231.1	20.9	15.2	28.8
Egypt	124.3	95.5	158.6	6.8	5.2	8.7	237.4	150.9	350.5	5.7	3.6	8.4
El Salvador	35.7	31.2	40.5	22.8	19.9	25.9	53.8	36.5	77.2	18.7	12.7	26.8
Equatorial Guinea	3.3	2.1	4.9	25.4	16.5	37.9	2.3	1.3	3.8	7.1	4.0	11.9
Eritrea	18.0	12.0	25.0	23.3	15.5	32.3	32.9	21.0	48.8	16.7	10.7	24.8
Estonia	42.0	37.4	46.9	41.3	36.8	46.2	11.6	7.8	16.4	14.2	9.5	20.0
Eswatini	3.1	2.1	4.5	16.1	11.1	23.1	4.7	2.9	7.2	13.9	8.5	21.3
Ethiopia	349.6	264.3	438.6	27.2	20.6	34.1	209.7	157.4	279.4	8.2	6.2	11.0

Country	Intermediate-onset gastric cancer (50–54 years)											
	1990						2019					
	Death number			Death rate (per 100 000)			Death number			Death rate (per 100 000)		
	Value	LUI	UUI	Value	LUI	UUI	Value	LUI	UUI	Value	LUI	UUI
Fiji	2.7	1.9	3.8	10.8	7.6	15.1	4.1	2.8	5.8	8.5	5.7	12.0
Finland	35.2	31.2	39.7	12.8	11.3	14.4	16.8	12.7	21.7	4.7	3.6	6.1
France	262.3	234.8	291.8	8.9	7.9	9.9	220.9	169.7	279.2	5.0	3.9	6.4
Gabon	6.2	4.4	8.7	20.0	14.0	28.0	7.3	4.8	10.5	10.4	6.8	15.0
Gambia	1.4	0.9	2.1	6.4	4.1	9.8	2.9	1.8	4.2	5.2	3.3	7.5
Georgia	122.0	104.8	143.1	33.7	29.0	39.6	48.3	35.9	63.4	21.1	15.7	27.7
Germany	790.8	713.3	875.8	12.9	11.6	14.3	477.3	383.5	579.9	7.0	5.6	8.5
Ghana	52.4	36.6	72.4	13.2	9.2	18.2	79.8	53.7	115.3	7.8	5.2	11.2
Greece	92.4	84.0	101.1	14.4	13.1	15.8	63.5	54.7	72.5	8.3	7.2	9.5
Greenland	0.7	0.5	0.9	28.4	21.2	37.8	0.7	0.5	1.0	14.3	10.0	20.1
Grenada	0.5	0.4	0.6	20.8	16.5	25.7	0.7	0.6	0.9	10.0	7.8	12.7
Guam	0.4	0.3	0.5	7.4	5.5	9.6	0.8	0.6	1.2	7.9	5.6	10.8
Guatemala	75.2	63.8	87.8	31.9	27.1	37.3	174.1	129.1	229.2	30.5	22.6	40.1
Guinea	37.3	27.2	50.5	21.7	15.8	29.4	63.9	42.7	88.7	20.1	13.4	27.9
Guinea-Bissau	9.3	6.2	13.3	38.9	26.1	55.4	11.3	7.6	16.2	23.8	16.1	34.1
Guyana	3.8	3.0	4.8	17.7	13.8	22.5	4.8	3.3	6.8	12.4	8.5	17.4
Haiti	67.4	43.7	92.4	34.9	22.7	47.9	88.8	52.9	134.4	20.5	12.2	31.1
Honduras	21.3	15.7	28.5	17.9	13.2	23.9	59.3	37.7	89.4	17.4	11.1	26.2
Hungary	127.4	114.3	140.9	21.3	19.1	23.5	68.0	48.9	91.4	10.5	7.5	14.1
Iceland	1.2	1.0	1.5	11.7	9.2	14.5	0.9	0.7	1.3	4.4	3.3	5.9
India	4730.6	4092.2	5367.2	16.1	13.9	18.2	7272.0	5896.3	8805.3	11.4	9.3	13.8
Indonesia	819.6	657.6	953.5	12.5	10.0	14.5	1029.0	808.1	1279.7	7.0	5.5	8.6
Iran	441.7	380.0	489.7	27.9	24.0	30.9	650.6	600.4	706.7	14.5	13.4	15.7
Iraq	50.0	34.4	69.9	11.1	7.7	15.6	125.6	84.3	175.8	8.1	5.4	11.3
Ireland	17.0	14.7	19.4	10.8	9.4	12.3	13.4	10.9	16.2	4.3	3.5	5.2
Israel	18.3	16.0	20.7	9.6	8.4	10.9	24.5	20.4	29.3	5.6	4.6	6.7
Italy	610.2	590.4	629.8	16.8	16.3	17.4	369.0	347.7	392.8	7.5	7.0	7.9
Jamaica	10.0	7.9	12.3	14.0	11.1	17.2	16.5	11.1	23.4	10.7	7.2	15.2
Japan	2871.7	2797.2	2937.4	35.0	34.1	35.8	944.1	894.8	988.0	10.9	10.3	11.4
Jordan	10.4	7.7	14.1	9.9	7.3	13.4	24.9	17.7	34.2	4.9	3.5	6.7
Kazakhstan	513.7	464.7	564.3	57.8	52.3	63.5	183.2	142.6	232.4	18.4	14.4	23.4
Kenya	56.9	43.1	75.8	11.7	8.8	15.5	176.2	137.2	219.8	11.9	9.3	14.8
Kiribati	0.7	0.5	1.0	31.3	21.5	42.8	1.1	0.7	1.6	23.7	15.3	34.1
Kuwait	2.6	2.0	3.3	5.3	4.2	6.7	5.9	4.1	8.3	2.3	1.6	3.2
Kyrgyzstan	111.0	97.8	123.7	62.8	55.4	70.0	77.4	60.4	95.4	25.8	20.2	31.9
Lao People's Democratic Republic	35.4	23.3	50.8	28.4	18.7	40.7	28.7	18.6	41.8	9.9	6.4	14.4
Latvia	72.0	65.1	78.6	40.3	36.5	44.0	20.4	15.3	26.9	15.8	11.8	20.8
Lebanon	15.5	11.3	20.8	11.5	8.4	15.5	17.9	12.2	25.3	7.1	4.8	10.0
Lesotho	8.2	5.5	11.9	15.3	10.3	22.2	14.7	9.4	21.5	20.1	12.8	29.6
Liberia	10.6	7.2	14.9	18.8	12.8	26.4	16.0	10.1	24.5	11.8	7.4	18.1
Libya	10.0	6.8	14.1	8.9	6.0	12.5	23.2	15.3	34.0	6.3	4.2	9.2
Lithuania	85.0	75.9	94.1	38.5	34.4	42.7	33.1	24.1	45.2	16.3	11.9	22.3
Luxembourg	2.8	2.3	3.4	12.4	10.3	14.9	1.8	1.4	2.2	3.8	3.0	4.7
Madagascar	40.5	29.9	53.4	13.8	10.1	18.1	78.2	50.8	113.4	9.7	6.3	14.1
Malawi	12.0	8.8	16.7	5.4	3.9	7.4	15.7	10.4	22.0	3.7	2.5	5.2
Malaysia	68.4	53.7	85.4	11.2	8.8	14.0	92.1	64.0	128.8	6.1	4.2	8.5
Maldives	0.9	0.6	1.2	13.0	9.2	17.6	0.6	0.4	0.7	2.6	1.9	3.5
Mali	92.6	68.5	122.2	38.2	28.2	50.4	139.0	94.3	198.5	25.8	17.5	36.8
Malta	1.9	1.5	2.2	9.8	8.1	11.7	1.0	0.8	1.3	4.0	3.1	5.0
Marshall Islands	0.2	0.2	0.3	28.3	20.3	38.2	0.5	0.4	0.8	22.7	15.4	32.4
Mauritania	12.0	8.6	16.1	23.0	16.5	30.8	11.2	6.9	16.7	9.3	5.7	13.7
Mauritius	6.0	5.0	7.0	15.3	13.0	18.0	6.4	4.7	8.5	6.9	5.1	9.2
Mexico	331.6	319.5	343.5	13.3	12.8	13.8	698.9	585.3	827.5	10.5	8.8	12.4
Micronesia	0.7	0.5	0.9	29.4	20.3	41.7	1.2	0.8	1.7	24.7	16.4	35.3
Monaco	0.2	0.2	0.3	11.1	7.9	14.9	0.2	0.1	0.3	5.9	3.9	8.3
Mongolia	56.3	41.2	74.0	99.8	73.0	131.2	105.6	71.8	148.8	58.0	39.4	81.7
Montenegro	3.9	3.1	4.8	10.6	8.5	13.1	3.4	2.6	4.4	8.3	6.3	10.7
Morocco	48.0	34.3	64.1	6.1	4.4	8.1	82.2	54.0	120.1	4.3	2.8	6.3
Mozambique	29.9	20.5	41.3	7.6	5.2	10.4	56.7	35.4	81.8	8.0	5.0	11.6
Myanmar	318.7	212.3	455.2	23.9	15.9	34.2	251.0	177.3	340.5	8.7	6.2	11.9
Namibia	2.0	1.3	2.8	5.4	3.5	7.6	3.3	2.2	4.7	3.9	2.6	5.7
Nauru	0.1	0.1	0.1	30.8	20.3	44.6	0.1	0.1	0.1	24.4	16.5	35.2
Nepal	99.2	67.3	138.1	16.4	11.2	22.9	135.3	90.7	191.3	10.9	7.3	15.4

Country	Intermediate-onset gastric cancer (50–54 years)											
	1990						2019					
	Death number			Death rate (per 100 000)			Death number			Death rate (per 100 000)		
	Value	LUI	UUI	Value	LUI	UUI	Value	LUI	UUI	Value	LUI	UUI
Netherlands	84.5	76.5	92.8	10.8	9.8	11.9	67.1	53.9	82.9	5.3	4.2	6.5
New Zealand	15.2	13.1	17.5	9.8	8.5	11.3	14.7	12.4	17.6	4.9	4.1	5.9
Nicaragua	16.4	13.3	19.9	18.4	14.9	22.3	36.6	26.6	50.7	13.7	10.0	19.0
Niger	39.2	27.2	53.3	21.5	14.9	29.3	73.3	48.5	111.4	14.6	9.7	22.2
Nigeria	134.6	97.7	181.9	5.2	3.7	7.0	216.0	155.4	293.9	3.5	2.5	4.7
Niue	0.0	0.0	0.0	16.7	11.4	23.3	0.0	0.0	0.0	11.9	7.8	17.0
North Macedonia	29.7	25.2	34.6	28.4	24.1	33.0	24.2	17.4	33.3	16.0	11.5	22.0
Northern Mariana Islands	0.3	0.2	0.4	21.0	14.9	28.9	0.6	0.4	0.8	12.1	8.4	16.6
Norway	19.7	18.6	20.7	9.9	9.4	10.4	13.5	12.3	14.8	3.7	3.4	4.0
Oman	7.5	4.9	11.0	14.9	9.7	21.9	7.7	5.4	10.8	5.0	3.5	7.1
Pakistan	289.4	223.0	364.7	8.7	6.7	11.0	632.5	468.6	845.1	8.6	6.4	11.5
Palau	0.1	0.1	0.1	19.6	13.0	29.6	0.2	0.2	0.4	16.1	10.6	24.5
Palestine	6.8	4.6	9.8	14.8	10.0	21.2	14.5	10.6	18.9	8.3	6.1	10.8
Panama	16.3	13.9	19.0	20.3	17.2	23.5	26.8	18.5	37.0	12.3	8.5	16.9
Papua New Guinea	22.9	14.2	34.5	19.2	11.9	28.9	64.0	38.6	96.7	18.0	10.8	27.2
Paraguay	15.2	12.0	19.2	12.7	10.0	16.0	32.6	21.6	46.5	10.8	7.1	15.3
Peru	198.3	159.6	246.1	29.6	23.8	36.7	299.4	198.7	444.9	17.5	11.6	26.0
Philippines	197.0	172.3	224.5	10.3	9.0	11.7	288.2	225.3	362.5	5.8	4.5	7.3
Poland	508.3	489.1	527.0	26.4	25.4	27.3	270.4	214.3	336.8	11.8	9.4	14.7
Portugal	174.3	157.2	192.2	29.9	27.0	33.0	121.4	101.7	141.8	15.6	13.1	18.2
Puerto Rico	17.2	14.8	19.8	10.4	9.0	12.0	11.5	7.9	16.3	4.9	3.3	6.9
Qatar	1.1	0.8	1.6	9.0	6.4	12.5	3.7	2.4	5.4	3.0	2.0	4.5
Republic of Korea	1810.3	1639.4	1988.2	88.7	80.4	97.5	623.0	505.6	766.5	14.0	11.3	17.2
Republic of Moldova	90.3	79.6	102.3	37.2	32.8	42.2	34.6	26.6	43.2	14.8	11.4	18.5
Romania	320.8	284.7	358.7	21.8	19.4	24.4	213.0	156.3	277.5	15.1	11.1	19.7
Russian Federation	5080.8	4753.9	5247.9	48.4	45.3	50.0	1530.0	1289.8	1811.9	17.4	14.7	20.6
Rwanda	38.9	28.2	51.4	22.8	16.5	30.1	34.4	22.8	49.8	9.2	6.1	13.4
Saint Kitts and Nevis	0.2	0.2	0.3	21.1	17.0	26.3	0.5	0.3	0.6	11.1	8.0	15.1
Saint Lucia	1.0	0.8	1.2	25.0	20.2	30.8	1.7	1.3	2.3	13.8	10.1	18.1
Saint Vincent and the Grenadines	0.5	0.4	0.6	17.6	14.1	21.7	1.1	0.8	1.4	15.0	11.5	19.3
Samoa	1.1	0.7	1.5	21.8	14.5	31.1	1.5	1.0	2.2	16.3	10.8	23.9
San Marino	0.3	0.2	0.4	22.7	16.6	30.3	0.4	0.2	0.6	14.7	8.2	23.9
Sao Tome and Principe	0.7	0.4	0.9	19.4	12.4	27.0	1.1	0.8	1.6	16.3	10.8	23.2
Saudi Arabia	30.1	19.5	44.9	7.6	4.9	11.3	66.8	44.8	94.2	4.2	2.8	5.9
Senegal	37.7	26.0	51.0	20.0	13.8	27.2	58.9	39.4	84.3	13.1	8.8	18.7
Serbia	110.9	85.0	141.9	18.0	13.8	23.0	62.8	44.6	87.9	11.6	8.2	16.2
Seychelles	0.4	0.3	0.5	16.2	12.1	21.1	0.6	0.4	0.8	8.5	6.3	11.4
Sierra Leone	19.0	12.8	26.9	18.5	12.5	26.3	31.3	20.6	45.9	14.1	9.3	20.7
Singapore	24.1	20.2	28.5	18.0	15.1	21.3	16.1	12.4	20.3	3.8	3.0	4.8
Slovakia	59.0	51.5	67.6	23.8	20.7	27.2	31.4	21.6	43.8	9.0	6.2	12.6
Slovenia	28.3	20.1	38.3	24.6	17.5	33.3	12.9	8.6	18.6	8.4	5.6	12.1
Solomon Islands	4.2	2.6	6.1	45.5	28.1	65.9	8.7	5.6	12.2	39.7	25.3	55.4
Somalia	44.6	28.0	66.2	23.6	14.8	35.0	63.9	39.8	93.3	17.8	11.1	26.0
South Africa	140.9	123.4	159.6	12.4	10.9	14.1	171.5	140.3	205.7	6.8	5.5	8.1
South Sudan	18.6	11.4	28.9	13.7	8.4	21.2	25.1	15.0	39.2	8.7	5.2	13.6
Spain	320.2	292.1	351.2	16.3	14.9	17.9	257.1	207.1	313.7	7.2	5.8	8.7
Sri Lanka	68.9	53.2	86.6	10.9	8.4	13.7	69.4	45.5	101.8	5.3	3.5	7.8
Sudan	127.4	79.4	174.4	24.1	15.0	33.0	217.0	130.4	341.8	17.3	10.4	27.2
Suriname	2.1	1.6	2.6	13.6	10.4	16.9	3.4	2.4	4.8	9.2	6.5	13.0
Sweden	36.7	33.0	40.7	7.9	7.1	8.7	20.9	17.9	24.1	3.1	2.7	3.6
Switzerland	42.2	37.8	47.6	10.7	9.6	12.1	25.8	20.4	32.6	3.8	3.0	4.9
Syrian Arab Republic	22.8	16.8	30.3	7.3	5.4	9.8	41.9	28.0	61.1	4.9	3.3	7.1
Tajikistan	96.7	84.1	112.6	57.5	50.0	67.0	104.9	75.9	144.6	26.6	19.2	36.7
Thailand	388.4	301.8	482.4	16.8	13.0	20.9	397.1	262.6	588.5	7.0	4.7	10.4
Timor-Leste	3.4	2.3	4.9	15.2	10.3	21.9	3.6	2.0	5.4	8.6	4.8	13.0
Togo	16.4	11.5	23.4	20.4	14.3	29.0	39.5	25.0	58.2	15.2	9.6	22.4
Tokelau	0.0	0.0	0.0	16.9	11.3	24.6	0.0	0.0	0.0	10.9	7.2	15.8
Tonga	0.6	0.4	0.8	19.0	13.5	25.6	0.7	0.5	1.0	15.5	10.2	23.0
Trinidad and Tobago	4.9	4.0	5.9	11.5	9.4	13.8	3.8	2.5	5.5	4.4	2.9	6.3
Tunisia	20.8	15.1	29.2	7.5	5.5	10.5	33.9	21.4	49.1	5.0	3.1	7.2
Turkey	634.1	469.1	831.9	30.6	22.6	40.1	670.3	487.6	907.8	14.0	10.2	19.0
Turkmenistan	43.6	38.4	49.8	37.8	33.3	43.1	29.8	21.1	41.1	11.6	8.3	16.1
Tuvalu	0.1	0.1	0.1	25.7	18.2	35.0	0.1	0.1	0.1	17.4	11.3	26.3

Country	Intermediate-onset gastric cancer (50–54 years)											
	1990						2019					
	Death number			Death rate (per 100 000)			Death number			Death rate (per 100 000)		
	Value	LUI	UUI	Value	LUI	UUI	Value	LUI	UUI	Value	LUI	UUI
Uganda	53.6	37.0	73.8	13.6	9.4	18.7	111.0	73.1	159.3	11.9	7.8	17.0
Ukraine	2148.4	1956.4	2364.6	54.8	49.9	60.3	721.9	550.0	944.6	25.1	19.1	32.8
United Arab Emirates	5.3	3.6	7.5	11.3	7.8	16.0	34.8	21.9	51.9	6.2	3.9	9.3
United Kingdom	284.7	278.0	291.2	9.1	8.9	9.3	194.9	187.3	203.9	4.2	4.0	4.4
United Republic of Tanzania	92.4	65.0	127.0	14.2	10.0	19.5	155.5	101.7	224.9	9.7	6.4	14.1
United States of America	705.5	682.6	726.7	6.2	6.0	6.3	829.0	783.8	876.3	4.0	3.8	4.2
United States Virgin Islands	0.7	0.5	0.9	13.1	9.7	17.6	1.0	0.7	1.3	12.7	8.8	17.9
Uruguay	33.4	29.2	37.9	20.8	18.2	23.7	23.5	18.9	28.4	11.9	9.6	14.4
Uzbekistan	272.1	244.2	304.4	40.8	36.7	45.7	288.3	221.9	364.3	18.1	13.9	22.8
Vanuatu	0.9	0.5	1.3	21.8	13.7	34.0	2.1	1.3	3.2	21.1	13.3	31.4
Venezuela	122.6	108.6	136.6	22.0	19.5	24.5	224.2	152.0	318.1	14.1	9.5	20.0
Viet Nam	523.0	361.7	731.7	26.7	18.5	37.4	871.0	593.4	1236.2	14.8	10.1	21.0
Yemen	94.5	59.2	144.0	31.9	20.0	48.6	204.3	125.4	313.6	25.4	15.6	39.0
Zambia	32.1	23.0	43.1	17.0	12.2	22.9	56.3	37.5	80.7	12.4	8.2	17.8
Zimbabwe	41.4	31.9	53.7	16.3	12.6	21.2	76.2	51.6	107.4	18.6	12.6	26.3

LUI, lower uncertainty intervals; UUI, upper uncertainty intervals

Table S7. Deaths due to later-onset gastric cancer in 203 countries, 2019 versus 1990

Country	Later-onset gastric cancer (≥ 55 years)											
	1990						2019					
	Death number			Death rate (per 100 000)			Death number			Death rate (per 100 000)		
	Value	LUI	UUI	Value	LUI	UUI	Value	LUI	UUI	Value	LUI	UUI
Afghanistan	1637.6	1216.6	2066.1	132.1	98.1	166.7	2062.1	1526.6	2560.3	125.9	93.2	156.3
Albania	254.4	235.6	272.0	73.2	67.7	78.2	399.0	300.5	526.8	51.8	39.0	68.5
Algeria	744.1	597.9	910.1	35.7	28.7	43.7	1212.4	985.2	1481.2	21.6	17.5	26.4
American Samoa	2.8	2.5	3.2	76.8	67.7	87.7	5.3	4.6	6.1	63.7	55.0	73.5
Andorra	5.3	4.1	7.2	55.1	43.1	74.6	10.9	8.3	13.8	45.6	34.8	57.7
Angola	366.9	283.0	462.9	59.5	45.9	75.1	650.6	526.0	812.2	36.1	29.2	45.1
Antigua and Barbuda	7.9	7.2	8.7	91.8	83.0	100.6	9.2	7.8	10.6	51.9	44.3	59.8
Argentina	3966.7	3773.4	4145.3	71.2	67.8	74.4	4462.4	4115.3	4824.2	49.2	45.4	53.2
Armenia	464.4	441.5	489.5	96.2	91.5	101.4	467.6	390.0	547.8	61.2	51.1	71.7
Australia	1263.8	1193.5	1317.2	38.5	36.4	40.1	1539.3	1370.5	1692.8	22.3	19.8	24.5
Austria	1906.4	1809.3	1983.1	97.9	92.9	101.8	956.4	860.2	1044.8	33.2	29.9	36.3
Azerbaijan	1130.6	1039.6	1223.0	129.8	119.3	140.4	1513.8	1253.0	1859.2	87.8	72.6	107.8
Bahamas	16.0	14.4	17.7	65.2	58.9	72.1	26.6	21.7	32.3	39.4	32.1	47.8
Bahrain	15.9	13.6	18.3	56.2	48.0	64.7	31.6	25.2	39.6	17.2	13.7	21.6
Bangladesh	3801.0	3167.7	4419.8	50.6	42.2	58.9	6459.8	4951.4	8448.6	29.6	22.7	38.7
Barbados	44.8	40.5	48.9	95.5	86.3	104.3	46.2	38.2	54.3	53.3	44.0	62.6
Belarus	3393.0	3244.2	3530.0	146.2	139.8	152.1	1930.0	1523.0	2438.8	67.4	53.2	85.1
Belgium	1753.9	1632.6	1853.9	66.9	62.2	70.7	1203.8	1073.4	1322.0	32.8	29.2	36.0
Belize	10.0	8.9	11.1	64.9	57.9	72.0	20.5	17.7	23.7	44.9	38.7	51.8
Benin	267.5	232.1	308.2	83.5	72.5	96.3	467.1	376.7	571.6	60.9	49.1	74.5
Bermuda	6.7	6.1	7.4	63.4	57.6	69.8	6.3	5.2	7.5	28.1	23.4	33.5
Bhutan	15.6	11.3	20.4	39.4	28.3	51.4	35.9	26.8	46.0	38.6	28.9	49.6
Bolivia	1179.7	975.9	1378.9	224.8	186.0	262.8	2543.9	2033.9	3141.4	170.5	136.3	210.5
Bosnia and Herzegovina	434.6	409.9	460.0	59.6	56.2	63.1	543.0	435.8	685.0	51.1	41.0	64.5
Botswana	48.1	37.0	60.5	50.8	39.0	63.9	82.8	63.6	103.4	36.9	28.4	46.2
Brazil	12584.9	11923.7	13033.2	85.3	80.8	88.3	19096.2	17600.3	20119.7	46.9	43.2	49.4
Brunei Darussalam	20.5	18.0	23.1	136.9	120.3	154.7	25.9	22.4	29.3	49.7	43.1	56.3
Bulgaria	2060.7	1939.8	2172.8	91.0	85.7	96.0	1386.0	1123.0	1713.1	56.9	46.1	70.3
Burkina Faso	586.4	479.6	694.1	80.0	65.5	94.8	972.6	801.7	1155.1	66.3	54.7	78.8
Burundi	223.3	183.5	270.8	59.1	48.6	71.7	273.7	214.1	349.4	36.7	28.7	46.8
Cabo Verde	68.1	60.9	76.1	171.8	153.5	192.0	93.3	82.1	105.0	130.3	114.6	146.6
Cambodia	484.8	381.4	594.3	65.9	51.8	80.7	809.1	645.7	960.8	39.9	31.8	47.4
Cameroon	537.7	448.3	633.9	72.8	60.7	85.9	1153.5	907.2	1464.9	60.5	47.6	76.8
Canada	2199.6	2069.9	2298.4	40.3	37.9	42.1	2916.7	2601.4	3185.0	24.8	22.1	27.0
Central African Republic	138.2	111.6	166.8	72.2	58.3	87.1	171.8	128.9	221.9	50.7	38.0	65.4
Chad	365.1	306.8	429.4	78.9	66.3	92.8	646.7	512.8	802.7	72.7	57.6	90.2
Chile	2779.3	2642.5	2900.1	166.0	157.8	173.2	3854.6	3507.9	4163.2	92.0	83.7	99.3
China	241087	211180	272213	166.8	146.1	188.4	362798	305288	422538	103.1	86.8	120.1
Colombia	3979.7	3753.4	4157.9	138.8	130.9	145.0	5652.7	4406.0	7140.6	62.2	48.5	78.5
Comoros	16.5	10.8	21.3	45.7	29.9	58.9	25.8	21.3	32.2	32.8	27.0	40.8
Congo	114.0	93.1	135.7	64.3	52.5	76.5	147.8	120.6	181.0	36.0	29.3	44.0
Cook Islands	1.0	0.9	1.1	46.4	40.5	52.2	1.3	1.1	1.6	29.8	25.7	35.0
Costa Rica	525.8	486.9	555.6	184.8	171.1	195.3	832.8	653.1	1043.7	93.1	73.0	116.7
Croatia	1206.3	1130.8	1285.4	104.6	98.1	111.5	786.2	632.0	967.4	53.5	43.0	65.8
Cuba	664.7	623.2	698.7	39.1	36.7	41.1	971.3	793.2	1171.5	30.0	24.5	36.2
Cyprus	59.3	51.0	66.3	42.4	36.5	47.4	114.7	100.2	131.5	34.7	30.3	39.8
Czechia	2161.6	2064.9	2247.9	91.3	87.2	94.9	1189.0	971.8	1430.9	34.5	28.2	41.5
Cote d'Ivoire	485.3	396.7	580.9	75.7	61.9	90.6	1002.7	805.8	1230.6	59.6	47.9	73.2
Democratic People's Republic of Korea	3430.2	2716.8	4252.4	129.4	102.5	160.4	5668.5	4602.6	6836.9	105.1	85.4	126.8
Democratic Republic of the Congo	1288.2	1042.7	1574.7	48.0	38.9	58.7	1955.6	1513.8	2486.7	34.0	26.3	43.2
Denmark	690.3	655.2	718.7	52.9	50.2	55.1	473.6	426.5	519.3	25.5	22.9	27.9
Djibouti	8.7	6.6	11.5	39.8	30.2	52.7	31.2	23.9	41.2	32.8	25.2	43.4
Dominica	18.8	16.8	20.6	160.0	143.1	175.6	16.4	13.6	19.6	105.0	87.2	125.5
Dominican Republic	201.0	176.1	227.6	32.8	28.7	37.1	621.8	481.4	804.3	40.2	31.1	52.0
Ecuador	1317.6	1222.7	1395.6	152.0	141.1	161.0	2617.0	2091.4	3284.4	102.2	81.7	128.2
Egypt	1205.7	1089.7	1311.8	25.3	22.8	27.5	2283.7	1726.1	3005.3	21.1	15.9	27.8
El Salvador	381.5	349.1	409.8	78.2	71.6	84.0	831.6	638.8	1042.2	84.8	65.2	106.3
Equatorial Guinea	20.0	15.1	25.1	61.5	46.5	77.1	21.3	16.0	29.0	28.4	21.3	38.8
Eritrea	74.4	55.3	96.5	48.8	36.3	63.3	172.7	131.2	218.5	41.2	31.3	52.2
Estonia	454.0	432.0	474.0	126.6	120.5	132.2	272.0	216.3	339.2	62.8	49.9	78.3
Eswatini	22.3	17.8	27.0	48.6	38.7	58.9	35.1	27.3	44.9	37.7	29.3	48.2
Ethiopia	1937.4	1557.7	2405.6	60.4	48.6	75.0	1899.2	1561.3	2355.0	29.3	24.1	36.4

Country	Later-onset gastric cancer (≥ 55 years)											
	1990						2019					
	Death number			Death rate (per 100 000)			Death number			Death rate (per 100 000)		
	Value	LUI	UUI	Value	LUI	UUI	Value	LUI	UUI	Value	LUI	UUI
Fiji	18.7	15.3	22.9	33.2	27.1	40.6	35.7	28.6	44.5	27.2	21.8	34.0
Finland	853.2	807.4	890.5	71.9	68.0	75.0	571.1	510.9	627.8	29.0	25.9	31.9
France	7981.6	7453.5	8359.3	57.3	53.5	60.0	6751.4	5825.7	7423.5	31.3	27.0	34.4
Gabon	56.3	45.3	70.2	58.3	46.9	72.7	61.6	48.4	75.3	35.4	27.8	43.3
Gambia	16.4	13.0	20.5	29.4	23.4	36.9	41.5	33.2	50.3	27.6	22.1	33.5
Georgia	925.9	844.2	1011.1	84.3	76.9	92.1	712.9	599.2	841.9	67.4	56.7	79.7
Germany	15211.9	14269.3	15855.6	72.5	68.0	75.6	12933.9	11595.3	14086.8	42.2	37.9	46.0
Ghana	510.7	416.3	616.2	50.0	40.7	60.3	942.5	748.5	1172.1	36.3	28.9	45.2
Greece	1981.6	1871.6	2076.9	74.1	70.0	77.6	1910.0	1713.4	2087.2	52.5	47.1	57.3
Greenland	4.4	3.9	4.9	78.8	70.1	88.1	5.8	4.7	7.0	45.1	36.6	54.1
Grenada	10.8	9.7	11.9	89.2	79.9	98.3	9.3	8.4	10.4	47.8	42.8	53.5
Guam	4.1	3.6	4.7	32.2	28.1	36.6	8.3	6.9	9.8	25.0	20.8	29.5
Guatemala	773.3	692.7	852.6	126.1	112.9	139.0	2327.5	1863.8	2874.5	128.7	103.1	159.0
Guinea	417.6	354.7	487.3	75.5	64.1	88.1	651.2	522.4	799.3	73.2	58.7	89.8
Guinea-Bissau	75.2	59.0	91.0	115.8	90.8	140.0	91.0	72.2	112.7	80.6	63.9	99.9
Guyana	42.0	36.7	47.5	68.5	60.0	77.6	39.6	31.3	49.3	36.5	28.8	45.5
Haiti	547.5	381.8	655.9	101.0	70.4	121.0	774.1	505.3	1040.4	67.9	44.3	91.2
Honduras	193.5	157.9	226.2	57.7	47.1	67.4	743.3	615.3	932.9	74.4	61.6	93.4
Hungary	2584.3	2470.1	2688.8	100.4	95.9	104.4	1534.8	1279.0	1821.5	48.0	40.0	56.9
Iceland	34.4	31.6	37.1	72.0	66.3	77.6	24.5	21.1	27.6	26.3	22.6	29.6
India	31281.3	27697.6	34823.9	41.7	36.9	46.4	59070.2	50809.2	67879.9	30.9	26.6	35.5
Indonesia	5932.4	4898.4	6801.1	36.7	30.3	42.1	10278.3	8616.5	11627.7	28.0	23.4	31.6
Iran	4662.6	4095.3	5210.3	101.2	88.9	113.1	9210.9	8456.9	9917.7	75.9	69.7	81.7
Iraq	377.1	305.3	462.1	30.3	24.5	37.1	867.4	688.8	1033.5	23.6	18.7	28.1
Ireland	484.8	456.3	510.1	71.2	67.0	74.9	410.6	368.3	450.8	33.0	29.6	36.2
Israel	463.7	431.1	492.1	57.2	53.2	60.7	647.3	576.6	704.6	34.0	30.3	37.0
Italy	15209.5	14589.2	15586.3	99.9	95.9	102.4	12102.8	10709.6	12908.8	54.9	48.6	58.5
Jamaica	238.0	220.4	255.7	80.8	74.8	86.8	249.7	200.3	305.6	49.2	39.5	60.3
Japan	45798.5	43418.7	47024.9	154.6	146.6	158.7	55045.0	45904.9	59929.0	106.4	88.7	115.8
Jordan	72.2	60.4	85.7	33.5	28.1	39.8	218.5	181.3	262.9	20.5	17.0	24.6
Kazakhstan	3212.2	3046.4	3378.2	153.7	145.7	161.6	1958.6	1685.6	2247.0	64.4	55.4	73.9
Kenya	558.2	445.2	660.8	41.9	33.4	49.6	1482.1	1225.6	1767.4	41.9	34.6	50.0
Kiribati	4.3	3.5	5.2	73.0	59.3	87.9	6.5	5.1	8.2	56.2	43.7	70.5
Kuwait	23.7	21.0	26.9	25.7	22.9	29.3	56.6	47.1	68.7	14.3	11.9	17.3
Kyrgyzstan	701.9	660.3	742.4	133.9	126.0	141.7	568.2	493.7	645.5	72.0	62.5	81.8
Lao People's Democratic Republic	241.5	184.6	302.2	70.4	53.8	88.1	248.2	189.8	305.2	34.3	26.3	42.2
Latvia	724.6	694.4	753.7	115.8	111.0	120.5	410.2	345.5	493.6	61.9	52.1	74.4
Lebanon	174.2	145.3	210.7	44.0	36.7	53.2	302.2	246.3	401.7	34.9	28.4	46.4
Lesotho	80.0	63.9	98.2	48.1	38.4	59.1	108.5	82.6	138.1	51.4	39.2	65.5
Liberia	142.8	119.7	168.5	77.2	64.7	91.1	168.0	129.4	213.4	53.7	41.4	68.2
Libya	100.8	75.1	126.7	32.9	24.5	41.4	203.3	156.5	255.3	25.7	19.8	32.3
Lithuania	899.6	861.8	934.5	113.4	108.6	117.8	593.0	483.6	713.1	61.5	50.2	74.0
Luxembourg	60.6	56.3	64.9	64.9	60.3	69.5	45.8	38.9	52.2	27.6	23.5	31.5
Madagascar	327.5	278.9	379.8	38.4	32.7	44.5	483.0	366.8	623.8	27.6	20.9	35.6
Malawi	134.5	114.4	155.6	21.3	18.1	24.7	179.3	148.6	212.2	15.7	13.0	18.6
Malaysia	692.4	631.2	759.5	47.4	43.2	51.9	1435.3	1164.1	1778.3	31.0	25.1	38.4
Maldives	5.6	4.4	6.6	36.6	28.9	43.0	7.8	6.4	9.3	16.1	13.2	19.2
Mali	678.7	571.0	799.7	98.0	82.5	115.5	1103.1	902.0	1361.1	79.0	64.6	97.5
Malta	45.3	41.6	49.2	62.4	57.2	67.7	44.5	38.9	50.5	29.0	25.3	32.8
Marshall Islands	2.2	1.8	2.6	87.6	71.7	103.0	3.3	2.5	4.2	57.4	43.7	73.0
Mauritania	138.7	116.1	163.3	83.4	69.8	98.1	180.2	139.3	225.7	52.5	40.6	65.8
Mauritius	84.9	79.6	90.5	69.2	64.9	73.8	105.1	85.4	127.1	32.9	26.8	39.9
Mexico	4587.7	4337.3	4739.2	65.8	62.2	68.0	8062.3	6957.4	9249.7	40.9	35.3	46.9
Micronesia	6.2	4.9	7.6	80.7	63.9	99.1	7.4	5.6	9.3	59.7	45.2	75.1
Monaco	7.9	6.3	9.4	71.4	56.5	85.0	6.4	5.1	7.5	42.0	34.0	49.2
Mongolia	549.6	461.5	652.0	317.3	266.4	376.4	697.9	544.8	888.6	181.1	141.4	230.6
Montenegro	44.1	38.8	49.4	40.5	35.7	45.4	65.7	54.9	78.0	37.7	31.5	44.7
Morocco	578.4	433.6	665.6	25.5	19.2	29.4	1158.0	890.2	1371.1	21.3	16.3	25.2
Mozambique	319.4	264.1	377.2	32.5	26.9	38.4	581.7	466.9	715.3	33.5	26.9	41.2
Myanmar	2405.6	1889.4	3057.8	61.4	48.2	78.0	2445.8	2081.1	2946.9	30.9	26.3	37.2
Namibia	24.3	19.9	28.3	20.2	16.5	23.6	39.4	32.2	47.8	17.4	14.2	21.1
Nauru	0.4	0.3	0.5	70.4	55.5	87.5	0.3	0.3	0.4	51.4	40.9	64.1
Nepal	623.8	488.1	777.1	40.6	31.8	50.6	1477.9	1156.4	1864.7	39.2	30.6	49.4

Country	Later-onset gastric cancer (≥ 55 years)											
	1990						2019					
	Death number			Death rate (per 100 000)			Death number			Death rate (per 100 000)		
	Value	LUI	UUI	Value	LUI	UUI	Value	LUI	UUI	Value	LUI	UUI
Netherlands	2308.6	2172.0	2411.8	69.7	65.6	72.8	2311.3	2062.2	2527.0	40.9	36.5	44.8
New Zealand	306.7	285.9	324.9	46.6	43.4	49.4	332.9	298.6	359.8	25.2	22.6	27.3
Nicaragua	187.9	169.5	206.0	75.8	68.4	83.1	517.1	427.8	609.9	70.0	57.9	82.6
Niger	324.2	261.1	381.3	73.7	59.3	86.6	772.0	591.1	965.8	59.6	45.6	74.5
Nigeria	1616.9	1282.4	1998.9	22.4	17.8	27.7	2603.2	2101.7	3218.6	18.6	15.1	23.1
Niue	0.3	0.2	0.3	69.7	59.3	82.5	0.2	0.2	0.2	48.0	40.4	55.8
North Macedonia	341.9	312.0	366.8	104.2	95.1	111.8	426.5	340.9	534.2	74.6	59.6	93.4
Northern Mariana Islands	1.6	1.3	1.8	63.7	54.6	75.4	4.0	3.4	4.6	40.2	34.2	46.1
Norway	702.8	662.2	728.9	64.9	61.2	67.3	423.7	381.6	459.2	27.2	24.5	29.5
Oman	58.7	44.1	74.6	58.7	44.1	74.6	77.5	67.4	89.3	28.2	24.5	32.4
Pakistan	2981.3	2329.2	3553.4	32.5	25.4	38.7	4968.2	4069.4	6050.9	27.9	22.9	34.0
Palau	0.9	0.7	1.1	55.0	44.3	68.8	1.5	1.2	1.8	38.1	30.6	47.6
Palestine	73.1	56.3	92.8	50.3	38.7	63.9	112.8	96.7	130.9	28.9	24.8	33.6
Panama	204.0	188.1	217.6	83.9	77.4	89.5	372.6	293.5	470.3	53.5	42.2	67.6
Papua New Guinea	161.3	120.7	209.9	53.4	39.9	69.4	385.9	281.3	510.6	51.8	37.7	68.5
Paraguay	184.7	162.8	206.6	50.9	44.8	56.9	384.2	299.8	486.7	40.8	31.9	51.7
Peru	2448.8	2116.8	2820.2	124.4	107.5	143.2	4823.8	3653.7	6233.7	90.6	68.6	117.0
Philippines	1415.8	1257.5	1590.1	29.6	26.3	33.2	2439.8	2047.5	2881.8	18.6	15.6	22.0
Poland	7510.3	7213.0	7724.0	97.5	93.6	100.2	6141.1	5200.2	7215.2	50.9	43.1	59.8
Portugal	3378.3	3220.1	3517.4	138.4	131.9	144.1	2695.3	2415.2	2941.7	71.1	63.7	77.6
Puerto Rico	368.8	341.7	390.8	60.6	56.2	64.3	332.8	261.4	416.4	28.3	22.2	35.4
Qatar	7.2	5.7	8.9	44.4	35.3	54.8	27.4	20.6	36.4	15.8	11.9	21.1
Republic of Korea	11055.2	10550.4	11703.7	221.9	211.8	234.9	10759.2	9537.2	11942.7	67.1	59.5	74.5
Republic of Moldova	651.3	621.9	681.4	84.2	80.4	88.1	448.9	387.4	515.4	43.1	37.2	49.5
Romania	3433.8	3279.3	3586.8	68.6	65.5	71.6	3389.1	2794.4	4074.9	55.2	45.5	66.4
Russian Federation	43837.5	42540.0	44653.2	139.0	134.9	141.6	27079.1	23742.4	30747.2	63.8	55.9	72.4
Rwanda	279.5	230.2	333.6	58.5	48.2	69.9	304.3	241.5	376.0	30.3	24.1	37.4
Saint Kitts and Nevis	6.2	5.6	6.9	97.6	87.7	107.7	5.2	4.5	6.0	42.6	36.8	49.4
Saint Lucia	14.9	13.6	16.1	101.5	92.8	110.0	21.1	17.8	24.5	57.5	48.5	66.8
Saint Vincent and the Grenadines	10.0	9.1	11.1	83.2	75.4	91.8	12.4	10.8	14.1	52.1	45.4	59.4
Samoa	10.7	8.9	12.7	72.5	60.6	86.5	13.3	11.0	15.9	54.2	44.9	64.7
San Marino	8.7	7.4	10.0	154.3	130.6	177.6	12.4	8.6	16.8	122.4	85.3	166.7
Sao Tome and Principe	9.3	7.9	10.8	83.0	70.1	96.1	13.8	11.3	17.1	81.0	66.3	100.4
Saudi Arabia	301.8	226.4	380.3	33.8	25.4	42.6	383.9	315.1	462.6	14.1	11.6	17.0
Senegal	414.0	336.6	486.9	78.4	63.7	92.2	780.5	618.2	953.8	63.0	49.9	76.9
Serbia	1252.9	1059.5	1391.2	60.3	51.0	66.9	1240.1	999.7	1534.9	45.3	36.5	56.1
Seychelles	5.3	4.8	6.1	55.4	49.6	63.2	5.7	4.9	6.5	29.8	25.8	34.0
Sierra Leone	247.2	201.8	295.2	79.7	65.1	95.2	350.2	273.1	444.2	61.8	48.2	78.4
Singapore	348.7	331.0	366.3	96.2	91.3	101.0	370.7	324.6	406.8	26.7	23.3	29.3
Slovakia	915.9	867.5	961.5	88.3	83.6	92.7	687.9	547.9	845.3	42.8	34.1	52.6
Slovenia	425.5	334.3	528.8	99.3	78.0	123.4	358.5	283.8	455.3	50.2	39.8	63.8
Solomon Islands	20.2	14.4	26.0	90.4	64.3	116.0	38.9	30.5	48.3	82.8	64.9	102.6
Somalia	214.8	160.7	273.6	55.9	41.8	71.2	447.6	330.1	592.7	44.0	32.4	58.2
South Africa	1203.2	1063.9	1346.5	35.3	31.2	39.5	1934.2	1805.3	2065.5	25.6	23.9	27.3
South Sudan	177.0	133.1	226.4	46.6	35.0	59.6	188.1	137.3	253.8	30.9	22.5	41.6
Spain	7683.0	7184.6	8050.1	80.4	75.2	84.2	6677.3	5909.9	7303.7	44.5	39.4	48.7
Sri Lanka	734.5	658.9	823.3	41.1	36.9	46.1	1042.0	789.1	1363.7	22.9	17.3	29.9
Sudan	1187.4	804.4	1451.6	79.8	54.1	97.6	2054.7	1438.7	2630.4	70.6	49.4	90.4
Suriname	20.9	18.9	22.7	47.4	42.8	51.6	34.5	29.0	41.2	33.1	27.8	39.6
Sweden	1415.4	1316.4	1485.2	59.6	55.4	62.5	776.7	691.1	841.0	23.8	21.2	25.8
Switzerland	1039.3	975.2	1089.6	61.5	57.7	64.5	691.3	607.9	760.9	24.7	21.7	27.2
Syrian Arab Republic	215.9	172.4	264.6	24.4	19.5	29.9	421.0	317.0	556.7	19.5	14.7	25.8
Tajikistan	664.2	613.3	725.8	138.4	127.8	151.2	816.3	662.9	995.0	93.2	75.7	113.6
Thailand	2715.0	2380.2	3085.8	45.8	40.1	52.0	4163.1	3139.3	5435.9	23.0	17.3	30.0
Timor-Leste	18.3	13.8	22.7	42.0	31.6	52.2	48.5	36.1	59.5	35.6	26.5	43.7
Togo	145.4	121.2	172.6	74.3	62.0	88.2	340.2	273.1	429.4	58.3	46.8	73.5
Tokelau	0.2	0.1	0.2	65.7	54.1	79.6	0.1	0.1	0.1	43.0	35.1	52.5
Tonga	6.4	5.2	7.5	67.4	55.1	79.3	8.5	7.1	10.0	65.0	54.4	76.6
Trinidad and Tobago	82.3	75.9	88.7	59.9	55.3	64.6	72.3	55.7	92.7	21.6	16.7	27.7
Tunisia	265.1	218.9	318.1	30.4	25.1	36.5	531.5	396.3	714.0	24.1	18.0	32.4
Turkey	5281.9	4501.4	6122.4	86.0	73.3	99.7	7606.8	6111.8	9299.2	50.2	40.3	61.4
Turkmenistan	325.5	308.5	342.9	100.1	94.9	105.4	273.5	215.6	343.8	40.5	31.9	50.9
Tuvalu	0.9	0.7	1.1	73.8	59.8	90.1	1.0	0.8	1.2	53.8	42.3	68.0

Country	Later-onset gastric cancer (≥ 55 years)											
	1990						2019					
	Death number			Death rate (per 100 000)			Death number			Death rate (per 100 000)		
	Value	LUI	UUI	Value	LUI	UUI	Value	LUI	UUI	Value	LUI	UUI
Uganda	490.2	406.7	572.4	46.7	38.8	54.5	814.9	669.0	975.4	36.6	30.1	43.9
Ukraine	15400.0	14746.4	16021.0	123.6	118.3	128.5	8087.0	6867.1	9504.7	60.0	51.0	70.6
United Arab Emirates	29.5	23.0	36.5	55.7	43.3	68.8	134.8	100.4	175.5	24.8	18.5	32.3
United Kingdom	10482.7	10010.8	10746.3	70.3	67.1	72.0	7121.6	6483.5	7492.6	35.2	32.1	37.1
United Republic of Tanzania	821.4	688.0	964.8	45.4	38.0	53.3	1323.3	1089.1	1565.5	33.9	27.9	40.1
United States of America	17028.2	16037.3	17535.6	32.3	30.5	33.3	17237.7	15888.1	18059.6	17.9	16.5	18.7
United States Virgin Islands	7.1	6.0	8.2	50.3	42.8	58.7	16.0	13.6	18.5	48.4	41.0	56.0
Uruguay	613.4	579.0	645.3	90.9	85.8	95.6	542.0	489.9	589.4	60.9	55.1	66.3
Uzbekistan	1865.7	1760.1	1963.7	96.8	91.3	101.8	1928.9	1611.8	2250.7	52.1	43.5	60.8
Vanuatu	6.9	5.0	8.8	66.3	48.1	85.0	17.2	12.5	22.3	60.8	44.2	78.8
Venezuela	1482.8	1381.0	1565.5	94.7	88.2	99.9	2978.4	2282.9	3787.3	59.1	45.3	75.2
Viet Nam	5993.6	4796.7	7167.2	85.9	68.7	102.7	6991.8	5688.6	8305.5	43.3	35.2	51.5
Yemen	728.3	528.1	939.2	91.0	66.0	117.3	1864.8	1433.1	2436.8	88.4	67.9	115.5
Zambia	233.6	194.2	274.1	50.7	42.2	59.5	372.5	297.3	454.2	36.4	29.1	44.4
Zimbabwe	381.0	333.3	430.1	57.5	50.3	64.9	641.0	509.2	796.6	57.5	45.7	71.5

LUI, lower uncertainty intervals; UUI, upper uncertainty intervals

Table S8. Temporal trends of incidence rates of gastric cancer globally and in SDI quintiles, 1990–2019

Location	Trend 1		Trend 2		Trend 3		Trend 4	
	Years	EAPC (95% CI)	Years	EAPC (95% CI)	Years	EAPC (95% CI)	Years	EAPC (95% CI)
Global								
EOGC	1990–2002	–0.07 (–0.24 to 0.11)	2002–2011	–0.95* (–1.27 to –0.63)	2011–2016	–2.46* (–3.37 to –1.55)	2016–2019	–0.48 (–1.94 to 1.00)
IOGC	1990–1998	–2.61* (–2.81 to –2.41)	1998–2005	1.32* (1.00 to 1.64)	2005–2012	–5.00* (–5.30 to –4.70)	2012–2019	–0.39* (–0.64 to –0.14)
LOGC	1990–1998	–1.40* (–1.63 to –1.17)	1998–2004	0.71* (0.23 to 1.19)	2004–2016	–2.05* (–2.29 to –1.91)	2016–2019	–0.59 (–1.64 to 0.48)
High SDI								
EOGC	1990–1996	–1.47* (–2.31 to –0.62)	1996–2001	–3.65* (–5.19 to –2.10)	2001–2008	–1.78* (–2.62 to –0.94)	2008–2019	–2.60* (–2.93 to –2.27)
IOGC	1990–1994	–2.15* (–3.41 to –0.87)	1994–1997	–4.61* (–8.46 to –0.61)	1997–2017	–3.24* (–3.35 to –3.12)	2017–2019	0.38 (–3.67 to 4.59)
LOGC	1990–1995	–0.61* (–1.00 to –0.21)	1995–2010	–1.76* (–1.84 to –1.68)	2010–2016	–2.36* (–2.75 to –1.97)	2016–2019	–0.32 (–1.20 to 0.57)
High-middle SDI								
EOGC	1990–1994	2.72* (1.13 to 4.33)	1994–1997	–1.97 (–6.69 to 2.97)	1997–2003	1.09 (–0.02 to 2.21)	2003–2019	–1.04* (–1.23 to –0.85)
IOGC	1990–1998	–3.42* (–3.95 to –2.87)	1998–2005	2.50* (1.61 to 3.39)	2005–2012	–5.42* (–6.23 to –4.60)	2012–2019	–0.77* (–1.45 to –0.09)
LOGC	1990–1998	–1.80* (–2.14 to –1.45)	1998–2004	1.47* (0.74 to 2.22)	2004–2014	–2.63* (–2.92 to –2.34)	2014–2019	–0.79 (–1.51 to 0.07)
Middle SDI								
EOGC	1990–2003	1.10* (0.90 to 1.31)	2003–2007	–1.78 (–3.65 to 0.11)	2007–2010	1.45 (–2.36 to 5.41)	2010–2019	–2.05* (–2.39 to –1.70)
IOGC	1990–1997	–2.27* (–2.60 to –1.93)	1997–2005	2.29* (1.94 to 2.64)	2005–2012	–6.11* (–6.52 to –5.71)	2012–2019	–0.06 (–0.40 to 0.29)
LOGC	1990–1998	–1.32* (–1.63 to –1.02)	1998–2004	2.42* (1.77 to 3.07)	2004–2017	–2.04* (–2.20 to –1.87)	2017–2019	–0.68 (–3.44 to 2.17)
Low-middle SDI								
EOGC	1990–1995	–0.97* (–1.69 to –0.25)	1995–1999	0.81 (–0.82 to 2.46)	1999–2011	–0.83* (–1.05 to –0.61)	2011–2019	–1.32* (–1.66 to –0.96)
IOGC	1990–2005	–1.22* (–1.35 to –1.09)	2005–2012	–2.37* (–2.87 to –1.86)	2012–2015	1.82 (–1.25 to 4.98)	2015–2019	–1.00* (–1.95 to –0.03)
LOGC	1990–1996	–1.01* (–1.25 to –0.78)	1996–2004	0.24* (0.05 to 0.43)	2004–2014	–1.32* (–1.45 to –1.19)	2014–2019	–0.55* (–0.87 to –0.24)
Low SDI								
EOGC	1990–1999	–1.07* (–1.14 to –0.99)	1999–2013	–1.32* (–1.37 to –1.28)	2013–2016	–1.68* (–2.51 to –0.84)	2016–2019	–0.90* (–1.32 to –0.47)
IOGC	1990–1997	–0.69* (–0.89 to –0.49)	1997–2011	–1.83* (–1.91 to –1.75)	2011–2014	0.15 (–1.37 to 1.70)	2014–2019	–0.79* (–1.13 to –0.45)
LOGC	1990–2000	–0.77* (–0.83 to –0.70)	2000–2003	–0.42 (–1.25 to 0.42)	2003–2016	–1.24* (–1.29 to –1.19)	2016–2019	–0.83* (–1.24 to –0.41)

CI, confidence interval; EAPC, estimated annual percentage change; GC, gastric cancer; SDI, sociodemographic index; EOGC, early-onset gastric cancer (15–49 years); IOGC, intermediate-onset gastric cancer (50–54 years); LOGC, later-onset gastric cancer (≥ 55 years); *Significantly different from 0 (*P* < .05).

Table S9. Temporal trends of death rates of gastric cancer globally and in SDI quintiles, 1990–2019

Location	Trend 1		Trend 2		Trend 3		Trend 4	
	Years	EAPC (95% CI)	Years	EAPC (95% CI)	Years	EAPC (95% CI)	Years	EAPC (95% CI)
Global								
EOGC	1990–2002	–0.42* (–0.58 to –0.25)	2002–2011	–2.49* (–2.79 to –2.18)	2011–2015	–3.33* (–4.71 to –1.93)	2015–2019	–1.78* (–2.67 to –0.88)
IOGC	1990–1998	–3.02* (–3.21 to –2.83)	1998–2005	0.70* (0.39 to 1.01)	2005–2011	–6.55* (–6.93 to –6.17)	2011–2019	–1.65* (–1.85 to –1.46)
LOGC	1990–1998	–1.66* (–1.85 to –1.46)	1998–2004	0.28 (–0.13 to 0.70)	2004–2015	–2.92* (–3.06 to –2.79)	2015–2019	–1.35* (–1.92 to –0.78)
High SDI								
EOGC	1990–1996	–2.42* (–2.87 to –1.96)	1996–2000	–4.27* (–5.58 to –2.94)	2000–2016	–3.39* (–3.50 to –3.27)	2016–2019	–0.63 (–1.99 to –0.75)
IOGC	1990–1994	–3.47* (–4.25 to –2.69)	1994–1997	–6.06* (–8.42 to –3.63)	1997–2016	–3.71* (–3.79 to –3.63)	2016–2019	–0.77 (–2.03 to 0.50)
LOGC	1990–1999	–1.76* (–1.86 to –1.67)	1999–2006	–2.54* (–2.72 to –2.36)	2006–2016	–2.28* (–2.38 to –2.18)	2016–2019	0.01 (–0.54 to 0.56)
High-middle SDI								
EOGC	1990–1994	1.78* (0.08 to 3.52)	1994–2004	–0.96* (–1.44 to –0.48)	2004–2014	–3.69 (–4.15 to –3.22)	2014–2019	–1.51* (–2.68 to –0.32)
IOGC	1990–1998	–3.80* (–4.42 to –3.18)	1998–2005	1.24* (0.23 to 2.25)	2005–2011	–7.62* (–8.83 to –6.39)	2011–2019	–2.61* (–3.24 to –1.98)
LOGC	1990–1998	–2.08* (–2.44 to –1.72)	1998–2004	0.63 (–0.12 to 1.39)	2004–2013	–3.91* (–4.26 to –3.55)	2013–2019	–1.80* (–2.35 to –1.24)
Middle SDI								
EOGC	1990–2002	0.45* (0.25 to 0.66)	2002–2007	–3.09* (–4.15 to –2.01)	2007–2010	–1.21 (–4.60 to 2.31)	2010–2019	–3.42* (–3.73 to –3.11)
IOGC	1990–1997	–2.72* (–3.01 to –2.42)	1997–2005	1.23* (0.93 to 1.54)	2005–2011	–8.04* (–8.50 to –7.58)	2011–2019	–2.14* (–2.38 to –1.90)
LOGC	1990–1998	–1.50* (–1.72 to –1.27)	1998–2004	1.73* (1.26 to 2.20)	2004–2015	–3.36* (–3.51 to –3.20)	2015–2019	–2.45* (–3.09 to –1.81)
Low-middle SDI								
EOGC	1990–1995	–1.08* (–1.72 to –0.44)	1995–1999	0.69 (–0.76 to 2.16)	1999–2010	–1.11* (–1.33 to –0.88)	2010–2019	–1.55* (–1.82 to –1.29)
IOGC	1990–2005	–1.41* (–1.54 to –1.28)	2005–2012	–2.68* (–3.20 to –2.16)	2012–2015	1.53 (–1.63 to 4.79)	2015–2019	–1.71* (–2.69 to –0.72)
LOGC	1990–1996	–1.03* (–1.26 to –0.80)	1996–2004	0.18 (–0.00 to 0.36)	2004–2014	–1.57* (–1.70 to –1.45)	2014–2019	–0.83* (–1.13 to –0.53)
Low SDI								
EOGC	1990–1999	–1.10* (–1.18 to –1.03)	1999–2013	–1.32* (–1.37 to –1.28)	2013–2016	–1.68* (–2.54 to –0.81)	2016–2019	–0.97* (–1.40 to –0.54)
IOGC	1990–1997	–0.71* (–0.91 to –0.50)	1997–2011	–1.88* (–1.96 to –1.80)	2011–2014	0.08 (–1.44 to 1.63)	2014–2019	–0.85* (–1.19 to –0.51)
LOGC	1990–2000	–0.72* (–0.79 to –0.66)	2000–2003	–0.38 (–1.23 to 0.49)	2003–2017	–1.23* (–1.27 to –1.19)	2017–2019	–0.68 (–1.54 to 0.18)

CI, confidence interval; EAPC, estimated annual percentage change; GC, gastric cancer; SDI, sociodemographic index; EOGC, early-onset gastric cancer (15–49 years); IOGC, intermediate-onset gastric cancer (50–54 years); LOGC, later-onset gastric cancer (≥ 55 years); *Significantly different from 0 (*P* < .05).

Table S10. Temporal trends of incidence rates of gastric cancer in 21 GBD regions, 1990–2019

Location	Trend 1		Trend 2		Trend 3		Trend 4	
	**Years**	**EAPC (95% CI)**	**Years**	**EAPC (95% CI)**	**Years**	**EAPC (95% CI)**	**Years**	**EAPC (95% CI)**
Andean Latin America								
EOGC	1990–1994	1.18 (–0.77 to 3.17)	1994–2011	–0.23* (–0.47 to –0.01)	2011–2015	–3.94* (–6.86 to –0.94)	2015–2019	0.31 (–1.62 to 2.28)
IOGC	1990–2007	–1.53* (–1.66 to –1.41)	2007–2010	1.37 (–2.28 to 5.15)	2010–2015	–4.17* (–5.27 to –3.05)	2015–2019	–0.99 (–2.13 to 0.16)
LOGC	1990–2003	–0.41* (–0.63 to –0.20)	2003–2007	–1.63 (–3.65 to 0.42)	2007–2010	1.10 (–3.00 to 5.37)	2010–2019	–1.75* (–2.12 to –1.37)
Australasia								
EOGC	1990–2009	0.04 (–0.11 to 0.19)	2009–2012	–2.64 (–7.57 to 2.55)	2012–2015	1.86 (–3.30 to 7.29)	2015–2019	–1.82* (–3.42 to –0.19)
IOGC	1990–1997	–1.64* (–2.21 to –1.07)	1997–2005	–2.37* (–2.94 to –1.80)	2005–2016	–0.20 (–0.54 to 0.14)	2016–2019	–1.90 (–4.01 to 0.26)
LOGC	1990–1999	–0.62* (–0.92 to –0.32)	1999–2006	–2.36* (–2.91 to –1.82)	2006–2013	–1.67* (–2.21 to –1.12)	2013–2019	–0.08 (–0.63 to 0.48)
Caribbean								
EOGC	1990–2008	–0.07 (–0.14 to 0.01)	2008–2011	–1.16 (–3.40 to 1.13)	2011–2015	1.63* (0.47 to 2.80)	2015–2019	–0.28 (–1.00 to 0.44)
IOGC	1990–1997	–2.26* (–2.68 to –1.85)	1997–2008	–0.02 (–0.27 to 0.22)	2008–2011	–1.80 (–4.86 to 1.35)	2011–2019	–0.42* (–0.76 to –0.08)
LOGC	1990–1995	–1.97* (–2.65 to –1.30)	1995–2003	–1.26* (–1.67 to –0.85)	2003–2012	–0.71* (–1.04 to –0.37)	2003–2019	–0.20 (–0.61 to 0.21)
Central Asia								
EOGC	1990–1996	1.76* (0.95 to 2.58)	1996–1999	–5.17* (–9.54 to –0.59)	1999–2015	–2.55* (–2.74 to –2.35)	2015–2019	0.19 (–1.29 to 1.70)
IOGC	1990–1998	–3.92* (–4.23 to –3.60)	1998–2001	–1.76 (–4.68 to 1.25)	2001–2015	–2.85* (–3.01 to –2.70)	2015–2019	–0.98* (–1.92 to –0.03)
LOGC	1990–1994	–1.00* (–1.58 to –0.41)	1994–1999	–3.29* (–3.86 to –2.72)	1999–2004	0.41* (–0.81 to 1.01)	2004–2019	–2.40* (–2.48 to –2.33)
Central Europe								
EOGC	1990–1997	0.04 (–0.25 to 0.33)	1997–2008	–2.26* (–2.43 to –2.09)	2008–2013	–3.60* (–4.26 to –2.93)	2013–2019	0.60* (0.23 to 0.97)
IOGC	1990–2000	–2.13* (–2.32 to –1.94)	2000–2008	–0.71* (–0.47 to –0.01)	2008–2016	–3.48* (–3.80 to –3.16)	2016–2019	–0.31 (–1.55 to 0.94)
LOGC	1990–1993	–2.13* (–3.20 to –1.05)	1993–2004	–1.27* (–1.44 to –1.11)	2004–2013	–2.49* (–2.73 to –2.26)	2013–2019	–0.81* (–1.18 to –0.44)
Central Latin America								
EOGC	1990–2002	0.79* (0.65 to 0.94)	2002–2009	–0.05 (–0.46 to 0.37)	2009–2012	–1.35 (–3.75 to 1.10)	2012–2019	0.94* (0.61 to 1.27)
IOGC	1990–1999	–1.62* (–1.98 to –1.25)	1999–2002	0.66 (–3.33 to 4.82)	2002–2014	–1.55* (–1.82 to –1.28)	2014–2019	–0.18 (–1.08 to 0.73)
LOGC	1990–1993	–0.15 (–1.60 to 1.33)	1993–2003	–1.34* (–1.61 to –1.08)	2003–2014	–2.35* (–2.57 to –2.12)	2014–2019	0.13 (–0.52 to 0.79)
Central sub-Saharan Africa								
EOGC	1990–2000	–0.88* (–1.06 to –0.69)	2000–2007	–2.05* (–2.44 to –1.66)	2007–2011	–1.25* (–2.41 to –0.08)	2011–2019	–1.86* (–2.11 to –1.61)
IOGC	1990–1999	–1.07* (–1.38 to –0.75)	1999–2006	–2.12* (–2.70 to –1.54)	2006–2009	–0.33 (–3.75 to 3.21)	2009–2019	–2.04* (–2.31 to –1.77)
LOGC	1990–1997	–0.43* (–0.67 to –0.19)	1997–2013	–1.58* (–1.65 to –1.50)	2013–2016	–2.54* (–4.28 to –0.77)	2016–2019	–0.98* (–1.87 to –0.09)
East Asia								
EOGC	1990–1993	–0.04 (–2.62 to 2.61)	1993–2002	2.96* (2.38 to 3.55)	2002–2010	0.48 (–0.22 to 1.18)	2010–2019	–0.85* (–1.32 to –0.37)
IOGC	1990–1997	–2.49* (–2.99 to –1.99)	1997–2005	3.03* (2.50 to 3.56)	2005–2014	–5.00* (–5.40 to –4.60)	2014–2019	–0.35 (–1.20 to 0.52)

Location	Trend 1		Trend 2		Trend 3		Trend 4	
	Years	EAPC (95% CI)	Years	EAPC (95% CI)	Years	EAPC (95% CI)	Years	EAPC (95% CI)
LOGC	1990–1998	–1.32* (–1.58 to –1.05)	1998–2004	4.20* (3.62 to 4.78)	2004–2015	–2.47* (–2.65 to –2.28)	2015–2019	–0.88* (–1.66 to –0.10)
Eastern Europe								
EOGC	1990–1994	8.93* (4.20 to 13.87)	1994–1997	–6.80 (–19.01 to 7.25)	1997–2013	–2.99* (–3.56 to –2.41)	2013–2019	0.57 (–1.79 to 2.98)
IOGC	1990–1994	4.22 (–1.09 to 9.82)	1994–1997	–8.74 (–22.65 to 7.68)	1997–2013	–3.54* (–4.22 to –2.87)	2013–2019	–1.32 (–4.04 to 1.48)
LOGC	1990–1994	2.28 (–0.62 to 5.26)	1994–1997	–6.92 (–15.01 to 1.93)	1997–2000	0.62 (–8.12 to 10.19)	2000–2019	–2.99* (–3.25 to –2.73)
Eastern sub-Saharan Africa								
EOGC	1990–1998	–1.15* (–1.32 to –0.99)	1998–2001	–3.82* (–5.28 to –2.34)	2001–2011	–2.58* (–2.71 to –2.44)	2011–2019	–0.89* (–1.06 to –0.73)
IOGC	1990–1997	–0.83* (–1.02 to –0.65)	1997–2001	–2.70* (–3.38 to –2.01)	2001–2016	–2.40* (–2.46 to –2.34)	2016–2019	–0.94* (–1.63 to –0.24)
LOGC	1990–1998	–0.65* (–0.78 to –0.52)	1998–2005	–1.55* (–1.75 to –1.35)	2005–2015	–1.81* (–1.92 to –1.70)	2015–2019	–0.92* (–1.30 to –0.54)
High-income Asia Pacific								
EOGC	1990–1997	–2.31* (–3.36 to –1.24)	1997–2001	–5.98* (–9.72 to –2.10)	2001–2008	–1.74* (–3.08 to –0.39)	2008–2019	–3.28 (–3.81 to –2.75)
IOGC	1990–2000	–3.65* (–3.98 to –3.32)	2000–2010	–3.07* (–3.46 to –2.69)	2010–2016	–6.19* (–7.10 to –5.27)	2016–2019	–1.34 (–3.47 to 0.83)
LOGC	1990–1994	–0.94 (–2.09 to 0.22)	1994–2002	–2.41* (–2.89 to –1.93)	2002–2010	–1.44* (–1.92 to –0.95)	2010–2019	–1.94* (–2.27 to –1.61)
High-income North America								
EOGC	1990–1994	2.91* (1.68 to 4.16)	1994–2003	0.67* (0.25 to 1.09)	2003–2013	–1.12* (–1.46 to –0.77)	2013–2019	–0.60 (–1.24 to 0.04)
IOGC	1990–1995	–0.50 (–1.05 to 0.04)	1995–1998	–2.97* (–5.32 to –0.57)	1998–2002	–0.08 (–1.30 to 1.15)	2002–2019	–0.61* (–0.69 to –0.52)
LOGC	1990–1999	–0.70* (–1.00 to –0.39)	1999–2007	–2.92* (–3.35 to –2.49)	2007–2015	–1.71* (–2.15 to –1.27)	2015–2019	0.41 (–0.64 to 1.47)
North Africa and Middle East								
EOGC	1990–1994	–1.36* (–1.67 to –1.04)	1994–2003	–2.73* (–2.84 to –2.62)	2003–2007	–0.14 (–0.65 to 0.38)	2007–2019	0.73* (0.67 to 0.79)
IOGC	1990–2000	–3.09* (–3.21 to –2.97)	2000–2003	–0.93 (–2.49 to 0.66)	2003–2007	–2.32* (–3.09 to –1.54)	2007–2019	–0.16* (–0.26 to –0.07)
LOGC	1990–1995	–1.43* (–1.88 to –0.98)	1995–2001	–2.46* (–2.91 to –2.02)	2001–2015	–0.16* (–0.27 to –0.05)	2015–2019	–1.29* (–1.92 to 0.65)
Oceania								
EOGC	1990–1992	0.76 (–1.09 to 2.65)	1992–1995	–0.97 (–2.79 to 0.88)	1995–2007	1.01* (0.88 to 1.13)	2007–2019	–0.27* (–0.38 to –0.16)
IOGC	1990–1992	0.15 (–1.38 to 1.69)	1992–1995	–1.29 (–2.79 to –0.24)	1995–2011	0.07* (0.00 to 0.13)	2011–2019	–0.78* (–0.95 to –0.61)
LOGC	1990–1992	1.31* (0.22 to 2.41)	1992–1999	0.12 (–0.07 to 0.30)	1999–2011	–0.54* (–0.61 to –0.47)	2011–2019	–0.73* (–0.84 to –0.61)
South Asia								
EOGC	1990–2001	–0.51* (–0.84 to –0.19)	2001–2004	–2.63 (–7.25 to 2.22)	2004–2010	0.17 (–0.92 to 1.26)	2010–2019	–1.28* (–1.71 to –0.84)
IOGC	1990–1997	–0.01 (–0.90 to 0.89)	1997–2006	–3.40* (–4.10 to –2.69)	2006–2016	0.98* (0.37 to 1.60)	2016–2019	–2.57 (–5.78 to 0.75)
LOGC	1990–1995	–1.58* (–2.46 to –0.69)	1995–1998	0.85 (–3.10 to 4.97)	1998–2005	–2.03* (–2.69 to –1.37)	2005–2019	–0.89* (–1.08 to 0.70)
Southeast Asia								
EOGC	1990–2002	–0.16* (–0.26 to –0.06)	2002–2006	–2.66* (–3.48 to –1.83)	2006–2014	–1.99* (–2.21 to –1.77)	2014–2019	–0.63* (–1.01 to –0.25)
IOGC	1990–2004	–2.02* (–2.10 to –1.94)	2004–2009	–2.60* (–3.14 to –2.07)	2009–2015	–1.73* (–2.11 to –1.35)	2015–2019	–1.28* (–1.82 to –0.74)
LOGC	1990–1998	–1.13* (–1.29 to –0.98)	1998–2009	–2.49* (–2.60 to –2.38)	2009–2015	–1.69* (–2.00 to –1.37)	2015–2019	–0.39 (–0.84 to 0.06)

Location	Trend 1		Trend 2		Trend 3		Trend 4	
	**Years**	**EAPC (95% CI)**	**Years**	**EAPC (95% CI)**	**Years**	**EAPC (95% CI)**	**Years**	**EAPC (95% CI)**
Southern Latin America								
EOGC	1990–1992	–3.80 (–7.47 to 0.00)	1992–1997	0.73 (–0.50 to 1.97)	1997–2015	–1.17* (–1.30 to –1.03)	2015–2019	0.22 (–1.01 to 1.46)
IOGC	1990–1992	–4.90* (–8.51 to –1.15)	1992–2003	–0.74* (–1.04 to –0.44)	2003–2015	–1.66* (–1.91 to –1.40)	2015–2019	–0.98 (–2.18 to 0.24)
LOGC	1990–1992	–2.09 (–5.23 to 1.16)	1992–1999	–0.29 (–0.84 to 0.26)	1999–2014	–1.56* (–1.71 to –1.41)	2014–2019	–0.27 (–0.99 to 0.46)
Southern sub-Saharan Africa								
EOGC	1990–1995	–2.02 (–4.09 to 0.11)	1995–2003	0.76 (–0.52 to 2.06)	2003–2016	–3.57* (–4.12 to –3.03)	2016–2019	3.33* (–1.50 to 8.40)
IOGC	1990–1994	–0.86 (–2.90 to 1.22)	1994–1998	5.48* (2.08 to 8.99)	1998–2016	–2.29 (–2.51 to –2.06)	2016–2019	–5.76* (–8.80 to –2.62)
LOGC	1990–1994	0.91 (–0.59 to 2.42)	1994–1997	7.13* (2.19 to 12.31)	1997–2017	–1.99* (–2.13 to –1.86)	2017–2019	–4.38 (–8.80 to 0.24)
Tropical Latin America								
EOGC	1990–1992	–1.44 (–4.07 to 1.25)	1992–1999	0.50* (0.04 to 0.96)	1999–2009	–0.85* (–1.09 to –0.61)	2009–2019	–1.43* (–1.64 to –1.23)
IOGC	1990–1992	–4.01* (–6.21 to –1.77)	1992–2009	–1.76* (–1.85 to –1.68)	2009–2014	–3.14* (–3.85 to –2.43)	2014–2019	–1.62 (–2.13 to –1.11)
LOGC	1990–2003	–1.26* (–1.37 to –1.16)	2003–2007	–3.17* (–4.16 to –2.17)	2007–2014	–2.41* (–2.75 to –2.07)	2014–2019	–1.39* (–1.85 to –0.94)
Western Europe								
EOGC	1990–2003	–0.26* (–0.37 to –0.15)	2003–2009	–0.84* (–1.30 to –0.38)	2009–2016	–2.48* (–2.82 to –2.13)	2016–2019	–0.16 (–1.20 to 0.89)
IOGC	1990–1999	–2.10* (–2.30 to –1.90)	1999–2010	–1.15* (–1.32 to –0.97)	2010–2014	–2.59* (–3.69 to –1.47)	2014–2019	–1.20* (–1.70 to –0.69)
LOGC	1990–1997	–1.97* (–2.29 to –1.65)	1997–2000	1.22 (–3.63 to 1.25)	2000–2014	–1.95* (–2.08 to –1.82)	2014–2019	–0.31 (–0.86 to 0.24)
Western sub-Saharan Africa								
EOGC	1990–1999	–1.55* (–1.66 to –1.44)	1999–2009	–1.08* (–1.19 to –0.97)	2009–2014	–0.34 (–0.72 to 0.05)	2014–2019	–1.20* (–1.47 to –0.93)
IOGC	1990–1993	–1.74* (–2.56 to –0.92)	1993–2002	–1.21* (–1.39 to –1.03)	2002–2014	–0.83* (–0.94 to –0.72)	2014–2019	–1.75* (–2.12 to –1.38)
LOGC	1990–1999	–1.15* (–1.24 to –1.07)	1999–2004	0.54* (0.24 to 0.83)	2004–2013	–0.25* (–0.35 to –0.15)	2013–2019	–1.48* (–1.63 to –1.33)

CI, confidence interval; EAPC, estimated annual percentage change; GC, gastric cancer; EOGC, early-onset gastric cancer (15–49 years); IOGC, intermediate-onset gastric cancer (50–54 years); LOGC, later-onset gastric cancer (≥ 55 years). *Significantly different from 0 ($P < .05$).

Supplementary Table S11 Temporal Trends of Death Rates of Gastric Cancer in 21 GBD Regions, 1990–2019

Location	Trend 1		Trend 2		Trend 3		Trend 4	
	**Years**	**EAPC (95% CI)**	**Years**	**EAPC (95% CI)**	**Years**	**EAPC (95% CI)**	**Years**	**EAPC (95% CI)**
Andean Latin America								
EOGC	1990–1994	1.05 (–0.87 to 3.01)	1994–2011	–0.61* (–0.84 to –0.37)	2011–2015	–4.38* (–7.23 to –1.43)	2015–2019	–0.15 (–2.05 to 1.78)
IOGC	1990–2007	–1.69* (–1.82 to –1.57)	2007–2010	1.19 (–2.41 to 4.92)	2010–2015	–4.37* (–5.46 to –3.27)	2015–2019	–1.15* (–2.27 to –0.01)
LOGC	1990–1998	–0.38 (–0.82 to 0.06)	1998–2007	–1.31* (–1.74 to –0.88)	2007–2010	0.41 (–3.55 to 4.54)	2010–2019	–2.23* (–2.59 to –1.87)
Australasia								
EOGC	1990–2009	–0.97* (–1.08 to –0.86)	2009–2012	–3.36 (–6.89 to 0.30)	2012–2015	0.20 (–3.46 to 3.99)	2015–2019	–2.04* (–3.19 to –0.88)
IOGC	1990–2005	–3.36* (–3.47 to –3.24)	2005–2009	–0.46 (–1.85 to 0.95)	2009–2016	–0.93* (–1.40 to 0.46)	2016–2019	–1.83* (–3.20 to –0.44)
LOGC	1990–2002	–2.10* (–2.25 to –1.94)	2002–2005	–4.40* (–6.98 to –1.75)	2005–2013	–2.08* (–2.44 to –1.72)	2013–2019	–0.31 (–0.77 to 0.15)
Caribbean								
EOGC	1990–2008	–0.27* (–0.35 to –0.20)	2008–2011	–1.25 (–3.43 to 0.98)	2011–2015	1.78* (0.66 to 2.93)	2015–2019	–0.39 (–1.09 to 0.31)
IOGC	1990–1997	–2.50* (–2.93 to –2.08)	1997–2008	–0.29* (–0.54 to 0.03)	2008–2011	–1.84 (–4.98 to 1.41)	2011–2019	–0.61* (–0.96 to –0.26)
LOGC	1990–2002	–1.76* (–1.94 to –1.59)	2002–2012	–0.98* (–1.25 to –0.70)	2012–2017	–0.01 (–0.96 to 0.94)	2017–2019	–1.95 (–4.84 to 1.03)
Central Asia								
EOGC	1990–1996	1.97* (1.12 to 2.83)	1996–1999	–5.08* (–9.67 to –0.25)	1999–2016	–2.64* (–2.83 to –2.46)	2016–2019	0.83 (–1.64 to 3.36)
IOGC	1990–1998	–3.87* (–4.18 to –3.55)	1998–2001	–1.81 (–4.70 to 1.17)	2001–2015	–3.00* (–3.15 to –2.85)	2015–2019	–1.21* (–2.14 to –0.27)
LOGC	1990–1994	–0.88* (–1.42 to –0.34)	1994–1998	–3.66* (–4.48 to –2.83)	1998–2005	–0.05 (–0.34 to 0.24)	2005–2019	–2.75* (–2.83 to –2.67)
Central Europe								
EOGC	1990–1997	–0.32* (–0.62 to –0.02)	1997–2007	–2.78* (–2.98 to –2.58)	2007–2013	–4.04* (–4.52 to –3.55)	2013–2019	0.37 (–0.01 to 0.75)
IOGC	1990–2000	–2.52* (–2.71 to –2.33)	2000–2008	–1.12* (–1.45 to –0.79)	2008–2016	–3.88* (–4.20 to –3.56)	2016–2019	–0.39 (–1.63 to 0.86)
LOGC	1990–1993	–2.37* (–3.43 to –1.31)	1993–2005	–1.62* (–1.76 to –1.48)	2005–2013	–2.88* (–3.16 to –2.60)	2013–2019	–0.91* (–1.27 to –0.55)
Central Latin America								
EOGC	1990–2002	0.07 (–0.08 to 0.23)	2002–2009	–0.57* (–1.00 to –0.13)	2009–2012	–2.10 (–4.60 to 0.47)	2012–2019	0.43* (0.08 to 0.78)
IOGC	1990–1999	–2.16* (–2.54 to –1.77)	1999–2002	0.25 (–3.99 to 4.67)	2002–2014	–1.98* (–2.26 to –1.69)	2014–2019	–0.61 (–1.56 to 0.36)
LOGC	1990–1992	0.05 (–3.02 to 3.22)	1992–2003	–1.74* (–1.97 to –1.50)	2003–2014	–2.55* (–2.78 to –2.31)	2014–2019	–0.33 (–1.03 to 0.36)
Central sub-Saharan Africa								
EOGC	1990–2000	–0.81* (–0.99 to –0.63)	2000–2007	–2.03* (–2.42 to –1.65)	2007–2011	–1.21* (–2.36 to –0.06)	2011–2019	–1.89* (–2.14 to –1.64)
IOGC	1990–1996	–0.81* (–1.24 to –0.38)	1996–2007	–1.82* (–2.02 to –1.63)	2007–2011	–1.02 (–2.29 to 0.27)	2011–2019	–2.22* (–2.50 to –1.95)
LOGC	1990–1997	–0.29* (–0.49 to –0.10)	1997–2013	–1.59* (–1.65 to –1.53)	2013–2016	–2.70* (–4.14 to –1.24)	2016–2019	–1.07* (–1.80 to –0.33)
East Asia								
EOGC	1990–1993	–0.57 (–3.01 to 1.93)	1993–2002	1.98* (1.43 to 2.54)	2002–2011	–2.48* (–3.00 to –1.95)	2011–2019	–3.30* (–3.82 to –2.77)
IOGC	1990–1997	–3.02* (–3.57 to –2.47)	1997–2005	1.64* (1.07 to 2.22)	2005–2013	–7.48* (–8.00 to –6.96)	2013–2019	–2.54* (–3.23 to –1.84)

Location	Trend 1		Trend 2		Trend 3		Trend 4	
	Years	EAPC (95% CI)	Years	EAPC (95% CI)	Years	EAPC (95% CI)	Years	EAPC (95% CI)
LOGC	1990–1998	–1.63* (–1.89 to –1.37)	1998–2004	3.33* (2.77 to 3.89)	2004–2013	–4.23* (–4.48 to –3.98)	2013–2019	–2.59* (–2.98 to –2.19)
Eastern Europe								
EOGC	1990–1994	7.15* (2.43 to 12.08)	1994–2005	–3.67* (–4.73 to –2.60)	2005–2012	–6.08* (–8.31 to –3.79)	2012–2019	0.06 (–1.82 to 1.98)
IOGC	1990–1993	3.29 (–5.81 to 13.28)	1993–2005	–4.00* (–5.19 to –2.80)	2005–2012	–6.16* (–9.04 to –3.18)	2012–2019	–1.34 (–3.74 to 1.13)
LOGC	1990–1994	2.37 (–0.76 to 5.59)	1994–1997	–6.71 (–15.43 to 2.91)	1997–2001	–0.15 (–4.93 to 4.87)	2001–2019	–3.69* (–4.00 to –3.39)
Eastern sub-Saharan Africa								
EOGC	1990–1998	–1.24* (–1.41 to –1.08)	1998–2002	–3.56* (–4.29 to –2.81)	2002–2012	–2.23* (–2.36 to –2.09)	2012–2019	–0.79* (–0.99 to –0.58)
IOGC	1990–1997	–0.86* (–1.03 to –0.70)	1997–2001	–2.70* (–3.29 to –2.09)	2001–2016	–2.32* (–2.38 to –2.27)	2016–2019	–1.34* (–1.95 to –0.73)
LOGC	1990–1998	–0.59* (–0.73 to –0.44)	1998–2012	–1.55* (–1.62 to –1.48)	2012–2015	–2.00* (–3.32 to –0.66)	2015–2019	–0.94* (–1.36 to –0.51)
High-income Asia Pacific								
EOGC	1990–1997	–3.34* (–3.97 to –2.71)	1997–2001	–6.83* (–9.07 to –4.54)	2001–2017	–4.26* (–4.46 to –4.06)	2017–2019	1.97 (–2.87 to 7.05)
IOGC	1990–2000	–4.99* (–5.29 to –4.70)	2000–2011	–4.14* (–4.43 to –3.84)	2011–2016	–7.15* (–8.32 to –5.97)	2016–2019	–1.08 (–3.03 to 0.91)
LOGC	1990–1995	–1.52* (–2.02 to 1.01)	1995–2003	–2.74* (–3.04 to –2.44)	2003–2017	–1.80* (–1.92 to –1.68)	2017–2019	0.80 (–1.50 to 3.16)
High-income North America								
EOGC	1990–1995	0.74 (–0.03 to 1.51)	1995–2006	–0.49* (–0.76 to –0.23)	2006–2012	–2.40* (–3.15 to –1.65)	2012–2019	–0.59* (–1.05 to –0.14)
IOGC	1990–1995	–2.22* (–2.68 to –1.75)	1995–1998	–3.84* (–5.88 to –1.77)	1998–2013	–1.18* (–1.28 to –1.08)	2013–2019	–0.66* (–1.02 to –0.30)
LOGC	1990–1999	–1.77* (–1.97 to –1.57)	1999–2010	–3.19* (–3.36 to –3.02)	2010–2017	–1.36* (–1.74 to –0.98)	2017–2019	1.81 (–0.47 to 4.14)
North Africa and Middle East								
EOGC	1990–1994	–1.42* (–1.87 to –0.97)	1994–2002	–3.12* (–3.31 to –2.94)	2002–2005	–1.60* (–3.00 to –0.17)	2005–2019	0.24* (0.18 to 0.31)
IOGC	1990–2000	–3.24* (–3.36 to –3.11)	2000–2003	–1.40 (–3.00 to 0.22)	2003–2007	–2.70* (–3.49 to –1.90)	2007–2019	–0.53* (–0.62 to –0.43)
LOGC	1990–1995	–1.47* (–1.91 to –1.03)	1995–2001	–2.48* (–2.91 to –2.04)	2001–2014	–0.37* (–0.49 to –0.25)	2014–2019	–1.64* (–2.07 to –1.20)
Oceania								
EOGC	1990–1996	–0.33* (–0.62 to –0.05)	1996–2007	1.05* (0.92 to 1.19)	2007–2011	0.01 (–0.83 to 0.86)	2011–2019	–0.44* (–0.62 to –0.25)
IOGC	1990–1997	–0.71* (–0.92 to –0.50)	1997–2011	0.10* (0.02 to 0.18)	2011–2017	–0.96* (–1.31 to –0.61)	2017–2019	–0.14 (–1.72 to 1.45)
LOGC	1990–1992	1.43* (0.42 to 2.44)	1992–1999	0.20* (0.04 to 0.37)	1999–2011	–0.58* (–0.64 to –0.51)	2011–2019	–0.85* (–0.95 to –0.74)
South Asia								
EOGC	1990–2001	–0.56* (–0.86 to –0.25)	2001–2004	–2.88 (–7.22 to 1.68)	2004–2008	0.66 (–1.62 to 2.99)	2008–2019	–1.21* (–1.52 to –0.91)
IOGC	1990–1997	–0.03 (–0.93 to 0.87)	1997–2006	–3.45* (–4.16 to –2.74)	2006–2016	0.86* (0.25 to 1.49)	2016–2019	–2.67 (–5.89 to 0.66)
LOGC	1990–1995	–1.53* (–2.37 to –0.69)	1995–1998	0.51 (–3.26 to 4.42)	1998–2011	–1.57* (–1.80 to –1.35)	2005–2019	–0.53* (–0.94 to –0.11)
Southeast Asia								
EOGC	1990–2002	–0.38* (–0.48 to –0.28)	2002–2006	–2.91* (–3.71 to –2.09)	2006–2014	–2.38* (–2.60 to –2.16)	2014–2019	–1.16* (–1.53 to –0.79)
IOGC	1990–2004	–2.25* (–2.32 to –2.17)	2004–2009	–2.93* (–3.45 to –2.40)	2009–2015	–2.11* (–2.49 to –1.74)	2015–2019	–1.70* (–2.23 to –1.17)
LOGC	1990–1998	–1.18* (–1.33 to –1.03)	1998–2010	–2.57* (–2.67 to –2.48)	2010–2015	–1.89* (–2.33 to –1.45)	2015–2019	–0.85* (–1.30 to –0.41)

Location	Trend 1		Trend 2		Trend 3		Trend 4	
	Years	EAPC (95% CI)	Years	EAPC (95% CI)	Years	EAPC (95% CI)	Years	EAPC (95% CI)
Southern Latin America								
EOGC	1990–1992	–3.85* (–6.64 to –0.97)	1992–1998	–0.28 (–0.93 to 0.38)	1998–2015	–1.74* (–1.86 to –1.63)	2015–2019	–0.31 (–1.23 to 0.62)
IOGC	1990–1993	–3.75* (–5.41 to –2.06)	1993–2004	–1.14* (–1.40 to –0.87)	2004–2015	–2.17* (–2.44 to –1.91)	2015–2019	–1.39* (–2.47 to –0.30)
LOGC	1990–2003	–0.92* (–1.07 to –0.77)	2003–2006	–2.96* (–5.72 to –0.12)	2006–2015	–1.62* (–1.93 to –1.31)	2015–2019	–0.57 (–1.48 to 0.34)
Southern sub-Saharan Africa								
EOGC	1990–1995	–2.19* (–4.26 to –0.08)	1995–2003	0.41 (–0.86 to 1.70)	2003–2016	–3.35* (–3.90 to –2.81)	2016–2019	3.10 (–1.71 to 8.14)
IOGC	1990–1994	–1.08 (–3.20 to 1.09)	1994–1998	5.42* (1.86 to 9.10)	1998–2016	–2.25* (–2.48 to –2.01)	2016–2019	–5.98* (–9.16 to –2.70)
LOGC	1990–1994	0.81 (–0.73 to 2.38)	1994–1997	7.16* (2.05 to 12.52)	1997–2017	–2.05* (–2.20 to –1.91)	2017–2019	–4.35 (–8.91 to 0.43)
Tropical Latin America								
EOGC	1990–1992	–1.62 (–4.13 to 0.94)	1992–1999	0.18 (–0.26 to 0.61)	1999–2009	–1.23* (–1.46 to –1.00)	2009–2019	–1.72* (–1.92 to –1.53)
IOGC	1990–1992	–4.04* (–6.22 to –1.81)	1992–2009	–2.06* (–2.15 to –1.98)	2009–2014	–3.33* (–4.03 to –2.62)	2014–2019	–1.84* (–2.34 to –1.33)
LOGC	1990–2003	–1.45* (–1.56 to –1.34)	2003–2007	–3.36* (–4.37 to –2.34)	2007–2014	–2.55* (–2.89 to –2.20)	2014–2019	–1.59* (–2.05 to –1.13)
Western Europe								
EOGC	1990–2003	–1.60* (–1.71 to –1.49)	2003–2009	–2.12* (–2.59 to –1.65)	2009–2016	–3.29* (–3.64 to –2.94)	2016–2019	–0.44 (–1.51 to 0.63)
IOGC	1990–1999	–3.16* (–3.29 to –3.04)	1999–2010	–2.07* (–2.18 to –1.96)	2010–2014	–3.29* (–3.98 to –2.60)	2014–2019	–1.65* (–1.97 to –1.34)
LOGC	1990–2003	–2.40* (–2.51 to –2.28)	2003–2006	–3.07* (–5.25 to –0.83)	2006–2014	–2.21* (–2.51 to –1.91)	2014–2019	–0.51 (–1.01 to 0.00)
Western sub-Saharan Africa								
EOGC	1990–1999	–1.59* (–1.70 to –1.49)	1999–2009	–1.09* (–1.19 to –0.98)	2009–2014	–0.29 (–0.66 to 0.09)	2014–2019	–1.21* (–1.47 to –0.95)
IOGC	1990–1992	–1.97* (–3.58 to –0.33)	1992–2002	–1.28* (–1.43 to –1.13)	2002–2014	–0.85* (–0.97 to –0.74)	2014–2019	–1.81* (–2.17 to –1.44)
LOGC	1990–1999	–1.10* (–1.19 to –1.02)	1999–2004	0.58* (0.28 to 0.87)	2004–2012	–0.15* (–0.28 to –0.03)	2012–2019	–1.42* (–1.54 to –1.29)

Abbreviations: CI, confidence interval; EAPC, estimated annual percentage change; GC, gastric cancer; EOGC, early-onset gastric cancer (15–49 years); IOGC, intermediate-onset gastric cancer (50–54 years); LOGC, later-onset gastric cancer (≥ 55 years). *Significantly different from 0 (*P* < .05).

Table S12. Temporal trends of incidence rates of gastric cancer in 203 countries, 1990–2019

Country	EOGC			IOGC			LOGC		
	**EAPC**	**LCI**	**UCI**	**EAPC**	**LCI**	**UCI**	**EAPC**	**LCI**	**UCI**
Afghanistan	0.00	-0.26	0.26	-0.57*	-0.84	-0.30	-0.09	-0.30	0.12
Albania	0.02	-0.33	0.37	-0.85*	-1.10	-0.59	-0.91*	-1.13	-0.70
Algeria	-1.08*	-1.30	-0.86	-1.98*	-2.14	-1.83	-1.66*	-1.78	-1.55
American Samoa	0.17*	0.05	0.29	-0.95*	-1.12	-0.78	-0.66*	-0.77	-0.55
Andorra	0.75*	0.56	0.95	-0.80*	-0.91	-0.70	-0.31	-0.41	-0.21
Angola	-2.16*	-2.25	-2.07	-2.44*	-2.53	-2.36	-1.88*	-1.95	-1.81
Antigua and Barbuda	-0.93*	-1.02	-0.83	-2.50*	-2.71	-2.29	-1.86*	-2.07	-1.66
Argentina	-1.01*	-1.16	-0.87	-0.99*	-1.13	-0.85	-1.22*	-1.33	-1.11
Armenia	-2.85*	-3.14	-2.55	-2.79*	-3.06	-2.52	-1.53*	-1.70	-1.36
Australia	-0.12	-0.24	0.01	-1.22*	-1.39	-1.06	-1.37*	-1.48	-1.27
Austria	-3.55*	-3.73	-3.37	-3.05*	-3.22	-2.88	-3.20*	-3.39	-3.02
Azerbaijan	-2.19*	-2.49	-1.88	-2.58*	-2.82	-2.33	-1.15*	-1.32	-0.99
Bahamas	-0.34*	-0.47	-0.22	-1.84*	-2.05	-1.64	-1.67*	-1.79	-1.55
Bahrain	-1.45*	-1.96	-0.94	-4.20*	-4.44	-3.97	-4.24*	-4.68	-3.80
Bangladesh	-1.23*	-1.37	-1.09	-1.76*	-1.94	-1.58	-1.75*	-1.94	-1.56
Barbados	-0.69*	-0.95	-0.42	-1.74*	-1.97	-1.51	-2.27*	-2.47	-2.07
Belarus	-3.16*	-3.48	-2.84	-3.89*	-4.20	-3.58	-2.75*	-2.99	-2.52
Belgium	-1.21*	-1.41	-1.02	-1.33*	-1.49	-1.17	-1.76*	-1.98	-1.54
Belize	0.61*	0.12	1.11	-0.13	-0.64	0.39	-1.39*	-1.92	-0.86
Benin	-1.12*	-1.20	-1.05	-1.19*	-1.29	-1.09	-0.74*	-0.90	-0.58
Bermuda	-1.12*	-1.29	-0.95	-2.56*	-2.83	-2.30	-2.03*	-2.21	-1.85
Bhutan	-1.22*	-1.39	-1.06	-1.61*	-1.73	-1.48	0.03	-0.05	0.10
Bolivia (Plurinational State of)	-1.68*	-1.79	-1.57	-2.06*	-2.17	-1.94	-1.02*	-1.11	-0.92
Bosnia and Herzegovina	-0.55*	-0.79	-0.31	-1.33*	-1.65	-1.01	-0.44*	-0.63	-0.26
Botswana	-1.03*	-1.63	-0.42	-1.86*	-2.49	-1.22	-1.42*	-1.79	-1.05
Brazil	-0.71*	-0.84	-0.59	-2.08*	-2.15	-2.01	-2.00*	-2.10	-1.90
Brunei Darussalam	-2.24*	-2.69	-1.79	-2.91*	-3.35	-2.46	-3.28*	-3.49	-3.06
Bulgaria	-0.68*	-1.10	-0.26	-0.85*	-1.19	-0.50	-0.62*	-0.99	-0.26
Burkina Faso	-0.71*	-0.99	-0.43	-0.75*	-1.02	-0.49	-0.54*	-0.77	-0.30
Burundi	-2.61*	-2.92	-2.31	-2.33*	-2.51	-2.15	-1.95*	-2.10	-1.80
Cabo Verde	-1.03*	-1.34	-0.72	-1.71*	-2.26	-1.16	-1.42*	-1.77	-1.06
Cambodia	-2.30*	-2.41	-2.20	-2.72*	-2.88	-2.56	-1.86*	-1.96	-1.75
Cameroon	-1.03*	-1.09	-0.98	-1.00*	-1.15	-0.86	-0.35*	-0.50	-0.21
Canada	-0.03	-0.23	0.16	-0.84*	-0.94	-0.74	-1.11*	-1.20	-1.02
Central African Republic	-0.92*	-1.00	-0.84	-1.11*	-1.18	-1.04	-1.23*	-1.28	-1.18
Chad	-0.53*	-0.66	-0.39	-0.23*	-0.35	-0.11	-0.02	-0.12	0.08
Chile	-0.52*	-0.69	-0.35	-2.18*	-2.32	-2.03	-1.70*	-1.81	-1.59
China	1.01*	0.73	1.30	-1.29*	-1.74	-0.85	-0.31	-0.65	0.03
Colombia	0.15*	0.00	0.30	-2.08*	-2.33	-1.84	-2.69*	-2.83	-2.54
Comoros	-0.83*	-1.25	-0.41	-1.72*	-1.97	-1.47	-1.30*	-1.42	-1.19
Congo	-1.99*	-2.21	-1.77	-3.04*	-3.23	-2.85	-2.21*	-2.34	-2.09
Cook Islands	0.24	-0.08	0.57	-1.20*	-1.47	-0.93	-1.31*	-1.45	-1.17
Costa Rica	-0.62*	-0.88	-0.35	-2.12*	-2.47	-1.76	-2.49*	-2.75	-2.23
Croatia	-2.47*	-2.65	-2.29	-2.76*	-2.97	-2.55	-1.65*	-1.76	-1.53
Cuba	-0.09	-0.19	0.00	-0.47*	-0.65	-0.28	-0.19*	-0.34	-0.04
Cyprus	1.38*	1.12	1.65	0.53*	0.07	0.99	0.59*	0.32	0.85
Czechia	-2.60*	-2.80	-2.40	-2.94*	-3.22	-2.65	-2.86*	-3.01	-2.72
Cote d'Ivoire	-1.09*	-1.16	-1.02	-1.64*	-1.76	-1.51	-0.79*	-0.89	-0.69
Democratic People's Republic of Korea	-0.88*	-0.94	-0.82	-1.40*	-1.48	-1.33	-0.51*	-0.68	-0.34
Democratic Republic of the Congo	-1.26*	-1.36	-1.16	-1.15*	-1.25	-1.06	-1.27*	-1.38	-1.17
Denmark	-2.46*	-2.72	-2.19	-2.13*	-2.54	-1.72	-1.70*	-1.97	-1.42
Djibouti	-0.30*	-0.45	-0.15	-1.16*	-1.31	-1.02	-0.84*	-0.94	-0.73
Dominica	-0.60*	-0.85	-0.36	-1.79*	-2.08	-1.50	-1.44*	-1.52	-1.36
Dominican Republic	1.24*	0.98	1.51	1.12*	0.94	1.30	1.25*	1.07	1.43
Ecuador	-0.28	-0.70	0.15	-1.29*	-1.53	-1.05	-0.95*	-1.16	-0.74

Country	EOGC			IOGC			LOGC		
	EAPC	LCI	UCI	EAPC	LCI	UCI	EAPC	LCI	UCI
Egypt	-1.01*	-1.19	-0.82	-0.11*	-0.34	0.11	-0.28*	-0.38	-0.18
El Salvador	-0.04	-0.36	0.29	-0.81*	-1.12	-0.50	-0.06	-0.47	0.35
Equatorial Guinea	-4.95*	-5.38	-4.51	-4.69*	-5.16	-4.21	-2.98*	-3.28	-2.68
Eritrea	-0.73*	-0.85	-0.62	-1.14*	-1.25	-1.03	-0.67*	-0.80	-0.53
Estonia	-2.12*	-2.41	-1.83	-2.98*	-3.24	-2.73	-1.69*	-1.87	-1.52
Eswatini	0.77*	0.12	1.42	-0.15	-0.77	0.47	-0.65*	-0.96	-0.33
Ethiopia	-4.71*	-4.94	-4.47	-4.53*	-4.70	-4.35	-2.89*	-2.99	-2.78
Fiji	-0.16	-0.53	0.21	-0.43*	-0.72	-0.14	-0.56*	-0.76	-0.36
Finland	-3.02*	-3.18	-2.86	-2.50*	-2.71	-2.30	-2.75*	-3.01	-2.49
France	-0.20*	-0.36	-0.03	-0.74*	-0.90	-0.57	-1.59*	-1.71	-1.47
Gabon	-2.02*	-2.22	-1.81	-2.20*	-2.38	-2.03	-1.75*	-1.88	-1.63
Gambia	-0.53*	-0.74	-0.32	-0.83*	-1.03	-0.63	-0.20*	-0.34	-0.06
Georgia	-0.70*	-1.05	-0.36	-0.56	-1.23	0.11	0.08	-0.34	0.50
Germany	-0.91*	-1.30	-0.53	-1.48*	-1.64	-1.32	-1.58*	-1.77	-1.39
Ghana	-1.86*	-2.01	-1.72	-1.82*	-1.98	-1.67	-1.22*	-1.35	-1.10
Greece	-0.41*	-0.50	-0.32	-1.18*	-1.27	-1.09	-1.23*	-1.35	-1.12
Greenland	-1.82*	-2.15	-1.48	-2.13*	-2.20	-2.06	-2.03*	-2.19	-1.87
Grenada	-0.89*	-1.06	-0.72	-1.58*	-1.99	-1.18	-1.92*	-2.19	-1.66
Guam	1.08*	0.67	1.48	0.38*	0.20	0.57	-1.25*	-1.62	-0.88
Guatemala	-1.14*	-1.81	-0.47	-0.57	-1.30	0.17	-0.05	-0.58	0.47
Guinea	-0.20*	-0.30	-0.11	0.12*	0.00	0.25	0.14*	0.00	0.29
Guinea-Bissau	-1.27*	-1.45	-1.09	-1.29*	-1.46	-1.12	-0.87*	-1.09	-0.65
Guyana	-0.18*	-0.55	0.19	-1.00*	-1.25	-0.75	-2.17*	-2.33	-2.02
Haiti	-1.43*	-1.70	-1.17	-1.56*	-1.75	-1.37	-1.27*	-1.38	-1.17
Honduras	-1.79*	-2.01	-1.56	0.11*	0.00	0.23	1.13*	0.92	1.33
Hungary	-3.43*	-3.80	-3.06	-2.15*	-2.39	-1.91	-2.44*	-2.54	-2.33
Iceland	-2.19*	-2.39	-2.00	-2.67*	-2.80	-2.54	-3.16*	-3.31	-3.02
India	-0.83*	-0.93	-0.73	-1.18*	-1.52	-0.84	-1.18*	-1.28	-1.08
Indonesia	-1.30*	-1.44	-1.15	-1.87*	-1.95	-1.78	-0.82*	-0.90	-0.74
Iran (Islamic Republic of)	-0.40*	-0.74	-0.05	-1.82*	-2.06	-1.58	-0.79*	-0.88	-0.70
Iraq	0.09	-0.09	0.27	-0.94*	-1.15	-0.74	-0.72*	-0.95	-0.49
Ireland	-0.40*	-0.60	-0.20	-1.91*	-2.03	-1.79	-1.87*	-1.99	-1.75
Israel	-0.83*	-1.14	-0.53	-1.28*	-1.46	-1.11	-1.95*	-2.18	-1.73
Italy	-0.73*	-0.83	-0.62	-1.83*	-1.88	-1.77	-1.44*	-1.50	-1.38
Jamaica	0.87*	0.28	1.46	-0.48*	-0.89	-0.07	-1.41*	-1.70	-1.11
Japan	-3.71*	-3.95	-3.47	-3.93*	-4.12	-3.73	-1.53*	-1.63	-1.43
Jordan	-0.09	-0.22	0.03	-2.20*	-2.32	-2.08	-1.43*	-1.51	-1.36
Kazakhstan	-3.21*	-3.48	-2.94	-3.52*	-3.70	-3.35	-2.63*	-2.77	-2.49
Kenya	0.42*	0.12	0.72	-0.04	-0.40	0.32	0.02	-0.20	0.23
Kiribati	-0.48*	-0.65	-0.31	-0.89*	-0.94	-0.83	-0.91*	-0.98	-0.84
Kuwait	-1.27*	-1.67	-0.87	-2.32*	-2.70	-1.94	-1.44*	-1.92	-0.95
Kyrgyzstan	-2.87*	-3.17	-2.57	-3.10*	-3.39	-2.82	-2.09*	-2.27	-1.91
Lao People's Democratic Republic	-3.28*	-3.40	-3.16	-3.86*	-3.99	-3.73	-2.76*	-2.85	-2.66
Latvia	-2.55*	-2.97	-2.13	-3.04*	-3.36	-2.71	-1.68*	-1.92	-1.44
Lebanon	-0.13	-0.44	0.18	-0.62*	-0.88	-0.36	0.17*	0.03	0.31
Lesotho	2.62*	2.21	3.04	1.60*	1.24	1.97	0.63*	0.40	0.87
Liberia	-1.32*	-1.72	-0.91	-1.51*	-1.75	-1.28	-1.08*	-1.24	-0.92
Libya	0.90*	0.44	1.35	-0.91*	-1.12	-0.70	-0.60*	-0.75	-0.45
Lithuania	-1.80*	-2.15	-1.45	-2.68*	-2.98	-2.38	-1.61*	-1.79	-1.43
Luxembourg	-2.77*	-2.92	-2.62	-3.04*	-3.30	-2.78	-2.41*	-2.54	-2.27
Madagascar	-1.32*	-1.46	-1.19	-1.25*	-1.36	-1.14	-1.28*	-1.47	-1.08
Malawi	-1.91*	-2.12	-1.71	-1.63*	-1.94	-1.32	-1.45*	-1.68	-1.22
Malaysia	-1.17*	-1.40	-0.95	-1.69*	-1.96	-1.43	-1.68*	-1.88	-1.47
Maldives	-4.01*	-4.53	-3.47	-5.58*	-6.06	-5.09	-2.95*	-3.13	-2.77
Mali	-1.76*	-1.90	-1.62	-1.29*	-1.45	-1.13	-0.71*	-0.77	-0.64
Malta	-1.23*	-1.42	-1.04	-1.94*	-2.14	-1.74	-2.13*	-2.27	-1.99

Country	EOGC			IOGC			LOGC		
	EAPC	LCI	UCI	EAPC	LCI	UCI	EAPC	LCI	UCI
Marshall Islands	0.50*	0.39	0.61	-0.70*	-0.79	-0.61	-1.41*	-1.52	-1.30
Mauritania	-2.35*	-2.56	-2.14	-2.67*	-2.98	-2.35	-1.36*	-1.59	-1.12
Mauritius	-1.06*	-1.26	-0.86	-2.52*	-2.71	-2.34	-2.65*	-2.83	-2.48
Mexico	0.81*	0.69	0.92	-0.49*	-0.57	-0.41	-1.55*	-1.65	-1.44
Micronesia (Federated States of)	-0.22*	-0.27	-0.17	-0.59*	-0.75	-0.43	-1.22*	-1.32	-1.12
Monaco	-0.94*	-1.19	-0.69	-1.46*	-1.71	-1.22	-1.39*	-1.55	-1.23
Mongolia	-0.24	-0.54	0.06	-2.37*	-2.63	-2.12	-2.40*	-2.64	-2.16
Montenegro	-1.12*	-1.62	-0.63	-0.60*	-0.95	-0.24	0.02	-0.20	0.23
Morocco	-0.46*	-0.57	-0.35	-0.80*	-0.95	-0.65	-0.72*	-0.82	-0.62
Mozambique	0.52*	0.22	0.82	0.78*	0.56	1.00	0.40*	0.29	0.50
Myanmar	-3.09*	-3.34	-2.83	-3.69*	-3.84	-3.53	-2.60*	-2.71	-2.49
Namibia	-0.72*	-1.19	-0.24	-1.28*	-1.63	-0.93	-0.68*	-0.89	-0.47
Nauru	-0.29	-0.62	0.03	-0.66*	-0.96	-0.36	-1.27	-1.57	-0.97
Nepal	-1.14*	-1.48	-0.80	-1.18*	-1.55	-0.81	-0.03	-0.28	0.23
Netherlands	-0.84	-1.09	-0.60	-1.70*	-1.86	-1.54	-1.42*	-1.58	-1.26
New Zealand	-0.71*	-0.82	-0.60	-1.65*	-1.83	-1.46	-1.95*	-2.14	-1.77
Nicaragua	-0.38*	-0.68	-0.07	-0.91*	-1.09	-0.73	-0.37*	-0.70	-0.04
Niger	-1.46*	-1.61	-1.30	-1.09*	-1.32	-0.87	-0.47*	-0.70	-0.25
Nigeria	-1.03*	-1.11	-0.95	-1.23*	-1.33	-1.12	-0.41*	-0.58	-0.24
Niue	-0.89*	-1.07	-0.71	-1.00*	-1.07	-0.92	-0.90*	-0.98	-0.83
North Macedonia	-1.78*	-2.01	-1.54	-2.22*	-2.51	-1.94	-1.24*	-1.38	-1.11
Northern Mariana Islands	-0.75*	-0.94	-0.57	-1.79*	-2.05	-1.52	-1.87*	-2.14	-1.59
Norway	-2.22*	-2.45	-1.99	-2.46*	-2.60	-2.32	-2.60*	-2.70	-2.50
Oman	-2.54*	-2.99	-2.08	-3.00*	-3.64	-2.35	-1.90*	-2.28	-1.52
Pakistan	0.22	-0.06	0.51	-0.15*	-0.50	0.20	-0.50*	-0.70	-0.29
Palau	1.17*	1.05	1.29	-0.56*	-0.78	-0.34	-1.18*	-1.31	-1.05
Palestine	-1.65*	-1.96	-1.34	-1.81*	-1.98	-1.63	-1.67*	-1.82	-1.53
Panama	0.00	-0.26	0.26	-1.43*	-1.80	-1.05	-1.51*	-1.65	-1.36
Papua New Guinea	0.29*	0.17	0.40	-0.10	-0.20	0.00	-0.20*	-0.32	-0.08
Paraguay	-1.17*	-1.61	-0.73	-1.05*	-1.47	-0.63	-1.22*	-1.67	-0.76
Peru	-0.44*	-0.68	-0.20	-1.51*	-1.77	-1.26	-0.59*	-0.79	-0.40
Philippines	-1.63*	-1.78	-1.49	-2.15*	-2.30	-1.99	-1.75*	-1.93	-1.57
Poland	-2.50*	-2.76	-2.23	-2.46*	-2.63	-2.28	-2.43*	-2.57	-2.29
Portugal	-1.18*	-1.57	-0.79	-0.96*	-1.18	-0.73	-2.10*	-2.19	-2.02
Puerto Rico	-1.67*	-1.83	-1.51	-2.22*	-2.38	-2.07	-2.53*	-2.70	-2.36
Qatar	-2.28*	-2.68	-1.87	-3.35*	-3.67	-3.02	-3.09*	-3.59	-2.59
Republic of Korea	-1.92*	-2.28	-1.56	-4.04*	-4.37	-3.71	-2.97*	-3.28	-2.66
Republic of Moldova	-2.08*	-2.39	-1.77	-2.16*	-2.56	-1.76	-1.47*	-1.88	-1.07
Romania	-0.61*	-0.85	-0.36	-1.15*	-1.34	-0.96	-0.69*	-0.89	-0.48
Russian Federation	-1.95*	-2.37	-1.53	-3.30*	-3.65	-2.94	-2.61*	-2.84	-2.38
Rwanda	-4.11*	-4.68	-3.54	-4.28*	-4.75	-3.80	-3.17*	-3.49	-2.85
Saint Kitts and Nevis	-2.61*	-2.93	-2.30	-2.80*	-3.19	-2.40	-2.80*	-3.08	-2.51
Saint Lucia	-0.16	-0.39	0.07	-2.29*	-2.75	-1.83	-2.19*	-2.50	-1.88
Saint Vincent and the Grenadines	0.02	-0.09	0.13	-0.44*	-0.78	-0.10	-1.71*	-1.93	-1.49
Samoa	0.38*	0.17	0.59	-0.92*	-0.99	-0.85	-0.99*	-1.02	-0.96
San Marino	-0.07	-0.17	0.02	-1.05*	-1.23	-0.86	-0.52*	-0.60	-0.45
Sao Tome and Principe	0.18	-0.09	0.45	-0.63*	-0.82	-0.44	-0.07	-0.33	0.19
Saudi Arabia	1.09*	0.79	1.39	-0.86*	-0.95	-0.77	-2.30*	-2.49	-2.12
Senegal	-0.99*	-1.29	-0.69	-1.09*	-1.41	-0.77	-0.37*	-0.64	-0.11
Serbia	-1.77*	-2.13	-1.40	-0.87*	-1.11	-0.63	-1.11*	-1.35	-0.86
Seychelles	-0.63*	-0.80	-0.46	-2.20*	-2.36	-2.05	-2.11*	-2.29	-1.93
Sierra Leone	-0.55*	-0.71	-0.39	-0.41*	-0.64	-0.18	-0.43*	-0.62	-0.24
Singapore	-2.81*	-2.99	-2.63	-3.49*	-3.59	-3.40	-3.52*	-3.66	-3.38
Slovakia	-1.69*	-1.88	-1.50	-2.44*	-2.64	-2.23	-1.87*	-2.10	-1.63
Slovenia	-1.79*	-1.98	-1.60	-2.64*	-2.75	-2.52	-1.78*	-1.86	-1.69
Solomon Islands	0.89*	0.77	1.02	-0.28*	-0.35	-0.21	-0.29*	-0.36	-0.22

Country	EOGC			IOGC			LOGC		
	EAPC	LCI	UCI	EAPC	LCI	UCI	EAPC	LCI	UCI
Somalia	-1.61*	-1.92	-1.30	-1.17*	-1.25	-1.09	-0.84*	-0.88	-0.80
South Africa	-3.48*	-3.88	-3.07	-2.04*	-2.51	-1.56	-1.38*	-1.86	-0.89
South Sudan	-0.69*	-0.93	-0.45	-1.60*	-1.70	-1.50	-1.48*	-1.52	-1.43
Spain	-0.69*	-0.81	-0.58	-1.62*	-1.73	-1.50	-1.20*	-1.42	-0.97
Sri Lanka	-1.25*	-1.51	-1.00	-1.71*	-1.91	-1.52	-1.42*	-1.55	-1.29
Sudan	-0.63*	-0.70	-0.56	-0.93*	-1.01	-0.85	-0.36*	-0.45	-0.27
Suriname	-0.39*	-0.67	-0.10	-1.32*	-1.75	-0.89	-1.29*	-1.60	-0.98
Sweden	-2.99*	-3.15	-2.83	-2.55*	-2.68	-2.43	-3.11*	-3.31	-2.90
Switzerland	-2.71*	-2.88	-2.54	-2.56*	-2.83	-2.28	-2.49*	-2.80	-2.17
Syrian Arab Republic	-0.07	-0.63	0.49	-1.31*	-1.66	-0.95	-0.63*	-0.85	-0.40
Tajikistan	-1.50*	-1.86	-1.14	-2.54*	-2.80	-2.27	-0.54*	-0.90	-0.18
Thailand	-1.21*	-1.76	-0.67	-2.93*	-3.24	-2.62	-2.53*	-2.72	-2.33
Timor-Leste	-2.53*	-3.02	-2.05	-2.19*	-2.57	-1.81	-0.76*	-1.04	-0.46
Togo	-0.19*	-0.34	-0.05	-0.67*	-0.81	-0.52	-0.52*	-0.67	-0.38
Tokelau	-0.57*	-0.66	-0.47	-1.37*	-1.40	-1.34	-1.46*	-1.60	-1.32
Tonga	0.40*	0.26	0.54	-0.57*	-0.61	-0.53	-0.21	-0.44	0.03
Trinidad and Tobago	-2.33*	-2.57	-2.09	-3.59*	-3.92	-3.26	-4.04*	-4.41	-3.66
Tunisia	0.44*	0.37	0.51	-0.80*	-0.87	-0.72	-0.67*	-0.79	-0.55
Turkey	-2.02*	-2.64	-1.40	-1.87*	-2.41	-1.32	-1.04*	-1.68	-0.41
Turkmenistan	-2.40*	-2.85	-1.94	-4.17*	-4.53	-3.80	-3.33*	-3.63	-3.04
Tuvalu	-0.75*	-0.81	-0.69	-1.15*	-1.25	-1.04	-1.30*	-1.37	-1.22
Uganda	-0.65*	-1.03	-0.26	-1.02*	-1.40	-0.63	-1.18*	-1.46	-0.89
Ukraine	-3.06*	-3.84	-2.27	-3.51*	-4.07	-2.95	-2.84*	-3.09	-2.58
United Arab Emirates	-0.55	-1.44	0.36	-2.25*	-2.48	-2.03	-3.00*	-3.42	-2.58
United Kingdom	-0.88*	-0.97	-0.78	-2.06*	-2.28	-1.84	-2.34*	-2.52	-2.16
United Republic of Tanzania	-0.84*	-0.94	-0.73	-1.29*	-1.34	-1.24	-1.01*	-1.07	-0.96
United States of America	-0.18	-0.39	0.04	-0.75*	-0.83	-0.67	-1.94*	-2.09	-1.79
United States Virgin Islands	-0.40*	-0.47	-0.33	0.68*	0.26	1.11	0.43*	0.12	0.74
Uruguay	-1.31*	-1.39	-1.22	-1.58*	-1.70	-1.45	-1.31*	-1.38	-1.24
Uzbekistan	-2.11*	-2.52	-1.71	-3.05*	-3.38	-2.71	-2.23*	-2.51	-1.96
Vanuatu	0.08	-0.06	0.22	-0.45*	-0.63	-0.27	-0.55*	-0.71	-0.40
Venezuela (Bolivarian Republic of)	0.25*	0.06	0.44	-1.38*	-1.58	-1.19	-1.81*	-2.02	-1.60
Viet Nam	0.19*	0.11	0.27	-1.71*	-1.78	-1.65	-2.61*	-2.86	-2.36
Yemen	-0.51*	-0.68	-0.33	-0.87*	-0.96	-0.79	-0.20*	-0.28	-0.13
Zambia	-1.17*	-1.39	-0.96	-1.53*	-1.89	-1.17	-1.46*	-1.77	-1.15
Zimbabwe	2.44*	1.96	2.93	1.12*	0.69	1.56	0.36*	0.10	0.61

EAPC, estimated annual percentage change; EOGC, early-onset gastric cancer (15–49 years); IOGC, intermediate-onset gastric cancer (50–54 years); LOGC, later-onset gastric cancer (≥ 55 years); LCI, lower limit of 95% confidence interval; UCI, upper limit of 95% confidence interval.

*Significantly different from 0 ($P < .05$).

Table S13. Temporal trends of death rates of gastric cancer in 203 countries, 1990–2019

Country	EOGC			IOGC			LOGC		
	EAPC	LCI	UCI	EAPC	LCI	UCI	EAPC	LCI	UCI
Afghanistan	0.06	-0.21	0.33	-0.60*	-0.87	-0.32	-0.03	-0.23	0.18
Albania	-0.46*	-0.85	-0.06	-1.26*	-1.52	-1.01	-1.20*	-1.42	-0.98
Algeria	-1.39*	-1.58	-1.20	-2.24*	-2.38	-2.10	-1.81*	-1.90	-1.72
American Samoa	0.06	-0.04	0.17	-1.08*	-1.26	-0.90	-0.66*	-0.77	-0.56
Andorra	-0.21*	-0.30	-0.11	-1.64*	-1.70	-1.57	-0.57*	-0.67	-0.47
Angola	-2.18*	-2.27	-2.10	-2.54*	-2.63	-2.46	-1.90*	-1.97	-1.83
Antigua and Barbuda	-1.24*	-1.34	-1.13	-2.77*	-2.98	-2.55	-2.17*	-2.37	-1.97
Argentina	-1.39*	-1.51	-1.27	-1.25*	-1.39	-1.12	-1.36*	-1.45	-1.26
Armenia	-3.09*	-3.40	-2.78	-3.02*	-3.27	-2.76	-1.51*	-1.71	-1.32
Australia	-1.08*	-1.17	-0.98	-2.09*	-2.33	-1.84	-2.15*	-2.29	-2.02
Austria	-4.52*	-4.67	-4.37	-3.98*	-4.16	-3.80	-3.97*	-4.20	-3.74
Azerbaijan	-2.23*	-2.51	-1.96	-2.69*	-2.92	-2.46	-1.16*	-1.37	-0.95
Bahamas	-0.57*	-0.70	-0.44	-1.99*	-2.20	-1.78	-1.80*	-1.93	-1.67
Bahrain	-2.16*	-2.69	-1.63	-4.71*	-4.92	-4.50	-4.70*	-5.17	-4.22
Bangladesh	-1.31*	-1.46	-1.17	-1.85*	-2.03	-1.68	-1.78*	-1.98	-1.58
Barbados	-0.98*	-1.24	-0.72	-2.02*	-2.25	-1.80	-2.57*	-2.78	-2.36
Belarus	-4.15*	-4.52	-3.79	-4.52*	-4.84	-4.20	-3.24*	-3.52	-2.96
Belgium	-2.29*	-2.45	-2.12	-2.20*	-2.34	-2.07	-2.37*	-2.65	-2.10
Belize	0.40	-0.10	0.91	-0.31	-0.82	0.21	-1.58*	-2.11	-1.04
Benin	-1.12*	-1.20	-1.04	-1.23*	-1.33	-1.13	-0.76*	-0.92	-0.60
Bermuda	-2.28*	-2.47	-2.09	-3.54*	-3.83	-3.25	-2.84*	-3.06	-2.62
Bhutan	-1.39*	-1.55	-1.24	-1.75*	-1.86	-1.63	0.10*	0.03	0.17
Bolivia (Plurinational State of)	-1.83*	-1.93	-1.73	-2.14*	-2.25	-2.03	-1.07*	-1.15	-0.99
Bosnia and Herzegovina	-0.95*	-1.21	-0.69	-1.67*	-1.98	-1.35	-0.58*	-0.77	-0.40
Botswana	-1.08*	-1.67	-0.49	-2.01*	-2.67	-1.35	-1.53*	-1.92	-1.15
Brazil	-1.06*	-1.18	-0.93	-2.35*	-2.41	-2.28	-2.18*	-2.27	-2.08
Brunei Darussalam	-2.84*	-3.29	-2.38	-3.44*	-3.89	-2.98	-3.72*	-3.93	-3.52
Bulgaria	-0.90*	-1.34	-0.47	-1.05*	-1.40	-0.71	-0.75*	-1.14	-0.35
Burkina Faso	-0.73*	-1.01	-0.44	-0.78*	-1.04	-0.51	-0.52*	-0.73	-0.30
Burundi	-2.57*	-2.87	-2.26	-2.37*	-2.55	-2.19	-2.02*	-2.17	-1.87
Cabo Verde	-1.19*	-1.49	-0.90	-1.86*	-2.39	-1.32	-1.44*	-1.76	-1.11
Cambodia	-2.45*	-2.56	-2.35	-2.86*	-3.01	-2.71	-1.87*	-1.96	-1.78
Cameroon	-1.05*	-1.11	-0.98	-1.05*	-1.21	-0.90	-0.35*	-0.50	-0.20
Canada	-1.33*	-1.47	-1.19	-2.09*	-2.20	-1.98	-1.92*	-2.07	-1.78
Central African Republic	-0.83*	-0.91	-0.76	-1.13*	-1.21	-1.06	-1.28*	-1.34	-1.22
Chad	-0.54*	-0.67	-0.41	-0.25*	-0.37	-0.12	-0.05*	-0.16	0.06
Chile	-1.41*	-1.57	-1.25	-2.83*	-2.94	-2.71	-2.15*	-2.27	-2.04
China	-1.09*	-1.51	-0.67	-2.94*	-3.46	-2.41	-1.47*	-1.88	-1.06
Colombia	-0.74*	-0.90	-0.58	-2.71*	-2.96	-2.47	-3.09*	-3.24	-2.95
Comoros	-0.79*	-1.20	-0.39	-1.76*	-2.01	-1.51	-1.29*	-1.40	-1.18
Congo	-1.92*	-2.14	-1.70	-3.11*	-3.31	-2.92	-2.22*	-2.34	-2.09
Cook Islands	-0.20	-0.53	0.13	-1.62*	-1.89	-1.34	-1.51*	-1.66	-1.36
Costa Rica	-1.48*	-1.75	-1.22	-2.71*	-3.06	-2.36	-2.93*	-3.20	-2.66
Croatia	-3.41*	-3.62	-3.20	-3.45*	-3.66	-3.24	-2.16*	-2.28	-2.04
Cuba	-0.60*	-0.70	-0.49	-0.93*	-1.10	-0.76	-0.63*	-0.78	-0.47
Cyprus	-0.43*	-0.61	-0.24	-0.84*	-1.26	-0.42	-0.56*	-0.77	-0.34
Czechia	-3.65*	-3.89	-3.41	-3.72*	-3.97	-3.47	-3.59*	-3.77	-3.40
Cote d'Ivoire	-1.07*	-1.14	-1.00	-1.67*	-1.80	-1.55	-0.72*	-0.82	-0.61
Democratic People's Republic of Korea	-0.93*	-1.03	-0.84	-1.52*	-1.62	-1.41	-0.51*	-0.69	-0.34
Democratic Republic of the Congo	-1.23*	-1.33	-1.12	-1.17*	-1.27	-1.07	-1.27*	-1.39	-1.15
Denmark	-3.64*	-3.87	-3.40	-3.05*	-3.44	-2.66	-2.45*	-2.72	-2.17
Djibouti	-0.26*	-0.42	-0.11	-1.19*	-1.34	-1.04	-0.83*	-0.93	-0.72
Dominica	-0.69*	-0.92	-0.47	-1.89*	-2.19	-1.58	-1.49*	-1.56	-1.41
Dominican Republic	1.07*	0.80	1.34	0.95*	0.77	1.13	1.14*	0.95	1.33
Ecuador	-0.67*	-1.08	-0.26	-1.46*	-1.71	-1.22	-1.26*	-1.48	-1.05

Country	EOGC			IOGC			LOGC		
	**EAPC**	**LCI**	**UCI**	**EAPC**	**LCI**	**UCI**	**EAPC**	**LCI**	**UCI**
Egypt	-1.21*	-1.37	-1.05	-0.26*	-0.48	-0.03	-0.45*	-0.55	-0.35
El Salvador	-0.66*	-0.97	-0.34	-1.26*	-1.56	-0.96	-0.31	-0.71	0.10
Equatorial Guinea	-5.03*	-5.45	-4.60	-4.84*	-5.32	-4.35	-2.93*	-3.22	-2.63
Eritrea	-0.63*	-0.74	-0.53	-1.26*	-1.38	-1.13	-0.69*	-0.83	-0.56
Estonia	-3.99*	-4.34	-3.65	-4.23*	-4.52	-3.95	-2.47*	-2.62	-2.32
Eswatini	0.71*	0.10	1.33	-0.17	-0.82	0.49	-0.68*	-1.00	-0.36
Ethiopia	-4.73*	-4.97	-4.49	-4.58*	-4.75	-4.41	-2.76*	-2.86	-2.65
Fiji	-0.21	-0.57	0.15	-0.50*	-0.79	-0.21	-0.64*	-0.85	-0.44
Finland	-4.20*	-4.34	-4.06	-3.23*	-3.43	-3.02	-3.33*	-3.63	-3.03
France	-1.50*	-1.63	-1.38	-1.79*	-1.91	-1.66	-2.32*	-2.51	-2.14
Gabon	-2.00*	-2.22	-1.78	-2.28*	-2.45	-2.10	-1.79*	-1.93	-1.65
Gambia	-0.53*	-0.74	-0.33	-0.85*	-1.06	-0.65	-0.12	-0.25	0.01
Georgia	-0.67*	-1.02	-0.32	-0.57	-1.24	0.10	0.26	-0.17	0.70
Germany	-1.86*	-2.26	-1.47	-2.15*	-2.31	-1.99	-1.80*	-2.04	-1.56
Ghana	-1.90*	-2.05	-1.75	-1.87*	-2.02	-1.72	-1.18*	-1.29	-1.06
Greece	-1.12*	-1.25	-0.98	-1.78*	-1.90	-1.65	-1.54*	-1.66	-1.41
Greenland	-2.04*	-2.41	-1.67	-2.36*	-2.43	-2.28	-2.19*	-2.35	-2.03
Grenada	-1.03*	-1.21	-0.86	-1.73*	-2.15	-1.31	-2.23*	-2.51	-1.96
Guam	1.08*	0.75	1.41	0.30*	0.08	0.51	-1.18*	-1.58	-0.77
Guatemala	-1.50*	-2.14	-0.85	-0.79*	-1.51	-0.07	-0.14	-0.64	0.37
Guinea	-0.22*	-0.31	-0.13	0.11	-0.02	0.24	0.16	-0.01	0.32
Guinea-Bissau	-1.26*	-1.44	-1.08	-1.33*	-1.50	-1.16	-0.88*	-1.10	-0.65
Guyana	-0.18	-0.56	0.20	-1.06*	-1.31	-0.81	-2.24*	-2.38	-2.09
Haiti	-1.46*	-1.72	-1.19	-1.59*	-1.79	-1.40	-1.26*	-1.36	-1.17
Honduras	-1.98*	-2.20	-1.76	-0.07	-0.19	0.05	1.00*	0.79	1.22
Hungary	-3.91*	-4.27	-3.55	-2.55*	-2.78	-2.31	-2.75*	-2.85	-2.65
Iceland	-3.17*	-3.27	-3.06	-3.50*	-3.67	-3.33	-3.70*	-3.80	-3.60
India	-0.90*	-0.99	-0.80	-1.26*	-1.59	-0.92	-1.16*	-1.27	-1.06
Indonesia	-1.41*	-1.57	-1.26	-1.99*	-2.09	-1.90	-0.82*	-0.91	-0.74
Iran (Islamic Republic of)	-0.79*	-1.13	-0.45	-2.14*	-2.37	-1.91	-0.87*	-0.94	-0.80
Iraq	-0.23*	-0.38	-0.08	-1.16*	-1.34	-0.97	-0.96*	-1.16	-0.76
Ireland	-2.11*	-2.26	-1.96	-3.25*	-3.38	-3.13	-2.90*	-3.05	-2.76
Israel	-1.90*	-2.20	-1.60	-2.12*	-2.26	-1.97	-2.54*	-2.77	-2.31
Italy	-1.96*	-2.01	-1.90	-2.84*	-2.93	-2.75	-2.19*	-2.27	-2.10
Jamaica	0.59*	0.03	1.16	-0.69*	-1.11	-0.28	-1.56*	-1.85	-1.27
Japan	-4.39*	-4.65	-4.13	-4.23*	-4.43	-4.03	-1.38*	-1.50	-1.27
Jordan	-0.70*	-0.81	-0.60	-2.63*	-2.75	-2.52	-1.74*	-1.82	-1.66
Kazakhstan	-3.43*	-3.74	-3.12	-3.71*	-3.91	-3.52	-2.79*	-2.96	-2.62
Kenya	1.00*	0.69	1.31	0.42*	0.04	0.81	0.32*	0.08	0.56
Kiribati	-0.47*	-0.64	-0.29	-0.98*	-1.04	-0.92	-1.00*	-1.07	-0.93
Kuwait	-2.04*	-2.47	-1.60	-2.94*	-3.36	-2.53	-1.76*	-2.30	-1.22
Kyrgyzstan	-2.96*	-3.25	-2.66	-3.20*	-3.48	-2.93	-2.14*	-2.29	-1.98
Lao People's Democratic Republic	-3.37*	-3.49	-3.25	-3.95*	-4.08	-3.83	-2.74*	-2.83	-2.66
Latvia	-3.44*	-3.85	-3.03	-3.53*	-3.86	-3.20	-2.09*	-2.32	-1.85
Lebanon	-1.31*	-1.56	-1.06	-1.45*	-1.66	-1.24	-0.44*	-0.56	-0.32
Lesotho	2.55*	2.15	2.96	1.64*	1.25	2.03	0.60*	0.34	0.86
Liberia	-1.32*	-1.74	-0.89	-1.56*	-1.80	-1.32	-1.05*	-1.21	-0.90
Libya	0.62*	0.17	1.07	-1.15*	-1.37	-0.93	-0.75*	-0.91	-0.60
Lithuania	-2.35*	-2.71	-1.98	-3.07*	-3.38	-2.76	-1.90*	-2.08	-1.72
Luxembourg	-4.02*	-4.16	-3.88	-4.02*	-4.26	-3.78	-3.09*	-3.14	-3.03
Madagascar	-1.24*	-1.38	-1.11	-1.24*	-1.36	-1.12	-1.37*	-1.57	-1.18
Malawi	-1.90*	-2.10	-1.70	-1.66*	-1.97	-1.35	-1.42*	-1.65	-1.19
Malaysia	-1.74*	-1.95	-1.53	-2.14*	-2.40	-1.88	-2.01*	-2.22	-1.80
Maldives	-5.03*	-5.49	-4.56	-6.36*	-6.81	-5.91	-3.20*	-3.34	-3.05
Mali	-1.79*	-1.93	-1.65	-1.32*	-1.48	-1.16	-0.67*	-0.73	-0.61
Malta	-2.42*	-2.62	-2.22	-2.84*	-3.03	-2.65	-2.78*	-2.93	-2.62

Country	EOGC			IOGC			LOGC		
	EAPC	LCI	UCI	EAPC	LCI	UCI	EAPC	LCI	UCI
Marshall Islands	0.49*	0.36	0.61	-0.75*	-0.85	-0.66	-1.59*	-1.70	-1.49
Mauritania	-2.39*	-2.59	-2.19	-2.73*	-3.04	-2.42	-1.30*	-1.52	-1.08
Mauritius	-1.40*	-1.61	-1.20	-2.82*	-3.00	-2.65	-2.83*	-3.00	-2.66
Mexico	0.27*	0.16	0.38	-0.89*	-0.97	-0.81	-1.87*	-1.98	-1.76
Micronesia (Federated States of)	-0.35*	-0.41	-0.30	-0.70*	-0.86	-0.54	-1.36*	-1.45	-1.28
Monaco	-1.79*	-2.07	-1.51	-2.17*	-2.45	-1.89	-1.87*	-2.04	-1.69
Mongolia	-0.30	-0.61	0.02	-2.45*	-2.71	-2.19	-2.51*	-2.76	-2.25
Montenegro	-1.48*	-2.04	-0.91	-0.91*	-1.31	-0.51	-0.19	-0.44	0.06
Morocco	-0.58*	-0.71	-0.45	-0.90*	-1.05	-0.76	-0.73*	-0.82	-0.63
Mozambique	0.48*	0.18	0.78	0.76*	0.54	0.98	0.35*	0.25	0.45
Myanmar	-3.19*	-3.46	-2.93	-3.82*	-3.98	-3.67	-2.58*	-2.70	-2.46
Namibia	-0.76*	-1.23	-0.29	-1.38*	-1.75	-1.01	-0.68*	-0.92	-0.45
Nauru	-0.43*	-0.79	-0.06	-0.75*	-1.08	-0.42	-1.46*	-1.78	-1.14
Nepal	-1.22*	-1.55	-0.89	-1.26*	-1.63	-0.89	0.01	-0.23	0.26
Netherlands	-2.06*	-2.30	-1.82	-2.71*	-2.88	-2.55	-2.11*	-2.29	-1.93
New Zealand	-1.65*	-1.76	-1.55	-2.46*	-2.62	-2.29	-2.53*	-2.69	-2.36
Nicaragua	-1.05*	-1.31	-0.79	-1.42*	-1.60	-1.24	-0.77*	-1.16	-0.37
Niger	-1.46*	-1.62	-1.30	-1.13*	-1.36	-0.90	-0.45*	-0.66	-0.23
Nigeria	-1.02*	-1.09	-0.94	-1.28*	-1.38	-1.18	-0.37*	-0.54	-0.19
Niue	-1.36*	-1.54	-1.17	-1.38*	-1.45	-1.30	-1.25*	-1.31	-1.19
North Macedonia	-2.17*	-2.42	-1.92	-2.54*	-2.83	-2.25	-1.47*	-1.60	-1.33
Northern Mariana Islands	-0.85*	-1.04	-0.66	-1.98*	-2.25	-1.71	-2.10*	-2.37	-1.83
Norway	-3.52*	-3.67	-3.38	-3.54*	-3.62	-3.46	-3.27*	-3.38	-3.16
Oman	-3.33*	-3.78	-2.88	-3.51*	-4.15	-2.86	-2.28*	-2.66	-1.91
Pakistan	0.18	-0.11	0.48	-0.20	-0.56	0.16	-0.63*	-0.82	-0.44
Palau	0.93*	0.82	1.03	-0.83*	-1.03	-0.63	-1.48*	-1.61	-1.36
Palestine	-1.85*	-2.11	-1.58	-1.98*	-2.14	-1.82	-1.84*	-1.97	-1.72
Panama	-0.61*	-0.92	-0.30	-1.88*	-2.27	-1.49	-1.71*	-1.85	-1.57
Papua New Guinea	0.32*	0.20	0.44	-0.11	-0.22	0.00	-0.21*	-0.35	-0.07
Paraguay	-1.44*	-1.88	-1.00	-1.26*	-1.68	-0.83	-1.41*	-1.87	-0.94
Peru	-0.88*	-1.14	-0.63	-1.72*	-1.98	-1.46	-1.10*	-1.31	-0.88
Philippines	-1.77*	-1.91	-1.62	-2.23*	-2.39	-2.08	-1.82*	-2.00	-1.65
Poland	-2.85*	-3.13	-2.57	-2.70*	-2.86	-2.55	-2.50*	-2.64	-2.36
Portugal	-2.53*	-2.83	-2.23	-1.97*	-2.13	-1.82	-2.46*	-2.56	-2.37
Puerto Rico	-2.59*	-2.78	-2.40	-2.97*	-3.15	-2.79	-3.13*	-3.33	-2.92
Qatar	-3.54*	-4.01	-3.08	-4.20*	-4.55	-3.84	-3.90*	-4.41	-3.38
Republic of Korea	-4.63*	-4.83	-4.42	-6.50*	-6.81	-6.18	-4.82*	-5.04	-4.61
Republic of Moldova	-2.48*	-2.82	-2.15	-2.41*	-2.82	-2.01	-1.68*	-2.07	-1.29
Romania	-1.05*	-1.30	-0.79	-1.54*	-1.74	-1.34	-0.97*	-1.18	-0.75
Russian Federation	-3.13*	-3.66	-2.59	-4.12*	-4.51	-3.72	-3.02*	-3.30	-2.74
Rwanda	-4.04*	-4.59	-3.49	-4.39*	-4.89	-3.90	-3.22*	-3.55	-2.89
Saint Kitts and Nevis	-2.97*	-3.29	-2.64	-3.10*	-3.51	-2.70	-3.14*	-3.42	-2.87
Saint Lucia	-0.38*	-0.63	-0.13	-2.51*	-2.98	-2.04	-2.35*	-2.68	-2.03
Saint Vincent and the Grenadines	-0.06	-0.19	0.07	-0.54*	-0.90	-0.19	-1.77*	-1.98	-1.55
Samoa	0.29*	0.09	0.49	-1.02*	-1.11	-0.94	-1.01*	-1.04	-0.98
San Marino	-0.82*	-0.96	-0.67	-1.72*	-1.94	-1.51	-0.73*	-0.80	-0.67
Sao Tome and Principe	0.12	-0.14	0.39	-0.70*	-0.89	-0.51	-0.06	-0.35	0.23
Saudi Arabia	-0.56*	-0.74	-0.37	-2.24*	-2.30	-2.17	-3.33*	-3.52	-3.13
Senegal	-0.99*	-1.29	-0.70	-1.13*	-1.45	-0.81	-0.35*	-0.61	-0.09
Serbia	-2.66*	-3.08	-2.24	-1.61*	-1.85	-1.36	-1.38*	-1.63	-1.12
Seychelles	-0.99*	-1.15	-0.82	-2.51*	-2.67	-2.35	-2.36*	-2.54	-2.18
Sierra Leone	-0.59*	-0.75	-0.43	-0.44*	-0.67	-0.21	-0.47*	-0.65	-0.28
Singapore	-4.79*	-5.06	-4.53	-5.30*	-5.46	-5.14	-4.87*	-5.08	-4.67
Slovakia	-2.55*	-2.74	-2.37	-3.03*	-3.23	-2.84	-2.58*	-2.77	-2.39
Slovenia	-3.49*	-3.74	-3.24	-4.01*	-4.15	-3.87	-2.51*	-2.61	-2.41
Solomon Islands	0.86*	0.72	0.99	-0.34*	-0.42	-0.26	-0.28*	-0.36	-0.21

Country	EOGC			IOGC			LOGC		
	EAPC	LCI	UCI	EAPC	LCI	UCI	EAPC	LCI	UCI
Somalia	-1.47*	-1.77	-1.17	-1.06*	-1.15	-0.97	-0.84*	-0.88	-0.79
South Africa	-3.53*	-3.93	-3.12	-2.07*	-2.56	-1.57	-1.46*	-1.96	-0.96
South Sudan	-0.60*	-0.84	-0.36	-1.63*	-1.73	-1.53	-1.50*	-1.56	-1.44
Spain	-2.04*	-2.22	-1.86	-2.65*	-2.77	-2.53	-1.96*	-2.24	-1.68
Sri Lanka	-1.93*	-2.21	-1.64	-2.29*	-2.51	-2.07	-1.88*	-2.00	-1.75
Sudan	-0.93*	-1.00	-0.86	-1.08*	-1.16	-1.00	-0.39*	-0.50	-0.29
Suriname	-0.50*	-0.78	-0.22	-1.46*	-1.88	-1.04	-1.37*	-1.68	-1.06
Sweden	-3.95*	-4.15	-3.75	-3.29*	-3.40	-3.18	-3.49*	-3.72	-3.27
Switzerland	-3.64*	-3.88	-3.39	-3.33*	-3.68	-2.97	-3.05*	-3.43	-2.66
Syrian Arab Republic	-0.46	-1.04	0.12	-1.63*	-1.98	-1.27	-0.91*	-1.13	-0.69
Tajikistan	-1.49*	-1.83	-1.14	-2.57*	-2.83	-2.31	-0.55*	-0.93	-0.17
Thailand	-1.92*	-2.46	-1.37	-3.52*	-3.83	-3.21	-2.87*	-3.06	-2.67
Timor-Leste	-2.70*	-3.17	-2.23	-2.30*	-2.68	-1.92	-0.70*	-0.99	-0.42
Togo	-0.17*	-0.32	-0.02	-0.71*	-0.85	-0.57	-0.55*	-0.70	-0.39
Tokelau	-0.83*	-0.94	-0.72	-1.57*	-1.60	-1.55	-1.57*	-1.75	-1.39
Tonga	0.28*	0.14	0.41	-0.67*	-0.71	-0.63	-0.15	-0.38	0.09
Trinidad and Tobago	-2.56*	-2.81	-2.31	-3.79*	-4.12	-3.45	-4.19*	-4.57	-3.81
Tunisia	-0.13*	-0.22	-0.04	-1.23*	-1.30	-1.16	-0.98*	-1.13	-0.83
Turkey	-2.78*	-3.36	-2.20	-2.43*	-2.94	-1.92	-1.43*	-2.01	-0.85
Turkmenistan	-2.49*	-2.93	-2.04	-4.27*	-4.63	-3.91	-3.44*	-3.72	-3.16
Tuvalu	-0.91*	-0.98	-0.84	-1.25*	-1.37	-1.14	-1.29*	-1.36	-1.22
Uganda	-0.65*	-1.03	-0.27	-1.11*	-1.49	-0.72	-1.20*	-1.46	-0.94
Ukraine	-3.52*	-4.32	-2.72	-3.86*	-4.43	-3.29	-3.31*	-3.61	-3.02
United Arab Emirates	-0.76	-1.68	0.17	-2.40*	-2.63	-2.18	-3.27*	-3.70	-2.84
United Kingdom	-1.85*	-1.94	-1.76	-2.81*	-3.04	-2.58	-2.71*	-2.89	-2.53
United Republic of Tanzania	-0.79*	-0.90	-0.69	-1.33*	-1.38	-1.28	-1.00*	-1.06	-0.95
United States of America	-0.87*	-1.02	-0.72	-1.42*	-1.57	-1.28	-2.45*	-2.59	-2.31
United States Virgin Islands	-0.75*	-0.84	-0.66	0.40	-0.05	0.84	0.12	-0.20	0.44
Uruguay	-1.73*	-1.81	-1.66	-1.87*	-1.99	-1.74	-1.44*	-1.50	-1.38
Uzbekistan	-2.18*	-2.60	-1.76	-3.13*	-3.47	-2.80	-2.39*	-2.69	-2.09
Vanuatu	0.06	-0.08	0.20	-0.48*	-0.65	-0.30	-0.58*	-0.73	-0.43
Venezuela (Bolivarian Republic of)	-0.48*	-0.70	-0.26	-1.86*	-2.08	-1.65	-2.22*	-2.44	-2.00
Viet Nam	-0.30*	-0.41	-0.19	-2.15*	-2.21	-2.09	-2.92*	-3.18	-2.66
Yemen	-0.63*	-0.82	-0.44	-0.95*	-1.04	-0.86	-0.19*	-0.28	-0.11
Zambia	-1.15*	-1.36	-0.95	-1.57*	-1.93	-1.20	-1.48*	-1.80	-1.16
Zimbabwe	2.78*	2.32	3.25	1.30*	0.92	1.68	0.44*	0.21	0.66

EAPC, estimated annual percentage change; EOGC, early-onset gastric cancer (15–49 years); IOGC, intermediate-onset gastric cancer (50–54 years); LOGC, later-onset gastric cancer (≥ 55 years); LCI, lower limit of 95% confidence interval; UCI, upper limit of 95% confidence interval.

*Significantly different from 0 ($P < .05$).

Table S14. Incidence (2004–2019) and prediction (2020–2035) of gastric cancer globally and in 12 countries

Location	Population (annual, million)		Incidence number (95% UI)		Incidence rate (per 100 000)		EAPC (95% CI)	
	2019	2035	2019	2035	2019	2035	2004 to 2019	2020 to 2035
Global								
EOGC	3903.8	4302.8	146296	195971	3.7	4.6	–1.51* (–1.71 to 1.30)	1.44* (1.27 to 1.61)
IOGC	438.3	505.5	92631	106595	21.1	21.1	–2.56* (–3.25 to –1.87)	–0.01 (–0.46 to 0.45)
LOGC	1391.6	1997.7	1032861	1581142	74.0	78.0	–1.85* (–1.99 to –1.70)	0.43* (0.28 to 0.58)
America								
Brazil								
EOGC	114.0	109.4	3031	3088	2.7	2.8	–1.16* (–1.25 to –1.06)	0.37* (0.31 to 0.42)
IOGC	12.7	16.5	1811	2077	14.4	12.6	–2.39* (–2.52 to –2.25)	–0.69* (–0.74 to –0.64)
LOGC	40.0	62.8	19126	25953	48.1	41.3	–2.36* (–2.53 to –2.18)	–0.82* (–0.88 to –0.77)
Canada								
EOGC	17.1	18.5	342	456	2.0	2.5	–1.31* (–1.53 to –1.10)	1.38* (1.34 to 1.42)
IOGC	2.5	2.8	237	307	9.6	11.2	–0.86* (–1.07 to –0.65)	0.94* (0.82 to 1.07)
LOGC	11.9	14.9	4833	7712	41.0	51.9	–0.87* (–1.11 to –0.62)	1.63* (1.55 to 1.70)
USA								
EOGC	151.7	160.6	2560	3203	1.7	2.0	–0.99* (–1.14 to –0.84)	1.09* (1.04 to 1.13)
IOGC	20.9	21.4	1663	2032	8.0	9.5	–0.39* (–0.49 to –0.30)	1.18* (1.07 to 1.30)
LOGC	95.5	114.3	27882	39943	29.4	34.9	–1.43* (–1.73 to –1.12)	1.26* (1.19 to 1.34)
Asia								
China								
EOGC	772.4	641.3	66744	112644	9.2	17.6	–0.50* (–0.88 to –0.12)	4.52* (4.17 to 4.88)
IOGC	120.7	97.5	49679	58039	41.2	59.6	–3.29* (–4.15 to –2.41)	2.81* (1.79 to 3.85)
LOGC	335.4	506.7	496386	931738	148.0	183.9	–2.19* (–2.41 to –1.97)	1.36* (0.91 to 1.80)
Japan								
EOGC	51.5	40.7	3619	2876	7.1	7.1	–2.52* (–2.76 to –2.29)	0.06 (–0.20 to 0.33)
IOGC	8.4	7.6	2460	1933	29.9	25.5	–4.53* (–4.92 to –4.13)	–0.60* (–0.90 to –0.30)
LOGC	50.9	56.0	96060	102858	188.6	183.7	–1.10* (–1.22 to –0.99)	–0.31* (–0.37 to –0.24)
Korea								
EOGC	24.9	19.1	2715	4085	10.9	21.4	–3.19* (–3.79 to –2.60)	4.15* (3.76 to 4.55)
IOGC	4.3	3.7	1672	2684	38.8	71.8	–4.06* (–4.64 to –3.48)	3.99* (3.57 to 4.42)
LOGC	15.5	22.7	20653	49321	134.0	217.6	–3.93* (–4.44 to –3.43)	3.20* (2.71 to 3.69)
Europe								

France								
EOGC	27.7	27.1	557	481	2.0	1.8	–0.96* (–1.01 to –0.91)	–0.72* (–0.79 to –0.65)
IOGC	4.4	4.1	405	380	9.3	9.3	–1.37* (–1.48 to –1.26)	–0.03* (–0.05 to –0.01)
LOGC	21.5	25.3	8446	10368	39.6	40.9	–0.82* (–0.98 to –0.67)	0.32* (0.19 to 0.44)
Germany								
EOGC	34.7	32.2	887	1103	2.6	3.4	–2.79* (–3.19 to –2.39)	2.15* (2.04 to 2.26)
IOGC	6.9	5.4	850	848	12.4	15.6	–2.40* (–2.62 to –2.18)	1.66* (1.37 to 1.95)
LOGC	30.4	33.5	17304	23488	57.0	70.1	–2.35* (–2.76 to –1.94)	1.55* (1.29 to 1.82)
Italy								
EOGC	25.3	21.3	856	557	3.4	2.6	–1.24* (–1.41 to –1.07)	–1.71* (–1.78 to –1.64)
IOGC	4.9	3.7	686	485	14.0	13.3	–1.85* (–1.98 to –1.71)	–0.18* (–0.26 to –0.10)
LOGC	22.3	26.8	16874	19707	75.7	73.6	–1.58* (–1.73 to –1.42)	–0.13 (–0.29 to 0.02)
Sweden								
EOGC	4.4	4.6	42	41	1.0	0.9	–2.28* (–2.68 to –1.89)	–0.16 (–0.35 to 0.04)
IOGC	0.67	0.64	36	33	5.4	5.3	–2.54* (–2.84 to –2.24)	–0.18* (–0.29 to –0.08)
LOGC	3.2	3.8	959	1177	30.5	31.2	–1.93* (–2.35 to –1.50)	0.24* (0.13 to 0.35)
UK								
EOGC	30.1	30.6	476	640	1.6	2.1	–0.75* (–1.06 to –0.44)	1.83* (1.79 to 1.86)
IOGC	4.7	4.6	337	448	7.2	9.8	–1.18* (–1.62 to –0.74)	1.86* (1.77 to 1.96)
LOGC	20.8	24.8	8651	12509	12.9	17.5	–0.83* (–1.27 to –0.39)	2.04* (1.93 to 2.15)
Oceania								
Australia								
EOGC	11.8	13.2	206	234	1.7	1.8	–0.59* (–0.84 to –0.34)	0.14* (0.05 to 0.23)
IOGC	1.6	1.9	128	173	8.2	9.3	–0.35* (–0.62 to –0.08)	0.69* (0.67 to 0.72)
LOGC	6.9	9.2	2563	3830	37.3	41.5	–1.08* (–1.35 to –0.80)	0.79* (0.69 to 0.89)

CI, confidence interval; EAPC, estimated annual percentage change; GC, gastric cancer; UI, uncertainty interval;

EOGC, early-onset gastric cancer (15–49 years); IOGC, intermediate-onset gastric cancer (50–54 years); LOGC, later-onset gastric cancer (≥ 55 years).

*Significantly different from 0 (*P* < .05).
